# Comprehensive characterization of tissue-specific chromatin accessibility in L2 *Caenorhabditis elegans* nematodes

**DOI:** 10.1101/gr.271791.120

**Published:** 2021-10

**Authors:** Timothy J. Durham, Riza M. Daza, Louis Gevirtzman, Darren A. Cusanovich, Olubusayo Bolonduro, William Stafford Noble, Jay Shendure, Robert H. Waterston

**Affiliations:** 1 Department of Genome Sciences, University of Washington, Seattle, Washington 98195, USA;; 2 Department of Cellular and Molecular Medicine, University of Arizona, Tucson, Arizona 85721, USA;; 3 Paul G. Allen School of Computer Science and Engineering, University of Washington, Seattle, Washington 98195, USA;; 4 Howard Hughes Medical Institute, Chevy Chase, Maryland 20815, USA;; 5 Brotman Baty Institute for Precision Medicine, Seattle, Washington 98195, USA;; 6 Allen Discovery Center for Cell Lineage Tracing, University of Washington, Seattle, Washington 98195, USA

## Abstract

Recently developed single-cell technologies allow researchers to characterize cell states at ever greater resolution and scale. *Caenorhabditis elegans* is a particularly tractable system for studying development, and recent single-cell RNA-seq studies characterized the gene expression patterns for nearly every cell type in the embryo and at the second larval stage (L2). Gene expression patterns give insight about gene function and into the biochemical state of different cell types; recent advances in other single-cell genomics technologies can now also characterize the regulatory context of the genome that gives rise to these gene expression levels at a single-cell resolution. To explore the regulatory DNA of individual cell types in *C. elegans*, we collected single-cell chromatin accessibility data using the sci-ATAC-seq assay in L2 larvae to match the available single-cell RNA-seq data set. By using a novel implementation of the latent Dirichlet allocation algorithm, we identify 37 clusters of cells that correspond to different cell types in the worm, providing new maps of putative cell type–specific gene regulatory sites, with promise for better understanding of cellular differentiation and gene regulation.

Critical cellular processes are dependent on fine-tuned control of gene expression levels. From properly responding to environmental stimuli to progressing through the stages of development and differentiation, specific changes in gene expression play an important role in facilitating precise changes in cellular state. Recent advances in single-cell transcriptomics have enabled the massively parallel measurement of gene expression in individual cells, giving unprecedented genome-wide insight into which genes are regulated together in the same cell and into expression dynamics over time. Determining which genes are important in which cells and under which conditions is critical to attaining a deeper understanding of gene function. However, in order to truly understand how gene expression reflects and influences cell state, we must also understand how it is controlled. The nematode *Caenorhabditis elegans* is a particularly powerful system in which to apply single-cell genomics technologies because it has limited cell numbers that nonetheless form diverse tissue and cell types, it is very amenable to genetic manipulation, and the developmental lineage of every cell is known and invariant. In the past few years, the worm has been the subject of perhaps the most comprehensive cell type–specific metazoan gene expression atlas by single-cell RNA-seq (scRNA-seq) (Cao et al. 2017; Packer et al. 2019). These studies of the *C. elegans* embryo (Packer et al. 2019) and second larval stage (L2) (Cao et al. 2017) provide a survey of the full complement of genes expressed in each major cell type, and even some cells present only once in the worm (e.g., the ASEL and ASER gustatory neurons). Now, to understand how these tissue-specific expression patterns arise, we also need to have a similarly comprehensive catalog of regulatory elements to map their activity in different cell types and at different stages of the life cycle.

Several efforts have been undertaken to map regulatory DNA in the worm (Araya et al. 2014; Daugherty et al. 2017; Ho et al. 2017; Jänes et al. 2018; Kudron et al. 2018). Collectively, these studies have identified tens of thousands of chromatin accessibility regions and transcription factor (TF) binding sites, using DNase-seq (Ho et al. 2017), ATAC-seq (Daugherty et al. 2017; Jänes et al. 2018), and ChIP-seq (Araya et al. 2014; Kudron et al. 2018) to assay developmental stages throughout the worm life cycle. The results show that the activity at many regulatory sites changes over the worm's life span. However, the data from all of these studies are from whole worms and thus do not resolve differences in regulatory activity across cell types. The lack of cell type resolution is problematic for three main reasons. First, gene regulation is often highly cell type–specific, and even when different cell types express the same gene, they may use different enhancers or promoters to regulate that gene. In the case of two sites regulating the same gene in different cell types at the same stage, a whole-worm chromatin accessibility data set would only show that both sites are accessible at the same time and would not reveal whether the sites act in concert in the same cell type or whether they affect the same gene but act in different cell types. Distinguishing between these cases is critical for understanding and modeling gene regulation. The second reason is that whole-worm data may lack the sensitivity to detect regulatory events that occur in cell types that make up small fractions of the whole worm. scRNA-seq data (Cao et al. 2017; Packer et al. 2019) reveal important differences in gene expression that distinguish even individual cells. Such differences are presumably driven in part by regulatory regions that are only accessible in those cells; in a whole-worm assay, the signal from these highly cell type–specific regions could be drowned out by the noise generated from more populous cell types. Third, the lack of cell type resolution on these regulatory DNA maps confounds our ability to draw conclusions about differential activity across development. During development, the number of cells, and with it the diversity and proportion of cell types, is constantly changing. Thus, if an accessible site is less prominent in a later larval stage compared with an embryonic stage, this change could mean the site is more accessible in embryogenesis than in later development, or it could reflect that the site is more specialized in later stages and is accessible in a smaller fraction of the cells. Given these important limitations in the available data on *C. elegans* gene regulation, we sought to generate cell type–resolved chromatin accessibility maps.

Over the past few years, the technology to collect chromatin accessibility profiles of single cells has improved greatly. This technology relies on the assay for transposase-accessible chromatin followed by high-throughput sequencing (ATAC-seq) (Buenrostro et al. 2013), which treats permeabilized nuclei with a hyperactive Tn5 transposome from prokaryotes (Adey et al. 2010) to simultaneously cut accessible sites in the genome and ligate sequencing adapters onto the fragment ends on either side of the cut site (a reaction referred to as “tagmentation”). The resulting library is then amplified and sequenced. The simplicity of the assay significantly reduces the requirements for input material compared with DNase-seq (Song and Crawford 2010), and protocols have adapted ATAC-seq to work on single cells (Buenrostro et al. 2015; Cusanovich et al. 2015; Chen et al. 2018). These and other studies have shown in multiple systems that single-cell ATAC-seq (scATAC-seq) can measure thousands of sites per cell type and can identify distinct cell populations with high sensitivity. The use of the single-cell combinatorial indexing variant of single-cell ATAC-seq (sci-ATAC-seq) permits the cost-effective capture of scATAC-seq data. This assay probabilistically identifies DNA fragments isolated from single cells by first sorting 2500 nuclei per well into a 96-well plate, treating the nuclei in each well with a Tn5 enzyme loaded with adapters that contain a unique barcode sequence, and then pooling and re-sorting 25 nuclei per well into new 96-well plates in which a second set of barcodes is incorporated by using well-specific primers during library amplification. After sequencing, the reads can be assigned to a particular cell based on their combination of Tn5 and PCR barcodes. The sci-ATAC-seq assay has been successfully leveraged in several previous studies, including the identification of differences in gene regulation across germ layers in *Drosophila* embryogenesis (Cusanovich et al. 2018b), the generation of an atlas of 85 different clusters of cells from 13 different mouse tissues (Cusanovich et al. 2018a), and the identification of cell types in hippocampal tissue from mice (Sinnamon et al. 2019).

Here we sought to obtain a comprehensive cell type–resolved map of the regulatory DNA in a whole metazoan, the nematode *C. elegans*. We collected single-cell chromatin accessibility data from tens of thousands of nuclei (hereafter referred to as cells for simplicity) isolated from L2 animals to match previously published scRNA-seq data (Cao et al. 2017). To contend with the sparsity of single-cell chromatin accessibility data and to reduce dimensionality, we implemented an improved latent Dirichlet allocation (LDA) model (Blei et al. 2003; Griffiths and Steyvers 2004) that can scale to tens of thousands of cells by parallelizing the training process across multiple cores, a feature that was unavailable in a previously published LDA implementation for single-cell analysis (González-Blas et al. 2019). By training LDA models on our scATAC-seq data and analyzing the results in conjunction with the L2 sci-RNA-seq expression data (Cao et al. 2017), we were able to identify the tissue, and even cell type origins, of the cells. We compared our maps of chromatin accessibility with those from prior studies of accessible chromatin in whole animals, both to validate our results and to determine which sites were novel to our data. Finally, we assessed whether genes expressed broadly across tissues might nevertheless show multiple, more cell type–specific regulatory patterns, for example, by having tissue-specific accessible sites at alternative 5′ ends. We anticipate that these data will provide a valuable resource for studying regulatory biology in the worm and set the stage for future scATAC-seq experiments on additional life stages. In conjunction with cell type–specific gene expression data, these maps of candidate regulatory regions will help reveal the gene regulatory networks driving development in *C. elegans*.

## Results

### Single-cell chromatin accessibility in *C. elegans* with sci-ATAC-seq

To match the sci-RNA-seq data, we grew a synchronized population of wild-type worms to the middle of L2. At this stage, about 700 of the 959 somatic cells in the adult hermaphrodite have been produced, and the vast majority are terminally differentiated, but the development of the gonad has not progressed far enough to begin producing the thousands of germline nuclei that at later developmental stages would severely bias our collection of tissue types. After harvesting the worms, we fixed and isolated the nuclei, froze them in aliquots, and used these wild-type nuclei as input to the sci-ATAC-seq (Cusanovich et al. 2015, 2018a,b). We collected sci-ATAC-seq data for 30,930 cells with at least 150 unique reads per cell (median, 672 reads per cell), which represents ∼40× sampling of each cell in the L2 worm (Supplemental Fig. S1). Note that we expect thousands of genes to be expressed per cell, and thus, at a median of 672 reads per cell, we are only sampling a small fraction of the accessible regulatory sites in any given cell.

**Figure 1.**
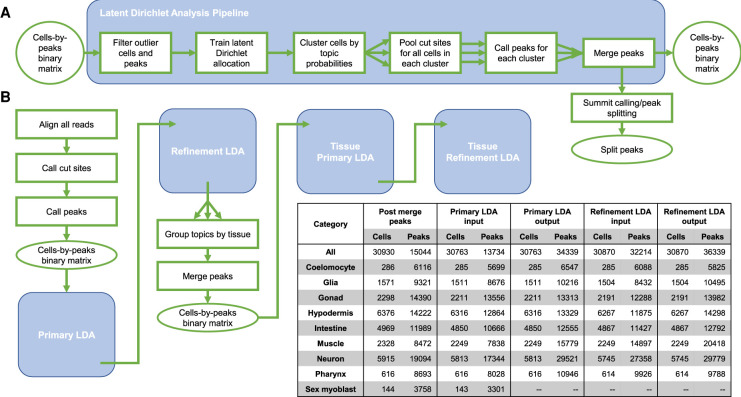
An iterative peak calling procedure yields more peaks from the complex mix of worm cell types. (*A*) The core peak calling procedure is to model the data using latent Dirichlet allocation (LDA), cluster the cells, and call peaks based on the clusters. (*B*) A flow chart represents the overall peak-calling strategy. First, bulk peaks are called, followed by two iterations of clustering and peak calling based on an LDA model. Then, we group the cells by tissue and repeat the two steps of clustering and peak calling. The number of cells included and the number of peaks called at each step are given in the *inset* table.

The postsequencing pipeline consists of aligning the paired-end reads to the WS235/ce11 version of the *C. elegans* genome and identifying cut sites as the 60-bp regions centered on the ends of the DNA fragments defined by the mapped mate pairs (for details, see Methods). Next, we identified which loci were accessible in each of our cells. There exists no unbiased annotation of cell type–resolved regulatory regions in *C. elegans*, so we called peaks directly from the sci-ATAC-seq data in an iterative fashion (Fig. 1A). The first step was to call peaks with MACS2 (Zhang et al. 2008) using all of the reads together, as with a bulk ATAC-seq data set. To detect additional cell type–specific peaks that could be obscured by the background reads coming from the most abundant cell types in this complex cell mixture, we clustered the cells based on the output of the LDA modeling technique (Blei et al. 2003) using the bulk peaks, pooled the cut sites from the cells in each cluster, and called peaks for each cluster using MACS2 (Fig. 1B, primary LDA). Finally, in order to refine our peak set, we repeated the clustering, pooling, and peak calling once more (Fig. 1B, refinement LDA).

Each peak-calling iteration centers around modeling the peak distribution in individual cells, as represented by a cells-by-peaks matrix containing binary values indicating whether or not a cell reports a read overlapping a particular peak. This binary matrix is input into LDA, a Bayesian modeling approach that was originally developed in the field of semantic analysis of text documents and has recently been used to model single-cell genomics data (Dey et al. 2017; González-Blas et al. 2019; Kim et al. 2020). We discuss this modeling approach in detail below and in the Methods. After our iterative clustering and peak calling procedure, we postprocessed the merged peaks from the refinement LDA step by detecting local maxima (summits) in the signal over each peak and splitting any peaks with multiple summits into separate contiguous segments. This step splits wide peaks to better capture accessible regions that may contain multiple binding sites. We share the pipeline output in a UCSC track hub, which can be accessed at the following URL: http://genome.ucsc.edu/cgi-bin/hgTracks?db=ce11&hubClear=http://waterston.gs.washington.edu/atacTissue/Durham_hub.txt.

### Single-cell peaks are concordant with regulatory regions from published bulk chromatin assays

**Figure 2.**
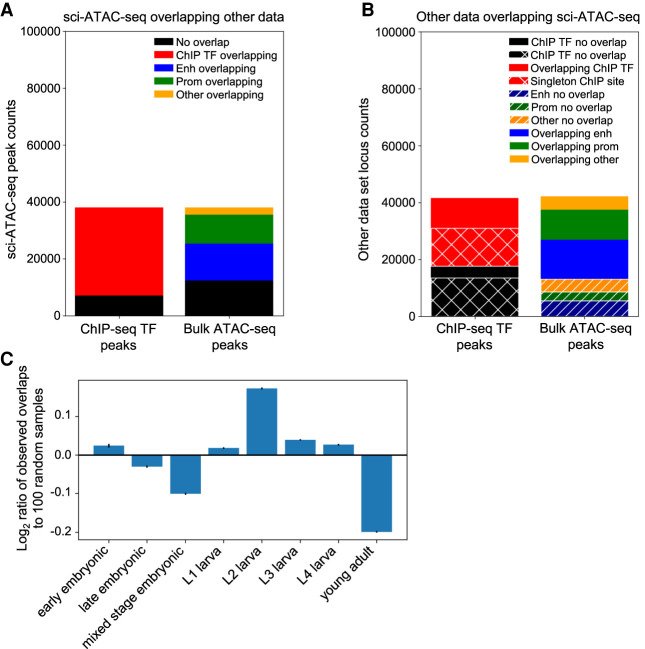
The peaks called from sci-ATAC-seq data show substantial overlap with existing chromatin data collected from whole worms. (*A*) The majority of sci-ATAC-seq peaks overlap sites called in the transcription factor (TF) ChIP-seq peaks from modERN or the bulk ATAC-seq peaks from Jänes et al. (2018). (*B*) Peaks from the other data sets also show substantial overlap with sci-ATAC-seq peaks. Most of the ChIP-seq TF peaks that do not overlap a sci-ATAC-seq peak are singleton peaks that are only found in a single experiment. (*C*) Breaking out the ChIP-seq peak overlaps by the developmental stage of the worms assayed and comparing the distribution across stages of the peaks with overlaps compared with the stage distribution for randomly selected ChIP-seq peaks show an enrichment for peaks found in larval stage L2. Error bars, 95% confidence interval.

After applying our iterative peak calling procedure, the whole-worm refinement LDA (Fig. 1) identified 36,339 peak regions, and splitting the multisummit peaks resulted in a total of 38,017 peaks. To compare our peak calls to other maps of regulatory DNA in *C. elegans*, we intersected our regions with peaks from two other data sets: bulk, whole-worm ATAC-seq data from samples spanning the *C. elegans* life cycle (Jänes et al. 2018), and TF binding site peaks identified from 427 whole-worm TF ChIP-seq data sets from the modERN Consortium (Fig. 2; Kudron et al. 2018).

We find good overlap with both published data sets but also some differences (Fig. 2A). First, intersecting the sci-ATAC-seq peaks with the bulk ATAC-seq data set shows 25,675 peaks of 38,017 (∼66%) overlapping a bulk ATAC-seq site overall. This fraction is significantly greater than expected by chance (Fisher's exact test *P* = 0), because the sci-ATAC-seq peaks cover 20,234,260 bp in total (∼20.2% of the genome), and the bulk ATAC-seq peaks cover 6,376,655 bp (∼6.4% of the genome). About 50% (12,960) of the overlaps were with bulk ATAC-seq sites classified as enhancers (Jänes et al. 2018), ∼40% (10,219) were with sites classified as promoters, and 10% (2496) were with other kinds of sites (e.g., noncoding RNAs). We find more extensive overlap with TF ChIP-seq sites; 30,886 of the 38,017 sci-ATAC-seq peaks (∼81%) overlap TF ChIP-seq peaks from modERN (Fig. 2A). The ChIP TF peaks cover 23,225,218 bp (∼23.2% of the genome), and similarly to the bulk ATAC-seq overlaps, the number of TF ChIP-seq peak overlaps is highly significant (Fisher's exact test *P* = 0).

Looking at the data from the opposite perspective (Fig. 2B), almost three-quarters of bulk ATAC-seq sites overlap a sci-ATAC-seq peak (29,021 of 42,102, ∼69%), and these overlaps are fairly evenly split between sites classified as promoters (10,684 of 13,833, ∼77%) and enhancers (13,715 of 19,195, ∼71%), with the remaining overlaps (4622 of 9,074, ∼51%) involving other categories of regulatory elements. In the case of the modERN TF sites, we find that the majority overlap a sci-ATAC-seq peak (23,863 of 41,542, ∼57%). Most of the ChIP-seq sites that do not overlap a sci-ATAC-seq peak are “singletons” that were only observed in one of the 427 ChIP-seq data sets (13,579 of 17,679, ∼77%). Because both the bulk ATAC-seq data set and the TF ChIP-seq data set contain data derived from across *C. elegans* development, we reasoned that many of the sites with no overlap in our sci-ATAC-seq peaks are specific to other developmental stages than L2. In support of this hypothesis, we find that the set of ChIP-seq sites that overlap sci-ATAC-seq peaks is enriched for those found in larval samples, especially L2, and depleted for sites observed in the embryo and young adult (Fig. 2C). We find similar results for the bulk ATAC-seq sites (Supplemental Fig. S2A), and the enrichment for TF ChIP-seq sites from L2 is even higher when only considering the singleton ChIP-seq sites (Supplemental Fig. S2B). Of the 18,543 singleton TF ChIP-seq sites that do not overlap a sci-ATAC-seq peak, only 929 (∼5%) are from data sets collected in L2.

We also checked the ChIP-seq signal over our sci-ATAC-seq peaks for four histone modifications (Jänes et al. 2018). The sci-ATAC-seq peaks are enriched for signal from the two activating histone marks H3K4me3 and H3K4me1, uncorrelated with signal from the H3K36me3 histone mark associated with actively transcribed gene bodies, and depleted for signal from the repressive histone mark H3K27me3 (Supplemental Fig. S3).

### LDA modeling reveals 37 clusters of cells

**Figure 3.**
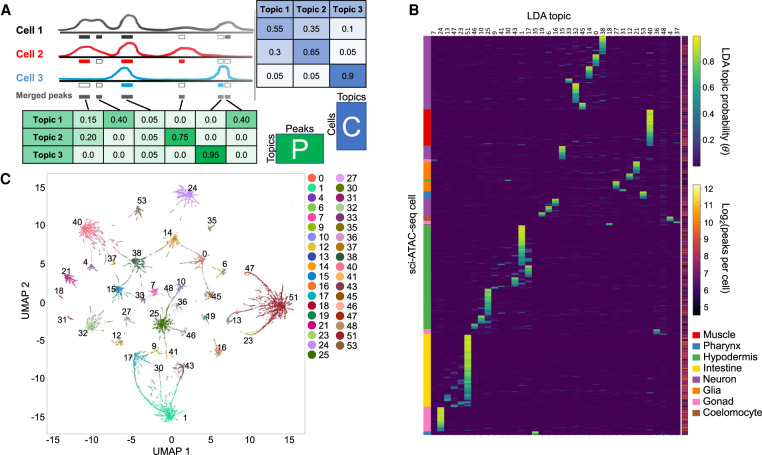
LDA modeling yields 37 major cell clusters that are characterized mostly by a single topic each. (*A*) LDA modeling learns latent topics that explain the data and return two matrices, here designated *P* and *C*. Matrix *P*, referred to in the text as the peaks-by-topics matrix, captures the probability distribution of each topic over all peaks, whereas matrix *C*, referred to in the text as the cells-by-topics matrix, captures the probability distribution of each cell over all topics. (*B*) Heatmap showing the normalized *C* matrix values for the 37 topics associated with clusters; this plot highlights that most cells have probability concentrated in one or a few topics. Cell types determined for the topics based on analysis of the *P* matrix are annotated on the *left*, and the number of peaks per cell is shown to the *right*. (*C*) UMAP embedding of the *C* matrix colored to indicate the 37 cell clusters. Any cells that are not assigned to a cluster are plotted as small gray dots and are mostly found on the periphery of the clusters.

To interpret our data at the level of tissues and cell types, we applied LDA (Blei et al. 2003), a statistical modeling technique that is particularly well suited to finding patterns in the sparse data generated by single-cell genomics assays (Fig. 3A). LDA has been applied previously to analyze single-cell chromatin accessibility (González-Blas et al. 2019), single-cell chromatin conformation (Kim et al. 2020), and single-cell gene expression data (Dey et al. 2017). LDA is a generative Bayesian modeling approach that was developed in the context of document classification. In the document classification task, the model is trained to identify information-rich words in a document corpus and to associate those words with latent topics that can distinguish the documents. The output consists of two matrices: one that captures the probability distribution of each topic over all words, and another that captures the probability distribution of each document over all topics. Thus, each topic is defined as some combination of words, and each document is modeled with some combination of topics based on its word content.

When applied to scATAC-seq data, cells are treated as documents, and peaks are treated as words. The model learns the peaks associated with latent “regulatory topics” that capture patterns that discriminate among regulatory states and cell types. The output consists of two matrices: one representing the distribution of peaks over topics, and another representing the distribution of topics over cells. A key advantage of LDA in this setting is that it handles sparse data quite well and leverages information from all cells at once to assign peaks to topics and all peaks at once to assign topics to cells.

We trained an LDA model with 55 topics, choosing 55 topics based on a fivefold cross-validation hyperparameter search procedure (see Methods) (Supplemental Fig. S4). This model yielded a cells-by-topics matrix with 30,870 rows (one for each cell after filtering out cells with too few peaks detected) and 55 columns, as well as a peaks-by-topics matrix with 32,214 rows (one for each peak after filtering out outlier high- and low-coverage peaks) and 55 columns (Fig. 3A). (Note that in the text, we transpose the topics-by-peaks matrix and refer to it as the peaks-by-topics matrix for consistency with the cells-by-topics matrix.) We expected that differences in chromatin accessibility among cell types would be the largest source of covariation in the data and, thus, that there would be many topics that corresponded to distinct cell types.

To look for topics that might distinguish cell types, we first removed 15 topics that did not have a high probability in any subset of tightly grouped cells (Methods) (Supplemental Fig. S5). Next, we assigned cells with >50% probability for one of the 40 remaining topics to “topic clusters.” For any topic cluster with fewer than 150 cells, we assigned unassigned cells (i.e., that had <50% probability for all topics) from the periphery based on their proximity to the centroid of that topic cluster. We removed three topics that still had fewer than 50 cells because they would have too little coverage for peak calling. Thus, by using this procedure, we assigned a total of 24,503 cells to 37 topic clusters for further analysis. We report the cells-by-topics matrix in Figure 3B and the number of cells per topic in Table 1. We describe how we assigned cell types to these topic clusters in the ensuing sections.

**Table 1.**
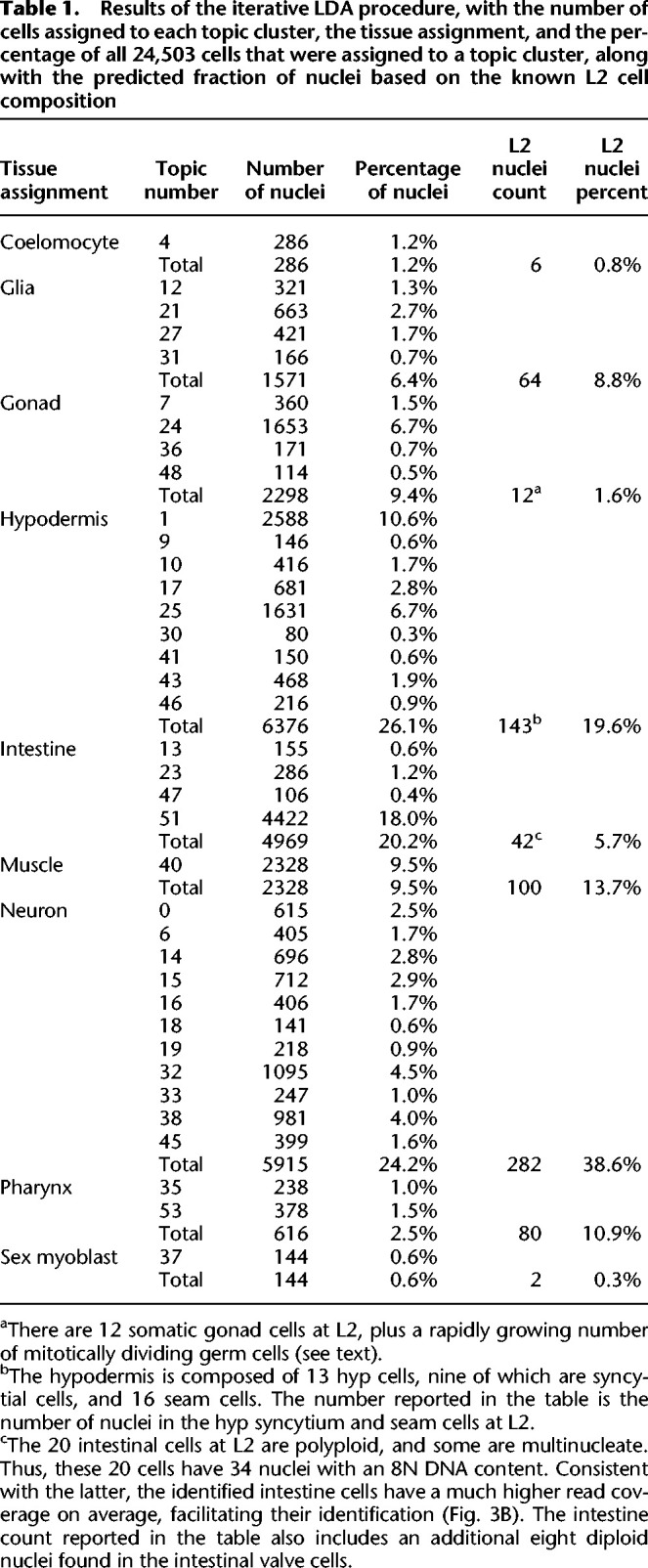
Results of the iterative LDA procedure, with the number of cells assigned to each topic cluster, the tissue assignment, and the percentage of all 24,503 cells that were assigned to a topic cluster, along with the predicted fraction of nuclei based on the known L2 cell composition

Visualizing these clusters with uniform manifold approximation and projection (UMAP) (McInnes et al. 2018; Becht et al. 2019) shows clear separation among groups of cells (Fig. 3C). We also note that, although we focus on the 37 topics that drive our cell clusters, the remaining topics could still contain useful information. Some topics with high probability in too few cells to be used for clustering may characterize cell types that are rare or that were less successfully isolated. In addition, topics with more diffuse probability across cells could correspond to other phenomena, for example, to different kinds of regulatory activity (e.g., promoters or enhancers) (González-Blas et al. 2019), cells with more complex patterns of regulatory activity, cells with noisy signal, or cell types with insufficient signal to be confidently clustered.

### Topics correspond to specific tissue identities

After clustering our cells based on 37 topics, we sought to determine whether these clusters of cells represent different cell types. As with other dimensionality reduction techniques (e.g., principal component analysis), LDA is an unsupervised algorithm with no restrictions on what qualities of the data it uses to determine the topics, and interpretation of the topics can be challenging.

**Figure 4.**
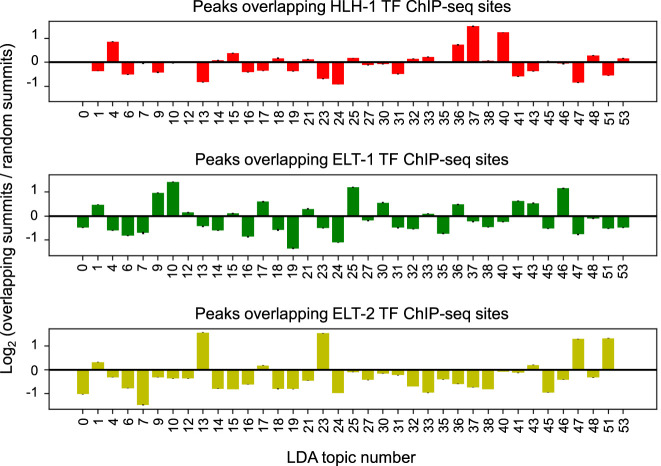
Overlapping peaks important for each topic with ChIP-seq peaks collected from cell type–specific TFs suggests at least some topics represent tissue types. Peaks associated with each topic were overlapped with ChIP-seq peaks for three cell type–specific TFs: HLH-1, which is specific for muscle (*top*); ELT-1, which is specific for seam cells (*middle*); and ELT-2, which is specific for the intestine (*bottom*). Topic distributions for peaks with ChIP-seq site overlaps were compared with the topic distribution for randomly sampled peaks, and the results are plotted here as the log_2_ ratio of the overlap topic distribution to the random topic distribution. Error bars, 95% confidence interval for 100 random samples.

One way to assess whether the topics show some tissue specificity is by cross-referencing the sci-ATAC-seq peaks with what is known about those loci in the literature, similarly to how marker genes are identified for clusters in scRNA-seq data (Cao et al. 2017; Packer et al. 2019). In the absence of broad data sets for cell-specific regulatory elements, we began by looking for overlap of the ATAC-seq peaks for each topic with the ChIP-seq peaks from cell type–specific TFs. For each of the 37 topics that we used to cluster the cells, we found all peaks in the peaks-by-topics matrix with probability greater than zero for that topic and overlapped them with all available ChIP-seq peaks from sites found in 40 or fewer other ChIP-seq data sets (i.e., non-high-occupancy-target [non-HOT] sites) (Kudron et al. 2018) for three TFs with known cell type–specific expression patterns: HLH-1, a master regulator for body wall muscle (Krause et al. 1990); ELT-1, a master regulator for hypodermis in embryos and seam cells in L2 larvae (Page et al. 1997); and ELT-2, a TF important in intestine development (Fukushige et al. 1998). We compared the number of overlaps in each topic to the number we would expect if the overlaps were random (i.e., if topics were not cell type–specific), and we expressed this comparison as a log_2_ ratio between observed and random overlap counts (Fig. 4). We find topics with specific enrichment for overlaps with peaks from each TF (95% confidence intervals are provided in Fig. 4). Peaks that characterize topics 37 and 40 are most enriched for overlaps with HLH-1 sites; peaks for topics 9, 10, 25, and 46 are particularly enriched for overlaps with ELT-1 sites; and peaks for topics 13, 23, 47, and 51 are most enriched for overlaps with ELT-2 sites. This analysis suggests that at least some of the topics are representing different tissues and that the cells enriched in topics characterized by many peaks overlapping HLH-1, ELT-1, and ELT-2 sites are likely muscle, hypodermis, and intestine cells, respectively. We show the same overlap analysis for all 283 TFs in Supplemental Figure S6.

Encouraged by the analysis of overlaps with ChIP-seq data from cell type–specific TFs, we sought to leverage the L2 sci-RNA-seq data (Cao et al. 2017) to do a more comprehensive analysis of all 37 topics. To do this, we mapped the sci-ATAC-seq peaks to the nearest downstream exon within 1200 bp, thereby accounting for peaks at alternative promoters and within introns. Assigning regulatory regions to their nearest gene is a simple and commonly used heuristic that works well in *C. elegans* (Araya et al. 2014). In contrast to mammals and other more complex animals (Pliner et al. 2018), there is little evidence that regulation by distal sites plays a prominent role in *C. elegans* (Reinke et al. 2013). Furthermore, the *C. elegans* genome is compact and gene-dense, meaning that most regulatory sites are found close to genes, either in intergenic sequences or in introns (Reinke et al. 2013). In total, we were able to assign 19,532 peaks of our 36,339 total peak calls to 17,389 genes. The number of genes we associate with accessible regions is higher than the number of genes known to be expressed in L2 worms (Boeck et al. 2016; Cao et al. 2017). There are several technical reasons for this, as well as potential biological reasons, including sites that play repressive roles, persistent sites from earlier developmental stages, or sites that are poised for later expression.

**Figure 5.**
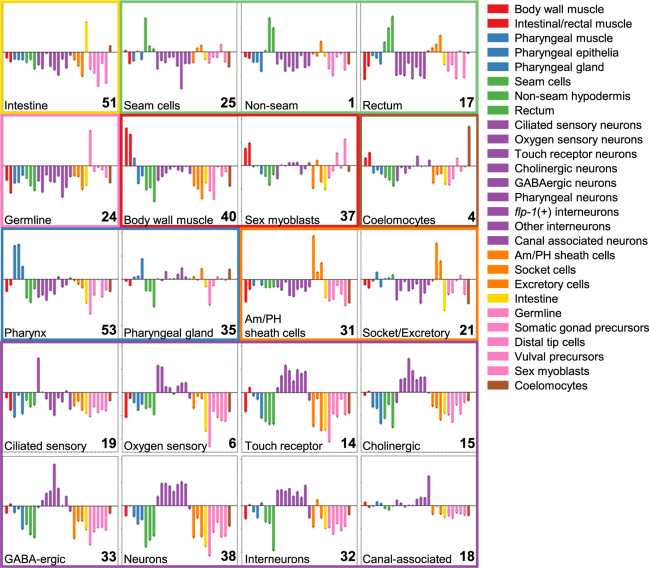
Topic-specific peaks tend to be near tissue-specific genes. Peaks associated with each topic were mapped to the nearest downstream gene, and the tissue expression distribution of the genes near the top 250 most-specific peaks for each topic was compared with the tissue expression distribution of 250 randomly selected genes. Here we plot the results as the log_2_ ratio of the topic-associated tissue expression distribution to that of randomly selected genes. Error bars, 95% confidence interval after comparing to the tissue expression distribution of 100 random samples. LDA topic numbers are shown in the *bottom right* corner of each plot. Topics with similar tissue-specificity patterns are grouped together, and the tissue type names and colors are as in Cao et al. (2017). Tissue assignments were made by eye based on the tissue with maximal fold-change and are written in the *bottom left* corner of each plot. If no single tissue was clearly the maximum, then a more general tissue annotation was chosen (e.g., “neurons” for topic 38). These annotations may need to be revisited with new data. Note that for concise visualization in this figure, we display just 20 of our 37 topics, but we report a version of this figure with all 37 topics in Supplemental Figure S9. All plots have the same *y*-axis range, from a log_2_ ratio of −4.5 to 4.0.

With these caveats, we used these peak–gene assignments with their associated expression patterns to associate genes with topics and thereby infer whether or not the topics are clustering cells by tissue type. For each topic, we identified the top 250 most topic-specific peaks (Supplemental Fig. S7) along with the set of genes that were associated with these peaks from our analysis above. Based on the normalized sci-RNA-seq expression values for each gene for 27 tissue types (http://genome.sfu.ca/gexplore/gexplore_search_tissues.html), we derived a tissue expression distribution for each gene. For each topic, we computed the mean tissue expression distribution for the genes associated with the top 250 most topic-specific peaks and then calculated the log_2_ ratio of that to the mean tissue expression distribution of 250 randomly selected genes (for an example schematic, see Supplemental Fig. S8). Our results show that these topic-specific peaks are near genes with tissue-specific expression patterns and suggest that these topics reflect specific cell types (Fig. 5; Supplemental Fig. S9). In fact, the genes associated with many of the topics show evidence of enrichment for tissue subtypes, including distinct kinds of neurons and hypodermis, and even some small but clearly distinct cell populations, such as sex myoblasts. At this resolution, there appears to be no distinction between body wall muscle and intestinal/rectal muscle (topic 40 encompasses both), and it is not possible in all cases to assign a subtype to topics enriched in neuronal genes (e.g., in topic 38).

Overall, we find reasonable agreement between the number of nuclei we observe in our tissue clusters and the number of nuclei we expect based on the worm anatomy (Table 1; Sulston and Horvitz 1977; Altun and Hall 2002, 2009). During L2, the number of nuclei in each worm increases from 674 to more than 731, and our synchronized worm culture likely sampled worms over several hours of development. One of the most dynamic tissues in this time is the gonad, as the germline begins mitotic divisions in early L2. Indeed, topic 24 (enriched for germline) adds nearly triple the number of nuclei to the gonad total as the other (somatic) gonad topics combined.

**Figure 6.**
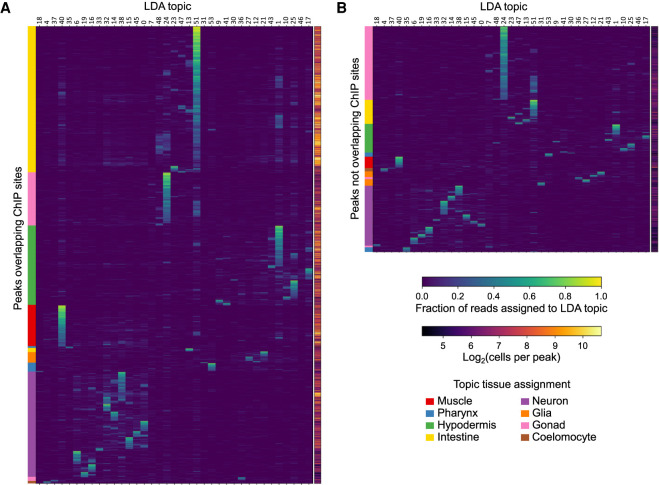
Novel sites of accessible chromatin with no overlapping modERN ChIP-seq peaks show topic specificity. We compare the normalized peak-by-topic matrix values between the peaks that overlap a ChIP-seq site (*A*) and those that do not (*B*). The nonoverlapping peaks are enriched for topics associated with gonad (especially germline/topic 24) and topics associated with neurons. The nonoverlapping peaks also tend to be observed in fewer cells.

After identifying cell types associated with our topics, we revisited the list of peaks that had no overlap with TF ChIP-seq sites and looked for differences between those with overlaps and those without (Fig. 6). In general, most peaks contribute to primarily one topic with appreciable contributions to a few additional topics. The peaks that overlap TF ChIP-seq sites (Fig. 6A) tend to be found in many more cells in the L2 animal than the peaks without TF ChIP-seq overlaps (Fig. 6B). Nevertheless, the peaks without ChIP-seq overlaps still show clear topic specificity, suggesting that they are informative peaks. In particular, over half of the peaks with no ChIP-seq overlaps contribute to neuron or gonad topics. Neurons are the most diverse tissue type in the worm, with scRNA-seq capable of identifying transcriptional signatures consistent with specific neuron cells (Packer et al. 2019); their absence in the ChIP-seq data suggests that whole-worm ChIP-seq lacks the sensitivity to find the cell type–specific regulatory sites present in only a few L2 cells. On the other hand, TFs for gonad tissues are not well characterized by the current modERN ChIP-seq compendium. In support of the increased sensitivity of sci-ATAC-seq over bulk whole-worm assays, we find that the top 250 most-specific peaks for each topic that we used to infer the cell types of our topic clusters (Fig. 5; Supplemental Fig. S7) are depleted for overlaps with TF ChIP-seq sites and bulk ATAC-seq sites (Supplemental Fig. S10). Thus, we conclude that the sci-ATAC-seq peaks that do not overlap TF ChIP-seq sites most likely either are highly cell type–specific or are specific to TFs that have not yet been tested with ChIP-seq.

**Figure 7.**
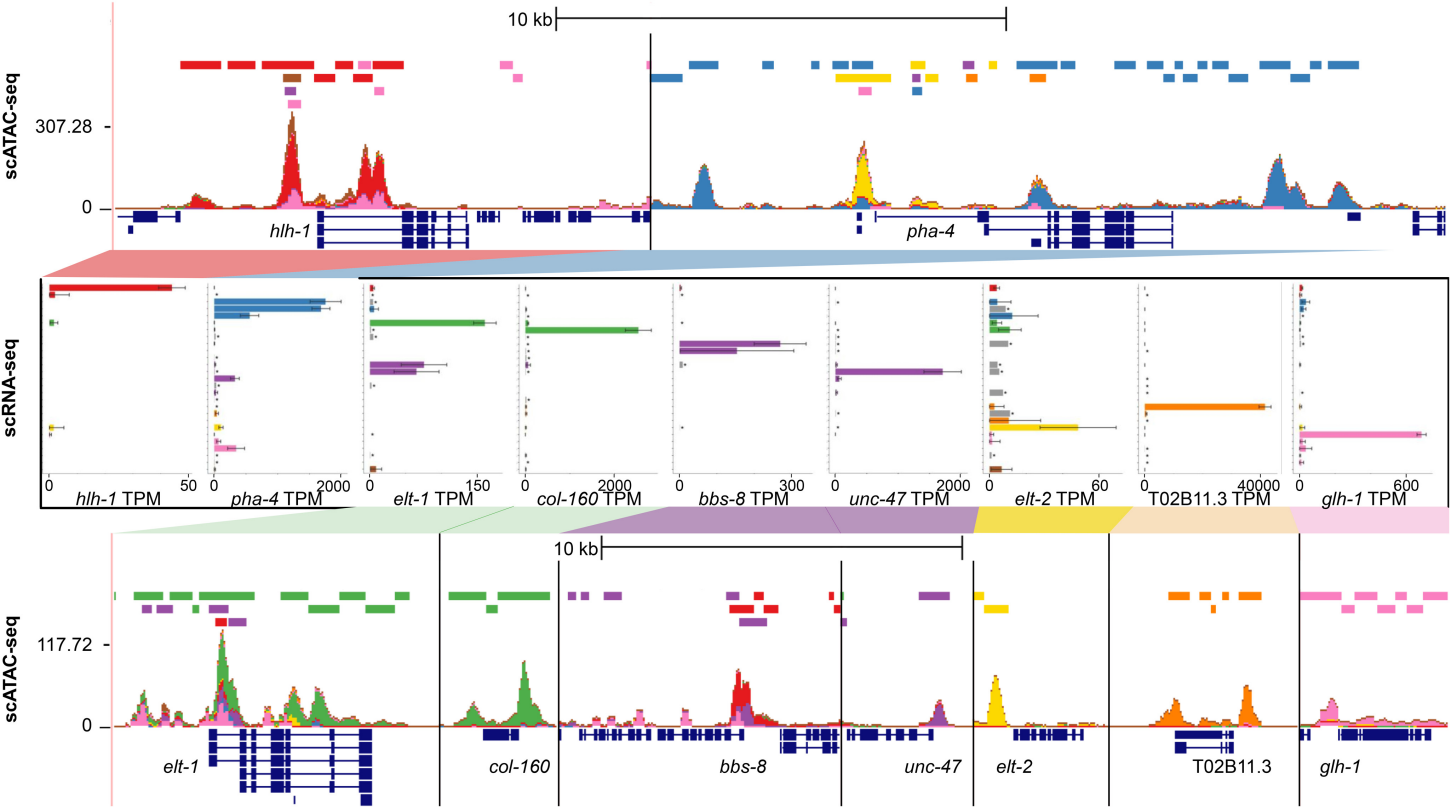
Known tissue-specific genes show topic-specific chromatin accessibility. UCSC Genome Browser multilocus view of the regions surrounding nine known tissue-specific genes (*top* and *bottom*), as well as the tissue expression patterns from sci-RNA-seq (*middle*). In each genome browser view, the *top* track shows the locations of sci-ATAC-seq peaks colored by tissue type, the second track shows the stacked sci-ATAC-seq signal from each tissue, the third track shows consensus peak regions around local maxima in the signal track, and the fourth track shows the gene models. The gene expression bar plots show expression values for 27 tissues in TPM units, with the same coloring and order as the legend in Figure 5.

We also investigated the sci-ATAC-seq signal at known tissue-specific genes, and we find strong tissue-specific chromatin accessibility that is consistent with the known expression patterns of these genes (Fig. 7). The genes *hlh-1*, *pha-4* (a master regulator of pharyngeal tissue), *elt-1*, *col-160* (a collagen gene that is expressed in nonseam hypodermis in L2), *bbs-8* (a gene encoding a receptor expressed in ciliated sensory and oxygen sensory neurons), *unc-47* (a gene expressed in GABA-ergic neurons), *elt-2*, T02B11.3 (a gene that is specifically expressed in sheath glial cells), and *glh-1* (a gene expressed specifically in the germline) all show accessibility enriched in the expected tissue types. The data also suggest patterns of differential isoform expression; for example, of the three *pha-4* isoforms, the 5′ end of the long isoform is most accessible in intestine cells, whereas the 5′ end of a medium isoform has almost no accessibility, and the shortest isoform has pharyngeal accessibility. There are also several sites downstream from the *pha-4* gene that are strongly accessible in the pharynx and perhaps represent other sites that play a role in regulation of this locus.

Given the isoform-specific accessibility pattern over the *pha-4* locus, we asked whether this is a more general phenomenon and searched for other genes that show similar patterns. Many genes, both with and without multiple 5′ ends, are broadly expressed and show complicated patterns of chromatin accessibility in the sci-ATAC-seq data that suggest the presence of tissue-specific regulatory sites but that can be difficult to interpret (Supplemental Fig. S11; Supplemental Table 1). To maximize interpretability, we searched for genes with multiple 5′ ends separated by at least 150 bp that are predominantly expressed in just two tissue types. We found dozens of examples of genes with compelling patterns of tissue-specific chromatin accessibility that suggest tissue-specific isoform usage (Supplemental Note 1). We compared the sci-ATAC-seq signal at these loci with bulk whole-transcript RNA-seq data from FACS-isolated embryonic tissues (Warner et al. 2019) and, despite the differences in stages assayed, find that in many cases the tissue-specific isoform patterns in the sci-ATAC-seq data are supported by the RNA-seq data (Supplemental Fig. S12).

### LDA modeling of cells from individual tissue types detects fine-grained cell types

Although the topics we identified can distinguish cells at the level of tissue type, to yield more specific cell identities, we tried a more focused analysis of cells from a particular cluster/tissue. We reran our LDA-based clustering procedure (Fig. 1; Supplemental Fig. S13) for cells of each tissue type to identify subclusters that correspond to more fine-grained cell types. We grouped the topics into eight major tissue types (coelomocyte, glia, gonad, hypodermis, intestine, muscle, neuron, and pharynx) and iteratively trained LDA models for each tissue, clustered the cells, and called peaks for each cluster. Similarly to the whole-worm analysis, we provide the results as a UCSC Genome Browser track hub, viewable at http://genome.ucsc.edu/cgi-bin/hgTracks?db=ce11&hubClear=http://waterston.gs.washington.edu/atacCellType/Durham_hub.txt.

**Figure 8.**
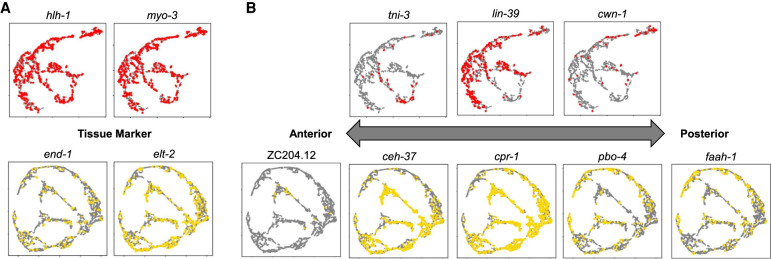
Subclustering of muscle and intestinal cells separates them by position along the anterior–posterior body axis. (*A*) Peaks near genes that should be expressed throughout a tissue, like *hlh-1* and *myo-3* in body wall muscle or *end-1* and *elt-2* in the intestine, show accessibility in cells throughout the UMAP. (*B*) In both the muscle and intestine data, we can detect subclusters of cells that show peaks near genes that mark the anterior or posterior regions of these tissues based on literature and microscopy data (Packer et al. 2019).

Body wall muscle cells are quite similar to each other, despite differentiating from four different embryonic lineages. In previous scRNA-seq studies, the body wall muscle cells all clustered together, without much separation (Cao et al. 2017; Packer et al. 2019). Nevertheless, within the body wall muscle cluster, the cells were found to group by anatomical position, setting up an anterior–posterior axis through the cluster that was identified by looking for the expression of specific marker genes. Similarly, the intestine cells showed evidence of expression differences in sci-RNA-seq by anatomical position. We looked for a similar anatomical pattern in the sci-ATAC-seq muscle and intestinal cell clusters (Fig. 8; Supplemental Figs. S14, S15). We found cells with peaks associated with marker genes that are expressed throughout muscle (*hlh-1* and *myo-3*) and intestine (*end-1* and *elt-2*) distributed throughout the clusters (Fig. 8A), whereas those cells with peaks associated with marker genes with anatomically biased expression showed distinct patterns (Fig. 8B). The subclustering thus appears to reveal still finer distinctions between cells.

**Figure 9.**
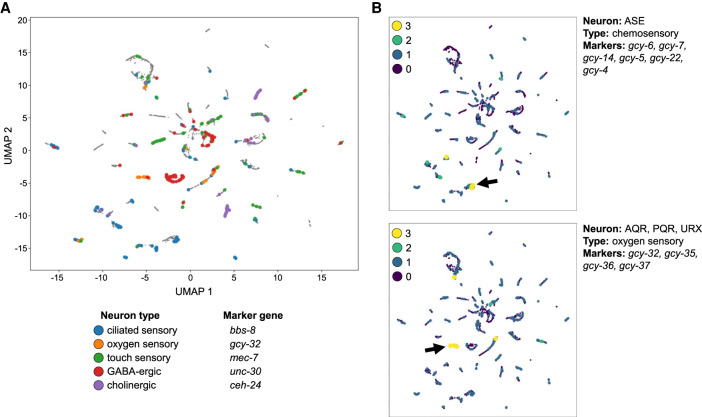
Subclustering of neurons reveals finer structure that distinguishes different types of neurons. (*A*) Cells with reads in peaks near genes with expression patterns specific to neuron subtypes cluster together (*bbs-8*: ciliated sensory neurons; *gcy-32*: oxygen sensory neurons; *unc-30*: GABA-ergic neurons; *mec-7*: touch receptor neurons; *ceh-24*: cholinergic neurons). (*B*) Cells in the UMAP plot are colored by the number of marker genes with nearby coaccessible peaks. Here, we show marker genes for the ASE neurons, a specific pair of ciliated sensory neurons, which are identified in one of the *bbs-8* clusters from *A* (marked by the *left*-facing arrow), and show marker genes shared by the oxygen sensory neurons AQR, PQR, and URX, which further support the cluster marked with *gcy-32* in *A* (marked by the *right*-facing arrow).

Next, we performed a similar analysis on all cells from neuron-enriched clusters. Although the tissue type categories based on the L2 sci-RNA-seq data (Cao et al. 2017) already break gene expression distributions down into nine different neuronal subtypes, there are many more specific neuron types revealed by anatomical analysis and by additional scRNA-seq data sets (Fig. 5; Packer et al. 2019; Taylor et al. 2019). To take a closer look at the neuron cells, we gathered all cells from topic clusters showing neuronal enrichment and performed the same analysis that we did for body wall muscle and the intestine above. The neuron LDA model yielded 36 topic clusters—only one fewer than the number of clusters found by the whole-worm model, highlighting the diversity of neuronal cell types. We evaluated five marker genes that we chose for their tissue-specific expression patterns in the sci-RNA-seq data: *bbs-8* is expressed in ciliated sensory neurons; *gcy-32* is expressed in oxygen sensory neurons; *unc-30* is expressed in GABA-ergic neurons; *mec-7* is expressed in touch-sensitive neurons; and *ceh-24* is expressed in cholinergic neurons. We find that the cells with chromatin accessibility near these genes are associated with distinct, well-separated clusters in UMAP space (Fig. 9A), suggesting cell type identities for these clusters.

To verify the neuron subtypes identified by single marker genes and also attempt to identify additional subtypes for the other clusters of cells, we followed up by checking additional marker genes for specific neurons (Fig. 9B). The ASE neurons are a pair of ciliated chemosensory neurons that detect water-soluble attractants like potassium and sodium ions. They express *bbs-8*, along with a highly specific repertoire of guanylyl cyclase (*gcy*) genes that encode sensory receptors.

We found chromatin accessibility peaks near six such receptor genes specific to ASE neurons: *gcy-4*, *gcy-5*, *gcy-6*, *gcy-7*, *gcy-14*, and *gcy-22* (Etchberger et al. 2009). To identify the ASE neurons in the UMAP, we colored cells by the number of coaccessible peaks they have that are near the ASE-specific *gcy* genes, which identifies one of the *bbs-8* subclusters as likely to be ASE neurons. We performed a similar analysis to confirm the UMAP cluster containing the AQR, PQR, and URX oxygen-sensory neurons. Oxygen-sensory neurons express their own repertoire of *gcy* genes, including *gcy-32*, *gcy-35*, *gcy-36*, and *gcy-37* (Packer et al. 2019), and peaks near these genes are coaccessible primarily in the cells marked by *gcy-32* in Figure 9A. We identified additional examples of neuron subtypes, including mechanosensory neurons, two types of motor neurons, and interneurons (Supplemental Fig. S16). We show the neuron UMAP colored by LDA topic probabilities in Supplemental Figures S17 through S20. Finally, we also subclustered coelomocyte (Supplemental Figs. S21, S22), glia (Supplemental Figs. S23, S24), gonad (Supplemental Figs. S25, S26), hypodermis (Supplemental Figs. S27, S28), and pharynx (Supplemental Figs. S29, S30), finding evidence for additional specific cell types in each of them. Thus, sci-ATAC-seq can make fine-grained distinctions among cell types at a resolution that approaches scRNA-seq and thereby begin to associate specific regions of accessible chromatin with specific cell types.

## Discussion

We used the sci-ATAC-seq assay to assemble the first cell type–resolved map of regulatory elements in *C. elegans*. We found 38,017 peaks, which we used to assign 24,503 of our 30,930 cells to one of 37 different clusters (Fig. 3) that represent distinct, differentiated tissues in the L2 nematode (Fig. 5). Our map, derived from data collected in essentially a single experiment, recovers the majority of L2 regulatory sites detected by hundreds of individual ChIP-seq experiments (Fig. 2) and proposes an additional 7131 novel regulatory sites not found in the ChIP-seq compendium (Fig. 6). Accessibility at these sites can distinguish among nearly all the major cell types in the worm (Fig. 5; Supplemental Fig. S9) and many minor ones, including highly similar cell types, such as muscle or intestine cells at different anatomical positions (Fig. 8), and cell types that have only a few examples per worm, especially neuron subtypes (Fig. 9; Supplemental Fig. S16). For example, there are only two ASE neurons in each worm, and our analysis suggests that we can identify one or two clusters that might harbor these cells (Fig. 9B). For accessibility peaks that overlap TF ChIP-seq sites (Kudron et al. 2018), they suggest the tissue in which the TFs are active, and in turn, the ChIP sites suggest which TFs are active at the sci-ATAC-seq peak (Fig. 6; Supplemental Fig. S6). We found dozens of examples in which nonoverlapping alternative 5′ exons are likely regulated by distinct tissue-specific accessibility sites, supporting the notion that a principal reason for alternative 5′ ends is for tissue-specific regulation (Supplemental Note 1; Supplemental Fig. S12). In addition, there are many other broadly expressed genes, both with and without multiple isoforms, that also show tissue-specific accessibility patterns (Supplemental Fig. S11; Supplemental Table 1).

The cell type resolution of sci-ATAC-seq data approaches that of scRNA-seq (Cao et al. 2017; Packer et al. 2019) despite the high levels of sparsity and low dynamic range of the data in individual cells. scATAC-seq suffers generally from sparsity and a low dynamic range because there are at most two chances in a diploid cell to sample a given accessible locus, depending on whether or not both alleles are accessible. This contrasts to scRNA-seq, which has hundreds or even thousands of chances for measuring the mRNA of highly expressed genes. The sparsity of scATAC-seq was particularly high in our data set because the worm samples yielded sci-ATAC-seq libraries with many fewer unique fragments per cell than other organisms. In the first reported sci-ATAC-seq results, human and mouse cell lines yielded a median of 2503 fragments per cell (Cusanovich et al. 2015), and in more recent work on fly and mouse cells, the median yield was over 10,000 fragments per cell (Cusanovich et al. 2018a,b). In contrast, despite using the most up-to-date protocol (Cusanovich et al. 2018a,b), the median *C. elegans* cell yielded only about 700 fragments (Supplemental Fig. S1). We hypothesize that this could be improved by optimizing the nuclear isolation and permeabilization conditions: L2 nuclei are extremely small, compact, and dense (∼2 µm in diameter), and possibly after formaldehyde fixation, Tn5 has only limited access to the chromatin. As single-cell technology advances and we are able to generate more complex libraries, we expect that scATAC-seq will provide even higher resolution of individual cell types in the worm.

We also expect to see improved results from applying more advanced computational techniques. We provide a new LDA implementation that implements several useful features (see Methods), and we also look forward to new approaches that more tightly integrate the analysis of scATAC-seq and scRNA-seq data sets. There are multiple recent approaches for projecting single-cell data from different modalities into the same embedding space (Welch et al. 2017; Cusanovich et al. 2018a; Stuart et al. 2019). By jointly analyzing the sci-ATAC-seq data and sci-RNA-seq data with one of these methods, it may be possible to improve the cell type resolution of our chromatin accessibility maps.

Such accessibility maps with high cell type resolution will be important for understanding gene regulation on the scale of the whole genome across the whole organism. In addition, regulatory sites are hypothesized to play a major role in common disease and evolutionary adaptation, so maps of regulatory sites will aid in interpreting the effects of genetic variation. For example, many mutations that are linked to some phenotype by approaches like GWAS do not fall in genes. The implication is that, if one of the mutations is indeed causal, it must fall in a regulatory sequence of DNA. Thus, maps of cell type–specific regulatory regions can help interpret and prioritize candidate causal variants and will be a useful complement to genetic resources in *C. elegans*, including the *C. elegans* Natural Diversity Resource (Cook et al. 2017) and the Million Mutation Project strains (Thompson et al. 2013). Better annotations of regulatory DNA can also help with understanding comparative genomics and the evolution or conservation of stretches of noncoding DNA.

Given the importance of mapping regulatory sites for understanding genome structure and function, as well as the power of *C. elegans* as a model organism, improving and expanding our maps of regulatory regions should be a high priority. As single-cell genomics technology continues to improve, we will be able to attain better measurements across more cells and at lower cost. Coassays that can make chromatin accessibility and RNA-seq measurements in the same cells are showing very promising results, with the library preparation cost per cell recently dropping by orders of magnitude (Cao et al. 2018; Ma et al. 2020). By leveraging such approaches, collecting accessibility data for additional developmental stages in the worm will provide valuable insight into the dynamics of gene regulation over the course of development as cells differentiate. These data can be paired with new scRNA-seq data collected from throughout *C. elegans* embryogenesis (Packer et al. 2019), moving the field closer to having a truly comprehensive map of gene expression and regulation for every cell throughout development in *C. elegans*.

## Methods

### Nuclear isolation from whole L2 worms

We grew wild-type *C. elegans* worms (VC2010 strain) at 21°C on nine 150-mm plates and synchronized the population by bleaching (2% bleach, 0.5 M KOH) young adults with eight to 12 embryos to isolate embryos, hatching them at room temperature in egg buffer (118 mM NaCl, 48 mM KCl, 2 mM CaCl_2_, 2 mM MgCl_2_, HEPES 25 mM at pH 7.3) for 12–16 h, and replating the L1 hatchlings onto nine more 150-mm plates at a density of approximately 60,000 worms per plate. After two rounds of this bleach synchronization and plating, the L1 worms were allowed to grow for 19 h at 21°C after plating to reach the middle of L2. The worms were washed off eight of the plates with M9 buffer (22 mM KH_2_PO_4_, 22 mM Na_2_HPO_4_, 85 mM NaCl, 1 mM MgSO_4_ at pH 6.5) into a 50-mL conical tube. Bacteria were removed from the suspension by spinning the tube at ∼3000*g*, aspirating the supernatant, and resuspending in fresh M9. The M9 wash was repeated, and the supernatant was aspirated, leaving a worm pellet in ∼1 mL of M9. The worm pellet was flash-frozen by using a P1000 to transfer the worms drop by drop into a mortar containing liquid nitrogen. The frozen worms were crushed into powder with a pestle such that each worm broke into three to four chunks, and the powder was transferred to a 50-mL Falcon tube containing 8.75 mL of 1.1% formaldehyde in egg buffer supplemented with 1× protease inhibitor. Worms were rocked at room temperature for 10 min before the fixation reaction was quenched by adding 1.25 mL 1 M glycine (final concentration ∼125 mM) and incubated another 5 min at room temperature. The fixed worms were pelleted at 3220*g* for 5 min at 4°C, the supernatant was removed, and the pellet was resuspended in 10 mL ice-cold egg buffer. Fixed worms were pelleted again by spinning at 3220*g* for 5 min at 4°C. The egg buffer supernatant was aspirated, and the pellet was resuspended in ice-cold 2× nuclear preparation buffer (20 mM HEPES at pH 7.6, 20 mM KCl, 3 mM MgCl_2_, 2 mM EGTA, 0.5 M sucrose, 0.05% Triton X-100 in egg buffer) supplemented with protease inhibitor (NPB + PI). The following steps were all performed at 4°C or on ice: The solution was transferred to a 7-mL Dounce homogenizer, and the fixed worm chunks were homogenized with 20 loose pestle strokes followed by 10 tight pestle strokes. The Dounced suspension was spun for 90 sec at ∼200*g* in a swing-arm centrifuge to loosely pellet debris, and the top 1000 µL of supernatant (containing the nuclei) was removed to a 15-mL Falcon tube on ice. One milliliter of fresh NPB + PI was added to the Dounce, the debris pellet was gently resuspended, and the Douncing and spinning were repeated three more times, resulting in the collection of 4 mL of nuclei. The suspension of nuclei was cleaned by gently passing through a 10-µm syringe filter prewetted and chased with 1 mL ice-cold NPB + PI into a new 15-mL Falcon on ice. The nuclei were split evenly into 1.5-mL Eppendorf tubes and pelleted at 2000*g* for 10 min at 4°C. All supernatant was removed, and the pellets were each gently resuspended in 1 mL freezing solution (50 mM Tris at pH 8.0, 25% glycerol, 5 mM Mg(OAc)_2_, 0.1 mM EDTA, 5 mM DTT, 1× protease inhibitor cocktail [Roche], 1:2,500 SUPERase•In [Ambion]) (Cusanovich et al. 2018b). The resuspended nuclei were transferred to 2-mL cryotubes, flash-frozen in liquid nitrogen, and stored at −80°C.

### scATAC-seq via single-cell combinatorial indexing

The sci-ATAC-seq protocol was as described by Cusanovich et al. (2018b). Briefly, flash-frozen VC2010 nuclei were thawed in a 37°C water bath and put immediately on ice. The nuclei were transferred to a 1.5-mL Eppendorf tube and spun at 2000*g* for 10 min. The supernatant was aspirated, and the pellet was resuspended in 200 µL of ATAC-OMNI (Corces et al. 2017) RSB (10 mM Tris-HCl at pH 7.4, 10 mM NaCl, and 3 mM MgCl_2_ in water) supplemented with 0.01% digitonin, 0.1% IGEPAL-630, and 0.1% Tween 20; allowed to stand for 3 min on ice; and then quenched by adding 1 mL of RSB supplemented with 0.1% Tween 20. The resuspended and lysed nuclei were stained with 1× Hoechst, and a BD FACS Aria II was used to distribute 2500 nuclei into each well of a 96-well v-bottom plate (Eppendorf twin.tec LoBind skirted 96-well PCR plate) prepared with 19 µL of tagmentation reaction solution (10 µL 2× Nextera TD buffer, 3.3 µL 1× DPBS, 0.2 µL 1% digitonin, 0.2 µL 10% Tween 20, 5.3 µL H_2_O) (Corces et al. 2017). After sorting, 1.0 µL of 2.5 µM uniquely barcoded Tn5 from Illumina (Cusanovich et al. 2015) was pipetted into each well of the 96-well plate, and the transposition reaction was allowed to proceed for 30 min at 55°C. Next, 20 µL of STOP reaction buffer (40 mM EDTA and 1 mM Spermidine) was added to quench the reaction, and the plate was put for 15 min at 37°C. After stopping transposition, all nuclei were pooled into a 15-mL conical tube, restained with 1× Hoechst, and distributed by FACS into twenty-eight 96-well v-bottom plates at 25 nuclei per well. The 96-well plates contained 12 µL per well of reverse cross-linking buffer (0.83 mg/mL Proteinase K and 0.042% SDS in Qiagen EB buffer) and were put on ice, spun down, and frozen at −20°C in batches during sorting. Later, these plates were thawed in groups of four for reversing cross-links by incubating for 16 h at 65°C, after which the transposed and un-cross-linked fragments were amplified using uniquely barcoded PCR primers. We ran four wells as test reactions in qPCR and monitored the libraries for saturation of SYBR green signal to identify the number of cycles required for appropriate amplification (Cusanovich et al. 2018b), and then amplified the rest of the wells for either 22 cycles with Illumina NPM 2× PCR master mix or 23 cycles with NEBNext 2× PCR master mix. PCR reaction was 12.0 µL of nuclei in reverse cross-linking buffer, 2.5 µL of 5 µM Nextera v2 barcoded P7 PCR primer, 2.5 µL of 5 µM Nextera v2 barcoded P5 PCR primer, 1.0 µL of 100× BSA, 25.0 µL of 2× NEBNext PCR master mix (NEB M0541), and 7.0 µL nuclease free H_2_O. NPM PCR protocol was 3 min at 72°C; 30 sec at 98°C; repeat for 22 times 10 sec at 98°C, 30 sec at 63°C, 1 min at 72°C; and 4°C HOLD. NEBNext PCR protocol was 5 min at 72°C; 30 sec at 98°C; repeat 23 times 10 sec at 98°C, 30 sec at 63°C, 1 min at 72°C; and 4°C HOLD. After amplification, the fragments were cleaned up by pooling the contents of all wells and splitting across four Zymo Clean & Concentrate columns (D4014), eluted each in 25 µL Qiagen EB; combined the eluates; further cleaned and concentrated with 1× Ampure XP magnetic beads; and finally eluted in 25 µL. Library quality was assessed using the Agilent TapeStation D5000 kit (Screentape 5067-5588; reagents 5067-5589), and molarity was quantified for fragments between 200 and 1000 bp. Last, the libraries were combined into an equimolar pool at 2 nM for sequencing. Libraries were sequenced using the manufacturer's denaturation conditions and loaded either on a Illumina MiSeq 300 cycle v2 kit (MS-102-2002) at an input concentration of 15 pM, or on an Illumina NextSeq mid-output 300 cycle v2.5 kit (20024905) at an input concentration of 1.8 pM, using custom sequencing primers and recipe from Illumina.

### Generation of genomic DNA input control

To control for the sequence cutting bias of Tn5 (Green et al. 2012), we treated naked *C. elegans* genomic DNA with the bulk ATAC-seq protocol (Buenrostro et al. 2013) as follows. We isolated genomic DNA with phenol:chloroform extraction and ethanol precipitation. To keep the Tn5:DNA ratio similar to a bulk ATAC-seq experiment with 50,000 cells, we estimated that a typical *C. elegans* nucleus will contain 1 × 10^6^ bp × 2 genomes × 660 MW/bp × 1.67 × 10^−12^ pg/MW ≈ 0.22 pg/nucleus, or ∼11 ng in 50,000 nuclei. We diluted the DNA to a concentration of ∼0.87 ng/µL, as measured with the Qubit high-sensitivity assay (Invitrogen), and used 11.5 µL as input to a 25-µL reaction with 12.5 µL of Nextera TD buffer and 1.0 µL of Nextera Tn5 enzyme. The reaction was incubated for 30 min at 37°C, cleaned up with a Qiagen MinElute column, and amplified using NEBNext 2× PCR mix with primers from Buenrostro et al. (2013). The libraries were cleaned up with 1:1 AMPure XP magnetic beads and sequenced on the Illumina NextSeq platform.

### ATAC-seq alignment pipeline

Initial processing of the sequencing results was performed as reported by Cusanovich et al. (2018b), with some changes. Sequencing results were converted to FASTQ format with the Illumina bcl2fastq program (v. 2.19). First, the integrity of the barcode sequences was checked for each of the four components of the barcode (tagmentation barcodes from both sides of the cut and the P5 and P7 primer indices added during PCR amplification) by matching the sequencing results to the known barcode sequences. Any read that had three or fewer edits compared with the best-matching known barcode sequence and that had no other known barcode sequences matching with five or fewer edits was corrected and assigned to the best-matching barcode sequence. Any read-through of short templates was corrected by trimming adapter sequences from reads using Trimmomatic (v 0.36) (Bolger et al. 2014) with the options ILLUMINACLIP:NexteraPE-PE:2:30:10:1:true, TRAILING:3, SLIDINGWINDOW:4:10, and MINLEN:20. Next, read sequences were aligned to the WS235/ce11 build of the *C. elegans* genome with Bowtie 2 (Langmead and Salzberg 2012) with options –X 2000 and -3 1; properly paired reads with mapping scores greater than 10 were kept; any reads mapping to the mitochondrial DNA were filtered out; and read pairs with identical barcode sequence and with identical starting and ending mapping coordinates were identified as PCR duplicates and collapsed to one using a custom script (Cusanovich et al. 2018b). Next, read coverage for each cell was calculated, and cell barcodes with fewer than 150 reads were removed from further analysis (Supplemental Fig. S1). The reads that made it through filtering for each batch of sequencing were merged into a single BAM file using the Picard MergeSamFiles program (http://broadinstitute.github.io/picard). Last, the reads in the merged BAM file were converted into cut sites by taking 60-bp intervals centered on the fragment ends, shifting those sites for reads mapping to the forward strand by +4 bp and the negative strand by −5 bp (to account for the shape of the Tn5 cut site) (Buenrostro et al. 2013), and writing the resulting coordinates to a BED file for peak calling.

We called peaks from the cut site data using MACS2 (v. 2.2.5) (Zhang et al. 2008) with the options ‐‐format = BED, -g 9e7, ‐‐nomodel, ‐‐qvalue=0.05, ‐‐SPMR, ‐‐tsize=60, ‐‐bdg, ‐‐keep-dup all, and ‐‐call-summits. Additionally, we aligned and mapped cut sites for some bulk ATAC-seq data collected on naked *C. elegans* genomic DNA and provided these as an input control to correct our peak calls for sequence bias in Tn5 cutting. After peak calling, we merged any overlapping peaks to produce a single set of nonoverlapping genome-wide peak calls. Finally, we generated a binary cells-by-peaks matrix that records which peaks were detected (i.e., were overlapped by a cut site) in each cell. This data structure was used for further cell clustering analysis with LDA, described below.

### Cell clustering pipeline overview

To identify cell types from the sci-ATAC-seq data, we used an iterative clustering and peak-calling approach. After investigating dimensional reduction methods, including PCA and LSI, we found LDA provided the best separation between clusters of cells. The core pipeline consisted of three main steps: first, training a LDA model (see below) on the data; second, identifying LDA topics that corresponded to coherent groups of cells and using those topics to cluster the cells; and finally, calling peaks on pooled data for each cluster of cells. Calling peaks on the cell clusters increases sensitivity to detect cluster-specific peaks compared with the peak calling at the end of the alignment pipeline; cell type–specific regulatory sites may be weaker than more commonly-accessible peaks either because they are smaller or more transiently accessible or because they may be specific to a small subset of the cells in the whole worm. Either way, the data for such sites might not rise above the background noise from other cell types in the whole-worm data set but are detectable in more homogeneous subsets of cells. We iterated this procedure twice: first, to generate a more sensitive set of peaks than the peak calls from the alignment pipeline (we call this first iteration the primary LDA), and a second time to train a new LDA model with the improved peak set, which also results in a third, refined peak set (we will refer to the second iteration as the refinement LDA). Last, MACS2 occasionally calls broad peaks that cover large regions of the genome of hundreds or even thousands of base pairs. The signal over these peaks is usually multimodal with multiple distinct “summits” that most likely represent distinct binding sites. To better capture the distinct nature of these summit regions in the refined peak set, we implemented a custom script that identifies the local summits within a MACS2 peak and splits the peak into multiple contiguous segments that each encompass a single summit region, and we report these split peaks in our UCSC Genome Browser track hubs. (Note that our peak splitting procedure is similar to, but distinct from, the MACS2 summit-calling procedure, which reports the coordinate of the base pair with the highest signal and does not actually segment the peak around the summits.) For the peak-splitting code, see the expand_summits2.py script in the paper's GitHub repository (https://github.com/tdurham86/L2_sci-ATAC-seq) and Supplemental Code.

### LDA implementation

Inspired by the effectiveness of LDA as implemented in cisTopic (González-Blas et al. 2019), we decided to take this approach to analyzing the sci-ATAC-seq data. Briefly, LDA is a Bayesian modeling strategy that was originally developed in the setting of document classification. It assumes that each document is characterized by one or more latent “topics” and that these topics are characterized by subsets of the words in the document. Documents are modeled as Dirichlet probability distributions over the set of topics, and the topics are modeled as Dirichlet probability distributions over the set of words. Training proceeds by iterating over the entire vocabulary defined by the documents it is modeling and proposing a topic for every instance of every word in every document. The probability of picking a topic for a given word and document is computed based on the current probability distribution of topics for that document and the probability of words for each topic. At the end of training, the probability distributions for the topics over the documents and the words over the topics can be calculated by summing the topic proposals for all peaks and for all documents, respectively. Our implementation uses a collapsed Gibbs sampler to speed up training by sampling the latent parameters of the model from the full conditional posterior (Griffiths and Steyvers 2004). When applied to scATAC-seq data, as in cisTopic, cells are treated as the documents and peaks are treated as the words. The LDA model then learns topics that distinguish among the cells based on which peaks tend to be accessible in similar patterns across all cells, and outputs two matrices that capture the relationship between peaks and topics and between cells and topics: The first matrix contains the counts of the number of cells for which a given peak was assigned to each topic (the peaks-by-topics matrix), and the second matrix contains how many peaks from each cell were assigned to each topic (the cells-by-topics matrix).

We began by using cisTopic itself, but found the R implementation to take ∼2 d to process the full data set. (Note that a new recently released version of cisTopic is significantly faster and incorporates similar ideas to our implementation.) To speed up the modeling, we implemented a parallelized version in Java that can split the training of a single model across multiple cores, reducing the run time to just a couple of hours. In the end, for the whole-worm primary LDA, we used 34 topics and set the alpha parameter for the Dirichlet priors to 3.0 to concentrate the probability distributions into just a few peaks/topics. We set the beta parameter to 2000.0; the higher value allows the LDA model to spread the probability across more peaks. These alpha and beta values control the weight of the symmetric, or uniform, priors used for the cell-by-topic and topic-by-peak distributions; the higher the alpha or beta value, the more weight is given to the uniform prior, which encourages the model to spread probability across topics and peaks instead of concentrating it. The Java code can be accessed at GitHub (https://github.com/gevirl/LDA) and is released under the GNU GPLv3 license.

### Filtering the cells-by-peaks matrix for LDA

To improve the efficiency and effectiveness of LDA, it can help to remove cells that have very few peaks and to remove peaks that are either found in very few cells or too many cells. Sparse cells and peaks provide little useful information and are enriched for noise, whereas peaks that are found in too many cells are likely real; they are not very helpful for distinguishing among cell types. We filtered the outliers by sorting the cells by the fraction of all peaks that was detected in each cell (or peaks by the fraction of all cells in which they were detected), mean centering the data, and then identifying the change points at the extremes of the resulting curve by doing a convolution with values from a sigmoid function to detect the areas where the slope is changing fastest. We set the filtering thresholds as four times the inter-quartile range above the mean of the convolution output (Supplemental Figs. S31, S32). We applied LDA to the resulting filtered cells-by-peaks matrix.

### LDA model selection

Choosing hyperparameter values is one of the most challenging aspects of training models like LDA. In particular, using an appropriate number of topics is critical to getting good results, and picking this number requires an empirical approach. In cisTopic (González-Blas et al. 2019), the investigators recommend training several model instances, each with a different number of topics, and choosing the number of topics that gives the best log likelihood of your input data. However, increasing the number of topics adds parameters to the model, which makes the model better able to fit the training data, even if it has already fit the true signal and begins to train on noise (i.e., it is overfitting). Because the cisTopic procedure uses the same data for training and evaluation, it does not test the generalizability of the model parameters and cannot tell when the model starts to overfit. Ultimately, it will recommend using a higher number of topics than can be supported by the data. To identify a suitable number of topics that avoids overfitting, we implemented the following cross-validation procedure.

First, the cells are evenly and randomly split into five disjoint sets for fivefold cross-validation. Then, for each number of topics that we would like to test, five LDA models are trained, with each model training on four of the folds and holding one out for evaluation. Once each model is done training, it estimates the likelihood of the data in the held-out test fold with a Chib-style estimator (Wallach et al. 2009). In this estimation procedure, the peak-topic probabilities learned from the training data are fixed, and then a cell-topic vector is trained for each held-out cell based on the fixed peak-topic probabilities. The log likelihood of each held-out cell is estimated based on sampling from the posterior of the model trained on that held-out cell. We convert these log likelihoods to perplexity, which is defined as follows:

$$\mathrm{perplexity}(w)=\exp\left(-\frac{L(w|\theta )}{N}\right),$$

where *w* is a held-out test cell, L(w|θ) is the log likelihood of that test cell given the LDA model, and *N* is the number of peaks found in that cell. Because perplexity is inversely related to the log likelihood, smaller values are better. The best number of topics to use is the one that produces the lowest mean perplexity from the five held-out sets of test data. It is important to note that LDA is a stochastic modeling technique, and training on the same data with different random seeds will yield similar but different solutions. In addition, our sci-ATAC-seq data are by nature noisy and complex. Thus, the hyperparameter search procedure will not always result in a clear best number of topics to pick. We found that if we trained a model with a few extra topics beyond the optimal number, LDA would largely ignore the extra topics and still put almost all of the probability in a number of topics that approximated the underlying dimensionality of the data. Given that the model appeared robust to some extra topics, we ran our models with 1.5 times the number of topics recommended by the hyperparameter search (Supplemental Figs. S4, S13).

### LDA training

To train the LDA model, we used our parallelized implementation of LDA, which could generally run on the full data set in ∼2 h on a machine with eight cores and 32 GB of memory. Here is an example command line from the whole-worm refinement LDA analysis (see the GitHub repository for full documentation and usage infomation): java -Xms32G -cp LatentDirichletAllocation.jar org.rhwlab.lda.cache.matrix.LDA_CommandLine -lda -a 3.0 -b 2000.0 scatac_data.bow -li 4000 -o ./out/dir -s 1 -t 55 -th 8 -tn 5 -ch 0 -rid 0000 -pe -d topic -st mode -sk 40 -v 1 -id ./out/dir/0000_topics55_alpha3.000_beta2000.000 -pr 1.0.

The model has four main outputs: the docTopic matrix, which contains the raw counts of how many peaks were assigned to each topic in each cell; the theta matrix, which contains the probability distribution across topics for each cell and takes into account the full LDA probability, including the prior; the wordTopic matrix, which contains the raw counts of how many times each peak was assigned to each topic across all cells; and the phi matrix, which contains the probability distribution across peaks for each topic and takes into account the prior. We used the theta matrix to cluster the cells and used the wordTopic matrix for identifying the cell type for each cluster.

### Cell clustering by topics

Next, we sought to cluster the cells based on the LDA modeling results. We reasoned that differences among cell types would be the dominant source of informative variation in our sci-ATAC-seq data and that, for this reason, many topics should mostly correspond to distinct cell types. To identify which topics were most likely to distinguish among cell types, we looked for topics that had high probability in subsets of cells that were close together in LDA topic-space and used these topics to define cell clusters as follows. To identify topics that corresponded to groups of cells in LDA topic-space, for each topic, we ranked the cells by their probability for that topic in the theta matrix, computed the centroid of the topic as the mean topic probability vector for the top 50 cells, and then scored the topic by calculating the average similarity of those top 50 cells to the centroid by averaging the dot products of the topic probability vectors and the mean topic probability vector. Then, we ranked the topics by this centroid similarity and identified a threshold of 0.2 to separate the candidate cell type topics from the others (Supplemental Fig. S5).

Next, we assigned cells to clusters defined by these topics. Any cell with >50% probability in one of the cell type topics was automatically assigned to that topic cluster. Some topic clusters had many high probability cells, whereas some had few. For any clusters with fewer than 150 cells, we attempted to add nearby unassigned cells based on their distance from the cluster's centroid in a 10-dimensional UMAP space (McInnes et al. 2018). We used an iterative procedure for each small topic cluster as follows: First, we computed the cluster centroid in UMAP space by averaging 200 samples from a Gaussian kernel density estimate of the shape of the cluster. Next, we used a KDTree to identify a set of nearest neighbor cells to the centroid that was 25% larger than the cluster size. We detected whether any of these nearest neighbors were distance outliers (and thus more likely from a different cluster) by ranking the neighbors by distance from the centroid and convoluting the resulting distances with a step function to detect regions where the slope of the ranked distances increases. We added to the topic cluster the unassigned cell closest to the centroid as long as its value from the convolution was not greater than 1.5 times the interquartile range of all convolution values. Then we iterated this procedure, growing the small topic clusters one unassigned cell at a time. After this procedure, any topic clusters that still had fewer than 50 associated cells were removed from consideration as clusters, and their cells were not assigned to any cluster.

To visualize the resulting topic clusters, we used UMAP (McInnes et al. 2018) to reduce the dimensions of the theta matrix to two (Fig. 3C). We first row-normalized the theta matrix with the L2-norm and then used the Python implementation of UMAP (umap-learn, v. 0.3.8) with default parameters. Finally, we plotted the cells as a scatter plot based on their coordinates in 2D UMAP space, and we colored the cells by their topic cluster assignments.

### Calling peaks for each topic cluster

The final step in the clustering pipeline is to call peaks for each cluster. For each topic cluster, we pooled the cut sites from the cells in that cluster and used the pooled data as inputs to MACS2 (v. 2.2.5) (Zhang et al. 2008) with the same settings as in the alignment pipeline, including providing the bulk ATAC-seq data from naked genomic DNA as input controls. After calling peaks for each cluster, we merged the peak calls from all clusters using bedtools merge to make a master list of peak regions and then used this master peak list to create a new cells-by-peaks matrix. We used our new cells-by-peaks matrix as input to a second round of LDA and cell clustering to refine our clusters and peak calls.

### Overlapping peaks with other data sets

Supplemental data for Figure 2 from Jänes et al. (2018) was downloaded from the eLife website (filename: janes2018_fig2_data1_v2.txt). This file was parsed using Unix tools and BEDTools (v. 2.25.0) (Quinlan and Hall 2010) into three files: a file containing all of the peaks in BED format with overlapping sites merged (i.e., using bedtools merge with default parameters), a file containing promoter-annotated peaks (those annotated as “coding_ promoter,” “unassigned_promoter,” or “pseudogene_promoter”) with overlapping peaks merged, and a file containing enhancer-associated peaks (those annotated as “putative_enhancer”) with overlapping peaks merged. These files were overlapped with the sci-ATAC-seq peaks, and the sci-ATAC-seq peaks were overlapped with these files, using bedtools intersect. The significance of the extent of the overlapping peaks was calculated using the Fisher's exact test implementation in the bedtools fisher command.

Peak loci from the modERN project were downloaded from the EPIC website (http://epic.gs.washington.edu/modERN/) for reference WS245/ce11 using the “download aggregated peaks” and “download clustered peaks” buttons on the “worm by life stage” tab of the user interface. Peaks were parsed into different files based on developmental stage, and any overlapping peak regions were merged in the final files. As above, these files were overlapped with the sci-ATAC-seq peaks, and the sci-ATAC-seq peaks were overlapped with these files using bedtools intersect. The significance of the extent of the overlapping peaks was calculated using the Fisher's exact test implementation in the bedtools fisher command.

To test for enrichment of overlaps from bulk ATAC-seq sites or TF ChIP-seq sites from different developmental stages, we split the sites from each compendium into separate BED files by developmental stage, from embryo to young adult. For the TF ChIP-seq data, this split was accomplished using the developmental stage annotation provided in the downloaded peaks. For the bulk ATAC-seq peaks, we first z-score-normalized the signal heights for each peak across life stages provided in the Figure 1 supplemental data from Jänes et al. (2018), and we then assigned each peak to any developmental stage with a *z*-score ≥ 1.5 for that peak and wrote the assigned peaks to the corresponding BED files. Next, we counted the number of overlaps between the developmental stage beds and our sci-ATAC-seq peaks and then computed the log_2_ ratio of the number of overlaps observed versus the number of overlaps expected from 100 randomly drawn samples of sites (Fig. 2C; Supplemental Fig. S2).

We also downloaded ChIP-seq data for the L2 developmental stage from Jänes et al. (2018) (NCBI Gene Expression Omnibus [GEO; https://www.ncbi.nlm.nih.gov/geo/] accession GSE114440) and analyzed the ChIP-seq signal over sci-ATAC-seq peaks as follows. First, we used the UCSC liftOver tool to map our peak coordinates to ce10 to match the reference of the ChIP-seq data and then generated a set of randomly shuffled peak locations using the bedtools shuffle command with options -chrom and -noOverlapping. Next, for both the true and shuffled peak sets, we computed the log_2_ ratio of the mean signal over each peak to the mean signal over the entire chromosome for each ChIP-seq data set, and then we compared the resulting distributions using split violin plots (Supplemental Fig. S3).

To gain further insight into our sci-ATAC-seq peaks that did not have overlaps with either TF ChIP-seq sites or with bulk ATAC-seq sites, we reasoned that if our nonoverlapping peaks are the result of the increased sensitivity of sci-ATAC-seq for tissue-specific accessibility, then our lists of the top 250 most topic-specific peaks (Supplemental Fig. S7) should be depleted for overlaps with TF ChIP-seq sites and bulk ATAC-seq sites. For each topic, we calculated the fraction of the 250 most topic-specific peaks with an overlap and compared that fraction to the overall fraction of sci-ATAC-seq peaks with overlaps in the TF ChIP-seq sites and bulk ATAC-seq sites using the log_2_ ratio (Supplemental Fig. S10). We find that, for most topics, the top topic-specific peaks are depleted for overlaps compared with the full list of peaks. The neuron-specific topics are particularly depleted for overlaps with bulk ATAC-seq sites, suggesting that whole-worm bulk ATAC-seq has reduced sensitivity for detecting neuron-specific peaks (Supplemental Fig. S10B). In addition, other important technical differences in the data collection methods could also contribute to differences in the regulatory sites detected. For example, in the case of the ChIP-seq data, the recovered sites are biased for the particular set of TFs that are assayed; in contrast, sci-ATAC-seq is relatively unbiased but is subject to the Tn5 sequence bias (Green et al. 2012).

### Cell-by-topic and peak-by-topic heatmaps

To generate the heatmaps as in Figure 3B and Figure 6, we selected only the columns corresponding to the topics used for clustering the cells. Next, we row-normalized the counts by dividing each row by its sum. Then we hierarchically clustered the rows and columns in Python (v. 3.6.10) with the SciPy module (v. 1.4.1). We used the scipy.spatial.distance.pdist function with the cosine metric to compute the pairwise distance matrix, then scipy.cluster .hierarchy.linkage with method average to generate clusters, and finally scipy.cluster.hierarchy.dendrogram to order the rows and columns after clustering. The clusters were classified by tissue type based on the dominant tissue type for each topic in Figure 5. The cell/peak coverage information was calculated as the log_2_-transformed sum of peaks found per cell for the cell-by-topic matrix or the log_2_-transformed sum of cells showing a peak for the peak-by-topic matrix.

### Identifying tissue-specific topics

We identified topic tissue specificity in two ways: by overlapping peaks from each topic with peaks from ChIP-seq of cell type–specific factors (Fig. 4) and by assigning peaks to the nearest gene and assessing which tissues those genes tend to be expressed in (Fig. 5).

To compare the overlap of topic-associated sci-ATAC-seq peaks with TF ChIP-seq peaks, we created separate BED files for peaks from ChIP-seq experiments for HLH-1, ELT-1, or ELT-2; removed any peaks that had overlaps in 40 or more other ChIP experiments (i.e., high occupancy target, or HOT, sites); and merged any remaining overlapping peaks with bedtools merge. We intersected the peaks called from each topic cluster with the peaks from each TF and recorded the number of overlapping peaks for each topic. To understand whether the observed overlaps per topic were surprising, we generated a null distribution by sampling a number of sci-ATAC-seq peaks equal to the number of observed overlaps, with each peak being drawn from a particular topic with a probability based on the total number of peaks called for that topic cluster. We then took the log_2_ ratio of the topic distribution of observed overlaps to the topic distribution of each sample of randomly drawn peaks. We plot the mean log_2_ ratio and use the samples to compute a 95% confidence interval around each bar (Fig. 4). We expanded our analysis and repeated this same procedure for all TF ChIP-seq data sets, and we present the results as a heatmap in Supplemental Figure S6.

We also used previously published sci-RNA-seq data (Cao et al. 2017) to comprehensively identify cell types for our topic clusters by assessing the tissue expression patterns of genes near our topic-specific peaks. For each topic, we ranked the peaks by their topic specificity, which we define for a given peak and topic as the fraction of cells with evidence for that peak that are members of the topic's cluster. We wrote a BED file for each topic cluster that contained the coordinates of either the top 250 peaks by topic specificity or all peaks with topic specificity greater than 0.5, whichever was greater (Supplemental Fig. S7). Next, we associated these topic-specific peaks with their nearest downstream gene (within 1200 bp) by using bedtools closest with options -D b, -io, and -id. Similar to the cell type–specific TF ChIP-seq analysis above, we then asked whether the expression distribution of these top genes across tissues is enriched on average for particular tissues. Accordingly, we drew 100 samples of 250 genes from the null distribution of all genes with the sci-RNA-seq data, and we compared the expression distribution of these gene sets with our sets of topic-specific genes by computing the log_2_ ratio of the mean topic-specific tissue expression distribution to the mean tissue expression distribution of each random sample. We again reported the mean log_2_ ratio and computed the 95% confidence interval around the mean. For an example illustrating this procedure, see Supplemental Figure S8, and for the tissue expression distributions associated with all 37 topic clusters, see Supplemental Figure S9. Note that we tried using different numbers of topic-specific peaks, from the top 50 to the top 500, and found that the cell type identities ultimately assigned to the topics were rather insensitive to this choice. We decided to use 250 as a balance between having input from as many potential cell type–specific genes as possible without including too many non-topic-specific peaks (Supplemental Fig. S7).

### Identifying tissue subtypes using marker genes

To identify fine-grained cell types, we conducted a subclustering analysis for each of the following major worm tissues that we identified: coelomocyte (Supplemental Figs. S21, S22), glia (Supplemental Figs. S23, S24), gonad (Supplemental Figs. S25, S26; Kimble and Crittenden 2005), hypodermis (Supplemental Figs. S27, S28), intestine (Fig. 8; Supplemental Fig. S15), muscle (Fig. 8; Supplemental Fig. S14), neuron (Fig. 9; Supplemental Figs. S17, S18, S19, S20, S16), and pharynx (Supplemental Figs. S29, S30). There were too few cells identified as sex myoblast to conduct a subclustering on that tissue. First, we pooled the data for the cells in the topic clusters corresponding to each tissue and also merged the peaks called from these topic clusters to create a data set per tissue (Table 1).

Next, we ran the same iterative LDA-modeling and cell-clustering procedure as detailed above for the whole worm. We identified subclusters by coloring the UMAP scatter plots by the cells with peaks near cell type–specific marker genes (see Figs. 8, 9). We colored a cell for a marker gene if it showed evidence for any peak that either overlapped the gene body or overlapped the region from 1200 bp upstream (5′) of the gene to 100 bp downstream (3′) from the gene. The marker genes we plot were used to identify fine-grained cell types in embryonic and L2 *C. elegans* scRNA-seq data (Packer et al. 2019).

### Comparing peak versus gene tissue specificity

To summarize the tissue specificity of peaks and genes, for each gene we calculated the entropy of the distribution of expression levels across tissues in the scRNA-seq, and for each sci-ATAC-seq peak, we calculated the entropy of the distribution of number of other overlapping peaks from each tissue. The entropy of a discrete distribution is a measure of how concentrated the probability of that distribution is in some subset of the elements of the distribution; for any domain, the uniform distribution has the highest possible entropy, and a distribution with 100% of the probability at one point in the domain has the lowest possible entropy. Thus, low entropy of a peak or gene indicates high tissue specificity, and high entropy indicates low tissue specificity. To compare gene expression patterns with their corresponding accessibility patterns, we assigned all peaks to their nearest downstream exon within 1200 bp (allowing overlaps), and then for each gene, we averaged the entropy values of all peaks linked to the exons of that gene. Plotting these values against each other for 13,111 genes suggests that most genes have a lower mean peak entropy than expression entropy, indicating a more tissue-specific pattern of regulation than their pattern of expression (Supplemental Fig. S11). Some genes with high expression entropy and low accessibility entropy simply do not have much chromatin accessibility signal, whereas other examples show extensive chromatin accessibility that shows multiple tissue-specific patterns that independently have low entropy and thus lead to a low mean peak tissue entropy for that gene. To distinguish between these two cases, we calculated a peak diversity score for each gene, which is defined as the number of distinct overlap patterns among the peaks from different tissues assigned to that gene. For example, consider a hypothetical gene that has a neuron peak, an intestine peak, and a pharynx peak. Assume that the neuron peak does not overlap any other peaks, and the intestine and pharynx peaks do overlap. That gene would have a peak diversity score of two: There is one pattern of neuron-specific accessibility and another of intestine plus pharyngeal accessibility. We extracted the set of 2038 genes with peak tissue entropy less than 1.0 and expression entropy greater than 1.5, which are enriched for the genes with the broadest expression but most tissue-specific gene regulation, and provide them as Supplemental Table 1.

### Generating UCSC Genome Browser tracks for the topic clusters

To generate signal files in bigWig format for display in the UCSC Genome Browser, we used the MACS2 output from our peak-calling steps. The data from the input control bedGraph file were subtracted from the data in the treatment bedGraph using macs2 bdgcmp -m subtract, and the resulting bedGraph file was converted to bigWig format using the bedGraphToBigWig utility from UCSC (Kent et al. 2010). These bigWig tracks are displayed along with our peak calls in two track hubs on the UCSC Genome Browser:

- sci-ATAC-seq Main Track Hub URL—http://genome.ucsc.edu/cgi-bin/hgTracks?db=ce11&hubClear=http://waterston.gs.washington.edu/atacTissue/Durham_hub.txt; and
- sci-ATAC-seq Subclustering Track Hub URL—http://genome.ucsc.edu/cgi-bin/hgTracks?db=ce11&hubClear=http://waterston.gs.washington.edu/atacCellType/Durham_hub.txt.

## Data access

All raw and processed sequencing data generated in this study have been submitted to the NCBI Gene Expression Omnibus (GEO; https://www.ncbi.nlm.nih.gov/geo/) under accession number GSE157017. The parallel LDA code is available at GitHub (https://github.com/gevirl/LDA) and as Supplemental Code and is released under the GNU GPLv3 license. The data pipeline, scripts for performing the LDA clustering analysis, and Jupyter notebooks for generating figures are available at the following GitHub repository (https://github.com/tdurham86/L2_sci-ATAC-seq) and as Supplemental Code and are released under the MIT license.

## Supplementary Material

Supplemental Material
