# Supplementary material for: Comprehensive characterization of tissue-specific chromatin accessibility in L2 Caenorhabditis elegans nematodes
Source: Genome Res. 2021 Oct;31(10):1952–69. doi: 10.1101/gr.271791.120 (PMC8494234; doi:10.1101/gr.271791.120)
Supplement: Supplemental Material [file supp_31_10_1952__DC1.html]

Comprehensive characterization of tissue-specific chromatin accessibility in L2 Caenorhabditis elegans nematodes — Supplemental Material 

# Comprehensive characterization of tissue-specific chromatin accessibility in L2 *Caenorhabditis elegans* nematodes

## Supplemental Material

- Supplementary\_Code.zip
- Supplementary\_LDA\_Code.zip
- Supplementary\_Table\_1.xlsx
- Supplementary\_Material.pdf
