# Supplementary material for: Comprehensive characterization of tissue-specific chromatin accessibility in L2 Caenorhabditis elegans nematodes
Source: Genome Res. 2021 Oct;31(10):1952–69. doi: 10.1101/gr.271791.120 (PMC8494234; doi:10.1101/gr.271791.120)
Supplement: Supplemental Material [file supp_gr.271791.120_Supplemental_Material_.pdf]

Supplementary Information

Durham, et al. Comprehensive characterization of tissue-specific chromatin accessibility in L2 *Caenorhabditis elegans* nematodes. *Genome Research.* 2021.

Supplementary Notes

1. **List of 39 genes expressed predominantly in two tissues with accessibility suggesting tissue-specific isoform usage.**

   C30F2.2, F19C7.8, H05L14.2, R13H7.2, T06A4.1, T07D4.2, T23E7.2, T28B4.1, W02B8.1, Y50D4B.1, *abts-3, acox-1.1, aexr-2, atic-1, avr-15, che-12, dac-1, elt-1, exp-2, hmit-1.3, hot-1, ifb-1, kvs-5, lev-11, lin-2, lron-9, mca-1, mua-3, nhx-9, pal-1, pkc-2, pnp-1, sorb-1, twk-17, twk-43, ugt-29, unc-2, unc-7, unc-87*

Supplementary Tables

Supplementary_Table_1.xlsx - 2,038 genes that are broadly expressed across tissues, but that show tissue-specific chromatin accessibility patterns.

Supplementary Files

1. Supplementary_Code.tgz - This file contains all of the code for running data and analysis pipelines, along with Jupyter notebooks for reproducing paper figures. It can also be found at https://github.com/tdurham86/L2_sci-ATAC-seq.

2. Supplementary_LDA_Code.tgz - This file contains the latent Dirichlet allocation implementation. It can also be found at https://github.com/gevirl/LDA.

Supplementary Figures

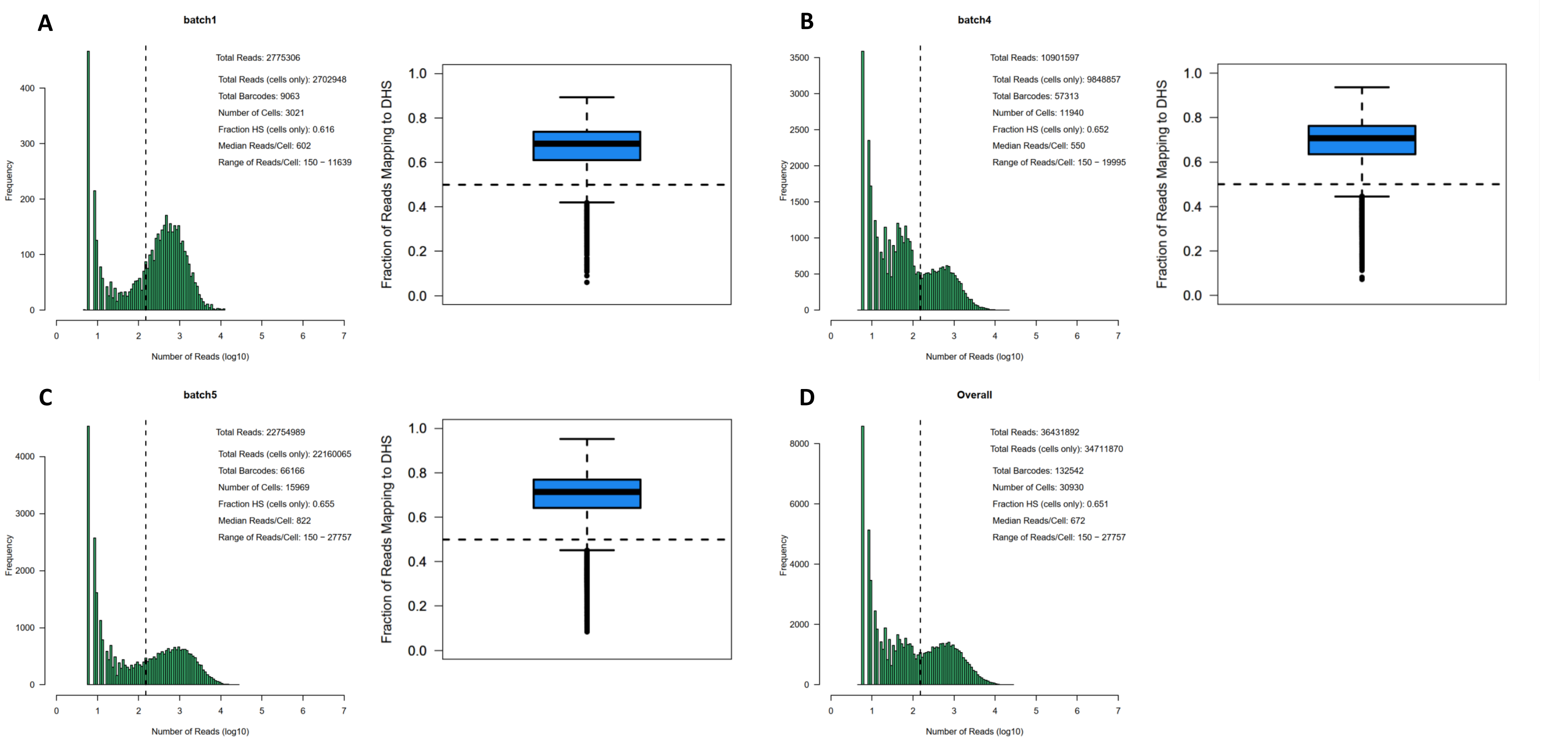

Supplementary Figure S1: **After thresholding cell coverage distribution, we recover a total of 30,930 cells from three sequencing batches.** We show the histograms of unique reads per cell barcode and the distribution of the fraction of reads mapping in a peak region for each sequencing batch, including (*A*) the initial pilot batch (MiSeq sequencing), (*B*) large-scale batch number 1 (NextSeq), and (*C*) large-scale batch number 2 (NextSeq). The final panel (*D*) shows the aggregated read coverage statistics for all three batches.

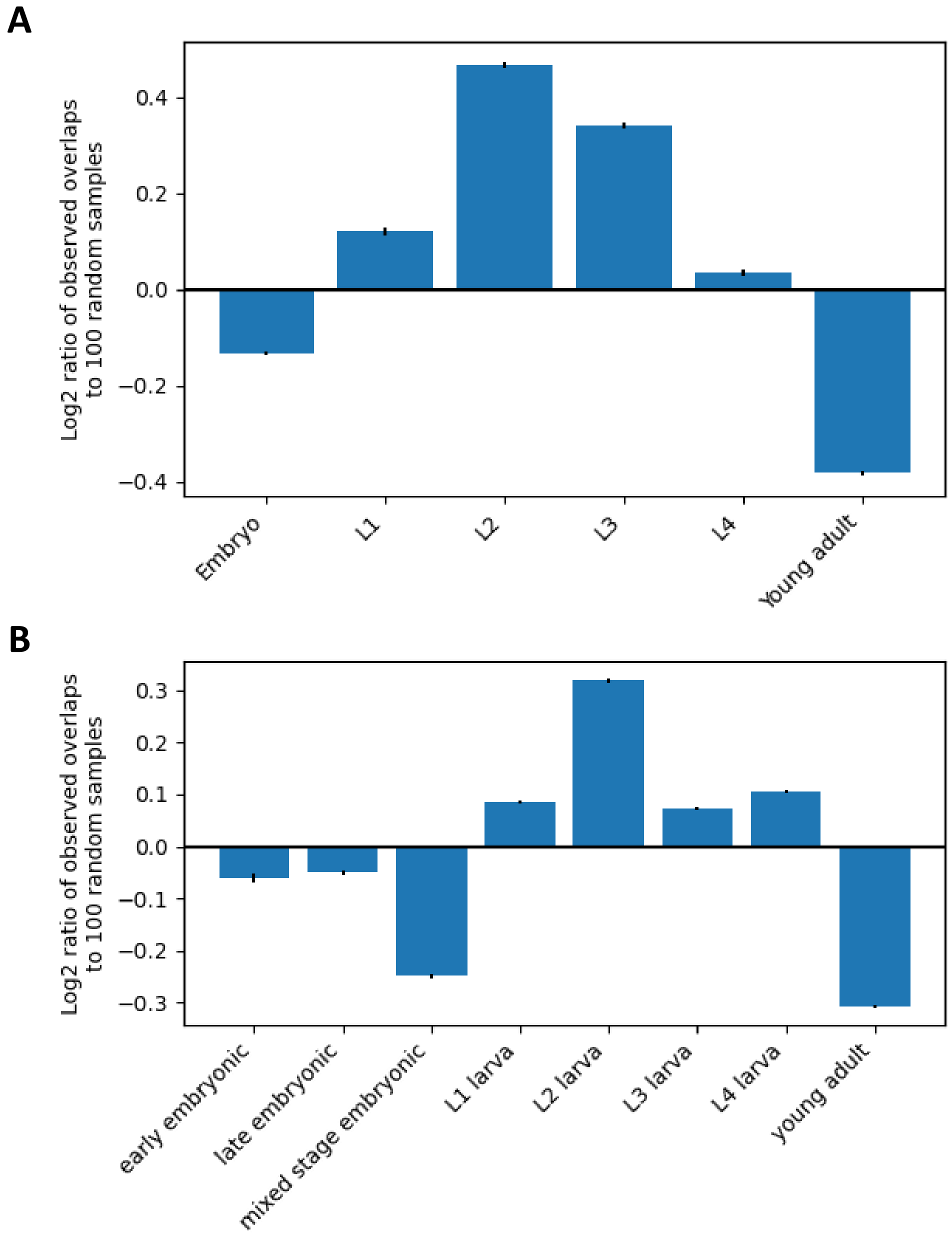

Supplementary Figure S2: **sci-ATAC-seq peaks are enriched for overlaps with bulk ATAC peaks and singleton ChIP-seq sites from the L2 stage.** Similarly to Fig. 2C, we computed the fraction of sites from either (*A*) bulk ATAC-seq (Jänes et al., 2018) or (*B*) singleton TF ChIP-seq sites (Kudron et al., 2018) from different developmental stages, and compared this to a randomly-drawn null distribution of overlaps across developmental stages using the $\log_2$ ratio. Error bars show the 95% confidence interval from comparison to 100 random samples.

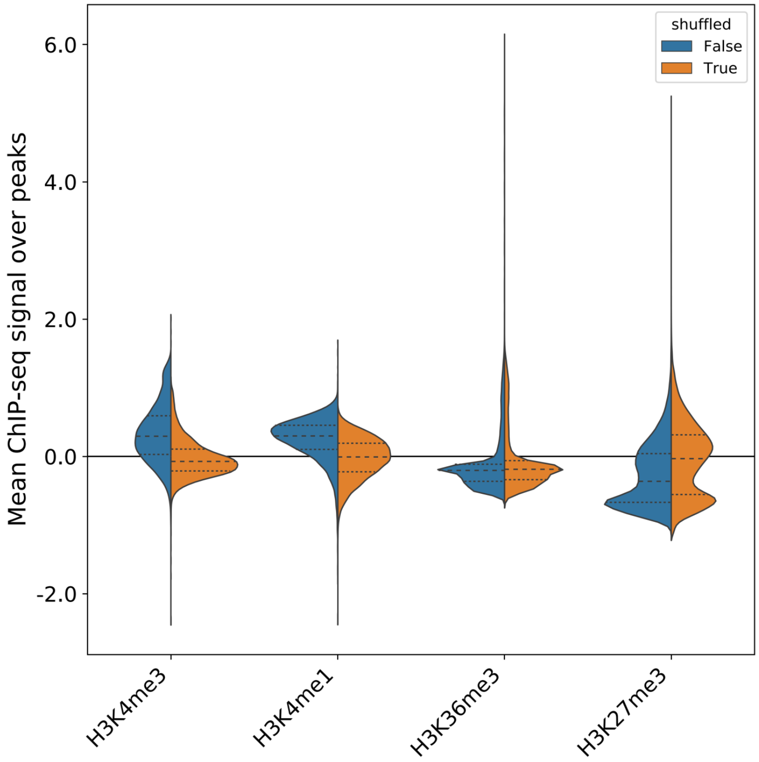

Supplementary Figure S3: **sci-ATAC-seq peaks tend to be over regions with histone marks associated with gene activation.** For each sci-ATAC-seq peak, we computed the average ChIP-seq signal from four histone modification data sets collected in L2 worms Jänes et al. (2018), took the $\log_2$ ratio of each peak's signal to the mean ChIP-seq signal across the whole chromosome, and then plotted the distribution of these ratios (blue side of each violin plot). We then randomly shuffled the sci-ATAC-seq peak regions and re-computed the same peak-mean to chromosome-mean ratios (orange side of each violin plot). The horizontal dotted lines in each violin plot show the quartiles of the distributions. Compared to randomly-shuffled locations, sci-ATAC-seq peaks are enriched for H3K4me3 and H3K4me1 histone marks, which are associated with active regulatory regions; uncorrelated with the H3K36me3 histone mark, which is found over actively transcribed gene bodies; and depleted for signal from the repressive H3K27me3 histone mark.

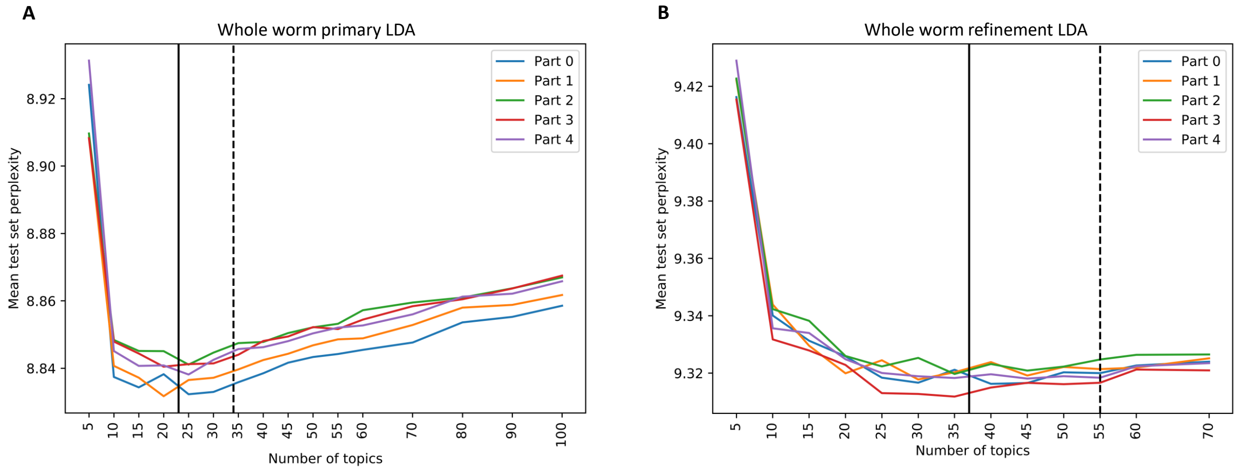

Supplementary Figure S4: **Tuning the number of topics using 5-fold cross validation.** Models were trained on 4 folds and tested on a held-out fold for varying numbers of topics. The average minimum topic number (solid line) was calculated, and used as the basis to pick a number of topics 1.5 times greater for use in training the full LDA model (dotted line). (*A*) Topic number search for the whole-worm primary LDA. (*B*) Topic number search for the whole-worm refinement LDA.

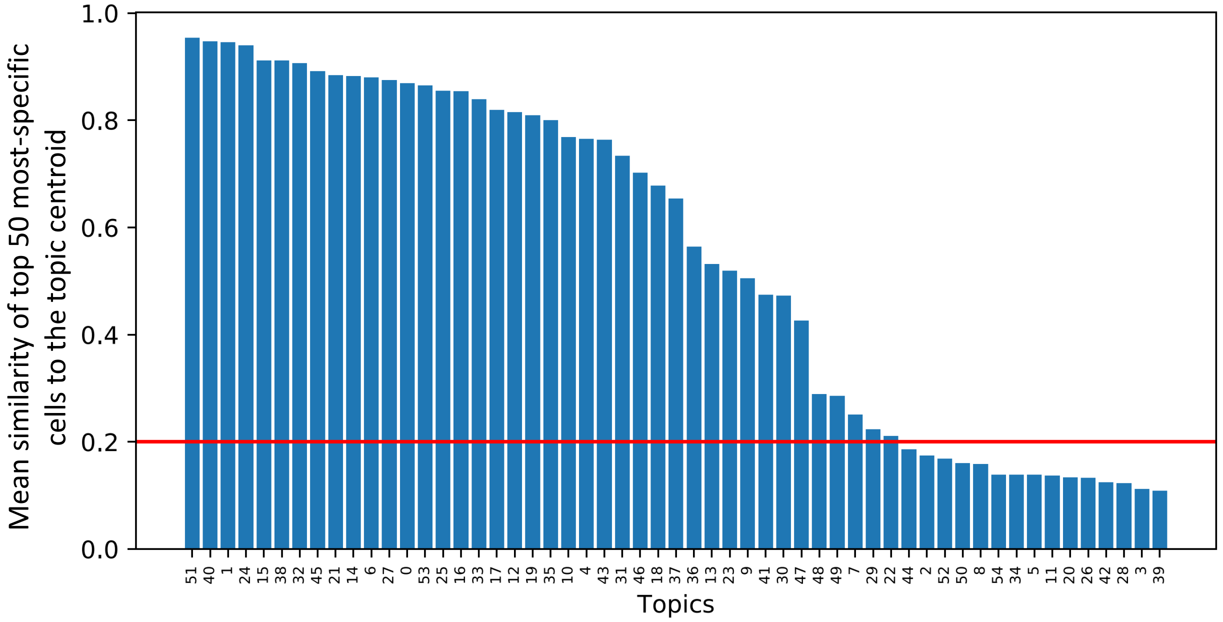

Supplementary Figure S5: **Identifying topics that capture clusters of cells.** Topics were ranked by the mean similarity of their top 50 most-specific cells to the average topic distribution of those same 50 cells. Topics were considered for further analysis if their mean similarity exceeded the 0.2 threshold (red line).

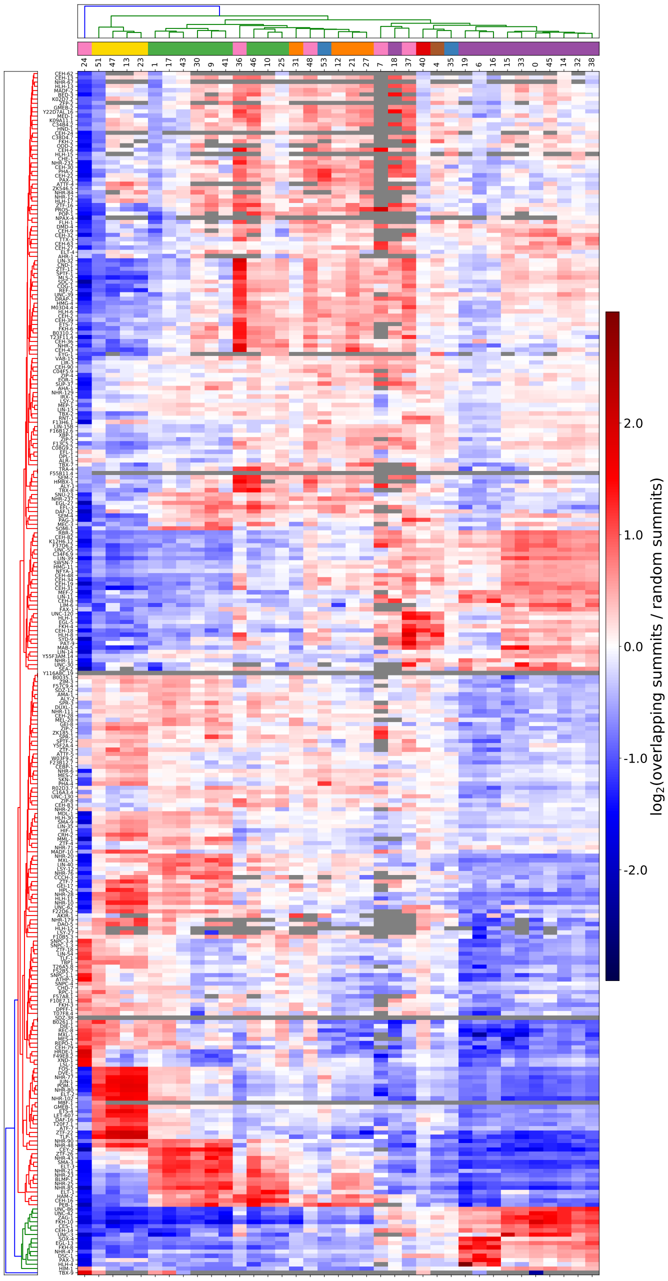

Supplementary Figure S6: **Sci-ATAC-seq peaks called from the whole-worm refinement LDA analysis show enrichment and depletion for overlaps with TF ChIP-seq peaks that is consistent with the TF tissue-specificity.** We extended the analysis shown in Fig. 4 to all TFs with ChIP-seq data. The heatmap shows the mean $\log_2$ ratio of the overlap counts for the peaks called based on each LDA topic to 500 randomly sampled sets of matched size from each topic. Each row displays the results for a single TF across all 37 topics used for clustering in the refinement LDA analysis. The rows and columns are hierarchically clustered based on Euclidean distance, using `sklearn.metrics.pairwise.nan_euclidean_distances` to ignore any TF/topic pairs with no overlaps (grey in the heatmap). TFs with similar tissue-specific expression patterns tend to show similar enrichment and depletion patterns for overlaps with peaks from different topics, and these patterns are consistent with our inferred tissue identities for the topics (Figs. 5, S9).

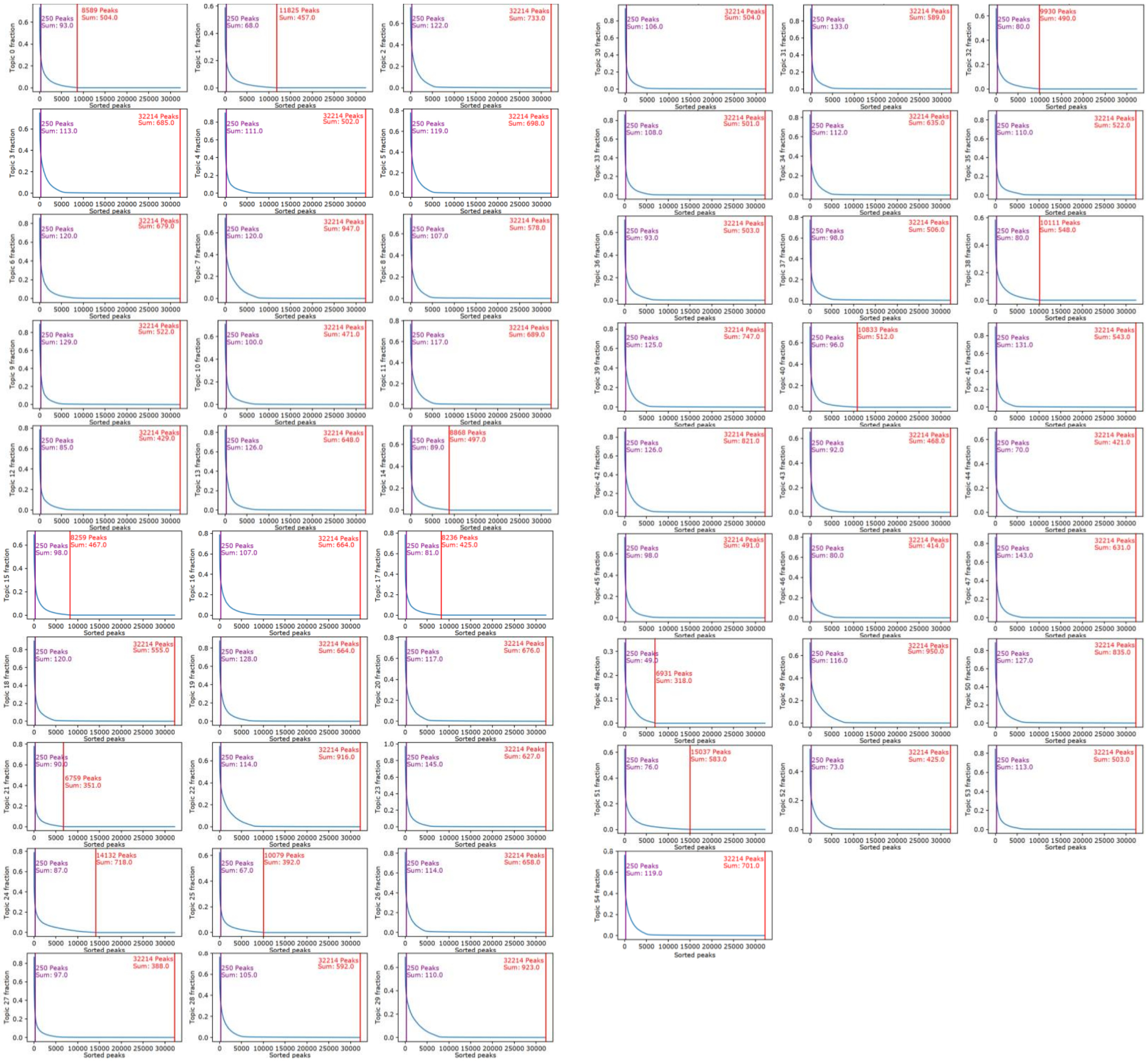

Supplementary Figure S7: **Selecting the top 250 most specific peaks for each topic.** For each topic, ranking peaks by the fraction of cut sites from each peak that LDA assigned to that topic reveals that a small proportion of peaks are highly-specific to each topic. The purple vertical lines show the 250 peak threshold that we used for selecting peaks to cross-reference with the scRNA-seq data for associating cell types with the different topics. The red vertical lines show where the weights of the peaks for each topic reach zero; any peaks to the right of the red line have no cut sites assigned that topic. Note that this figure includes all topics from the whole worm refinement LDA model, regardless of whether they were later used for clustering or not.

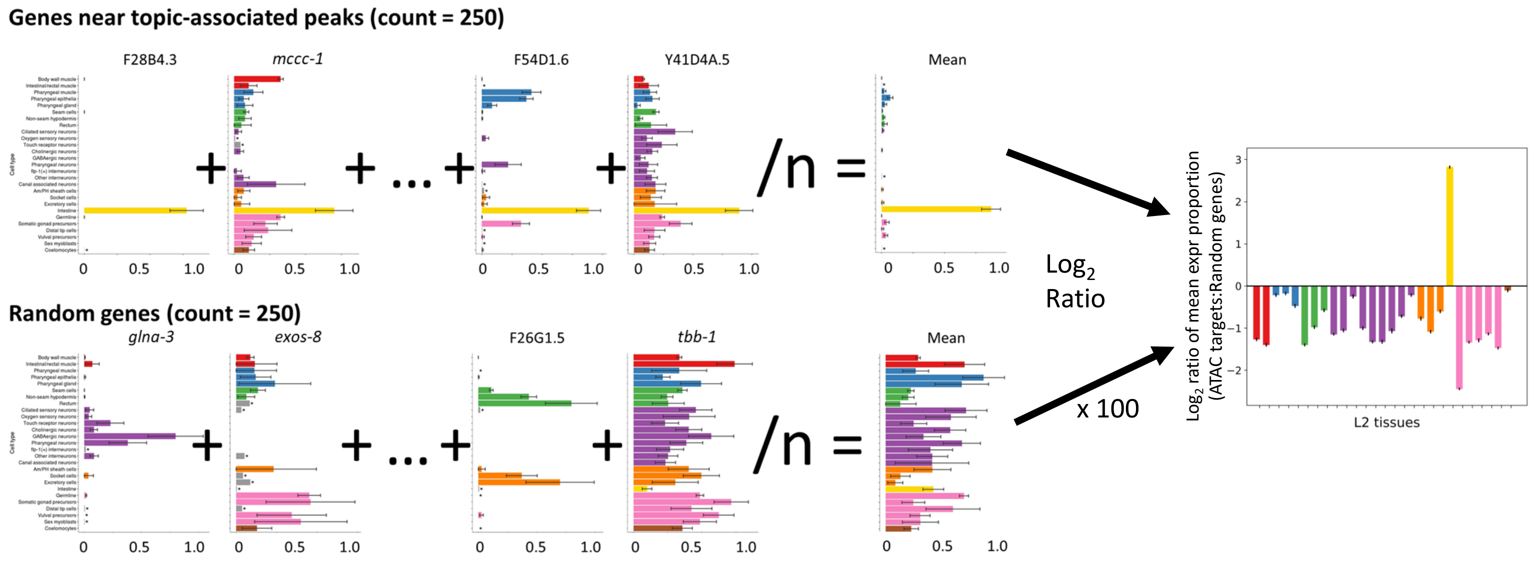

Supplementary Figure S8: **Schematic describing how to compute tissue enrichment.** For Fig. 5, we computed the tissue enrichment values by normalizing the tissue expression values for each gene to sum to one, then calculating the $\log_2$ ratio of the mean expression distribution for the top 250 genes by peak topic-specificity to the mean tissue expression distribution of 250 randomly-selected genes. This was repeated for 100 random samples of 250 genes, and the mean $\log_2$ ratio was plotted with error bars indicating the 95% confidence interval for the enrichment of each tissue.

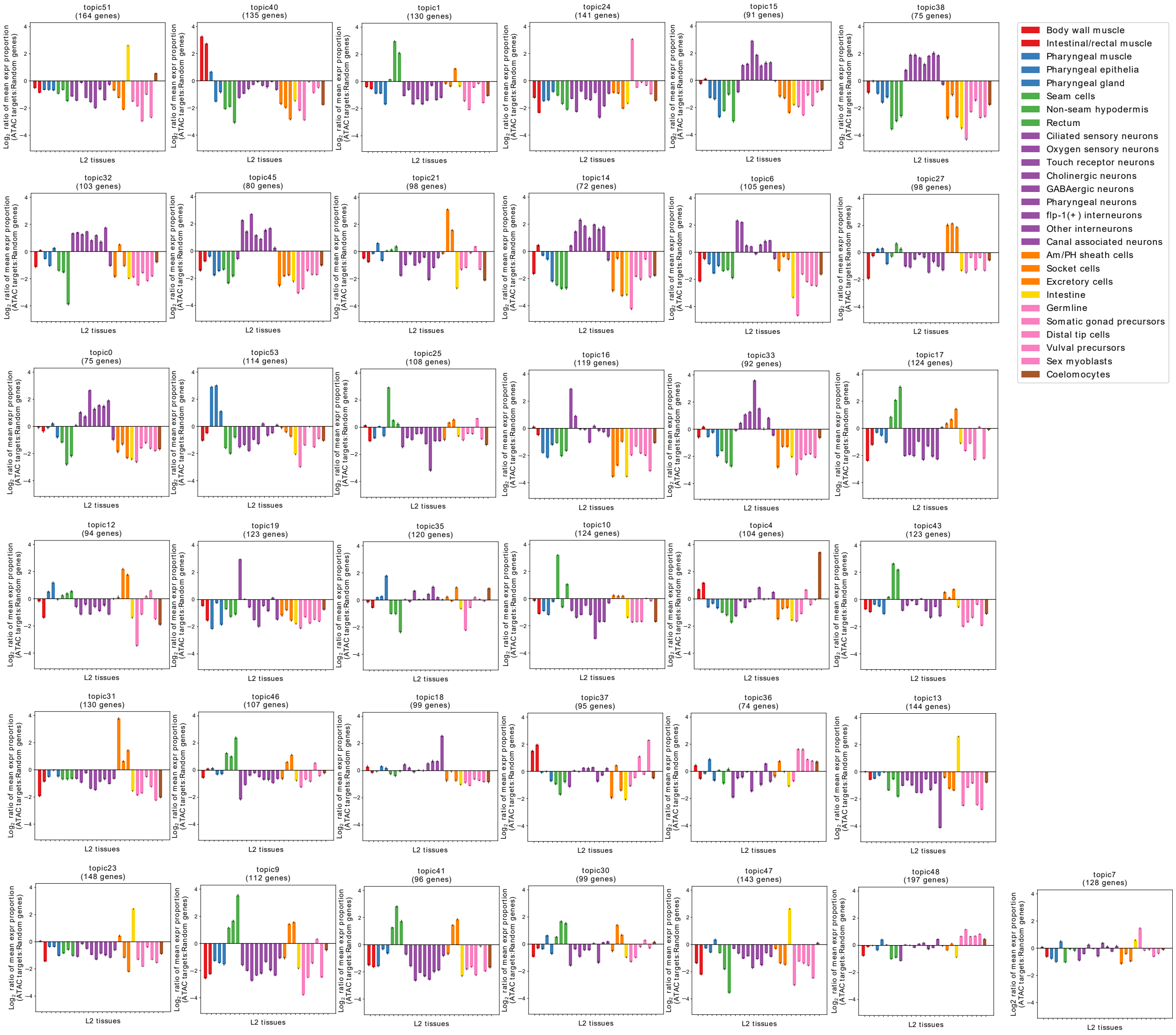

Supplementary Figure S9: **RNA-seq tissue expression enrichment for all 37 topic clusters.** The mean tissue expression distribution of the nearest downstream genes to the top 250 most topic-specific peaks are compared to 100 random samples of 250 peaks (see Methods and Fig. S8). Expanded from Fig. 5 to show results for all 37 topic clusters.

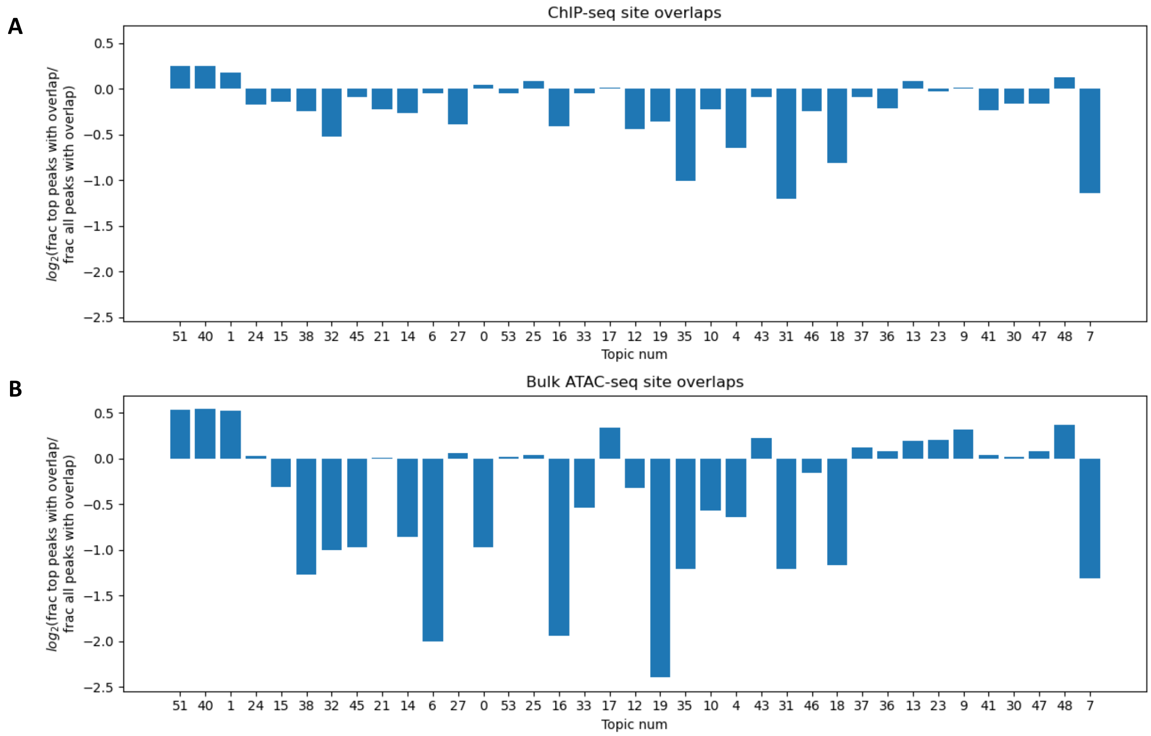

Supplementary Figure S10: **The most topic-specific peaks tend to be depleted of overlaps from other data sets.** We counted the fraction of the top 250 most specific genes (Fig. S7) that overlap a TF ChIP-seq site (*A*) or a bulk ATAC-seq site (Jänes et al., 2018) (*B*) and compared these fractions to the total fraction of sci-ATAC-seq peaks with overlaps (Fig. 2A) using the $\log_2$ ratio. For most topics, the highly specific peaks are depleted for overlaps relative to all sci-ATAC-seq peaks. The topics are ordered on the x-axis by their cluster density from Fig. S5.

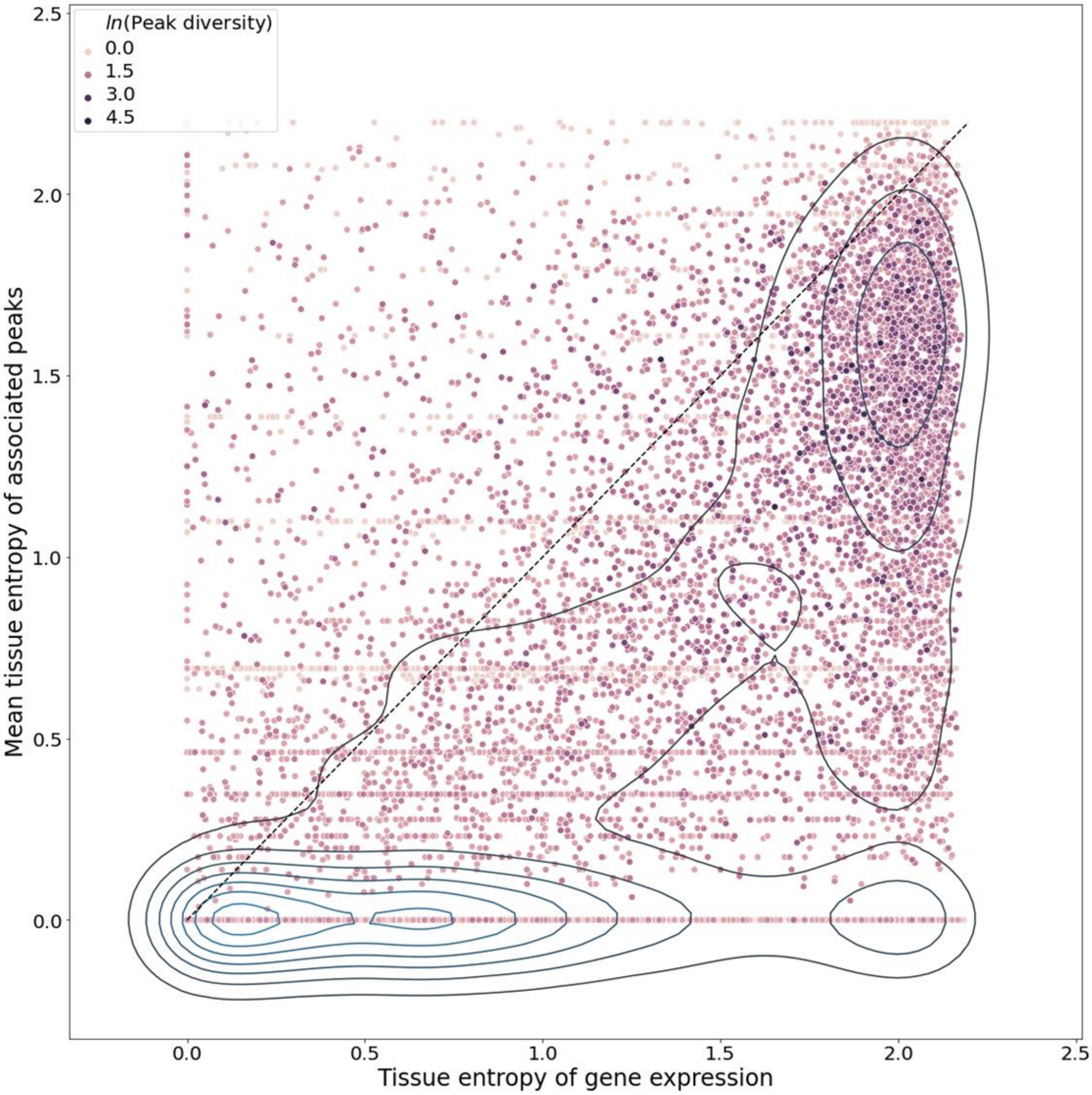

Supplementary Figure S11: **Proximal chromatin accessibility patterns suggest tissue-specific gene regulation of broadly expressed genes.** We calculated the entropy of the tissue expression distribution for each gene in the scRNA-seq, and the entropy of the number of overlaps with peaks from different tissues for the peaks within 1200 bp of each gene. Lower entropy scores indicate more tissue-specificity. We visualized the data for 13,111 genes as two-dimensional kernel density estimate plot. We colored the scatter plot by the peak diversity score of each gene, which we define as the number of unique combinations of overlapping peaks from different tissues that are associated with each gene. Genes with high diversity scores tend to have many different peak arrangements involving multiple tissue types, suggesting a more complex regulatory environment at that gene.

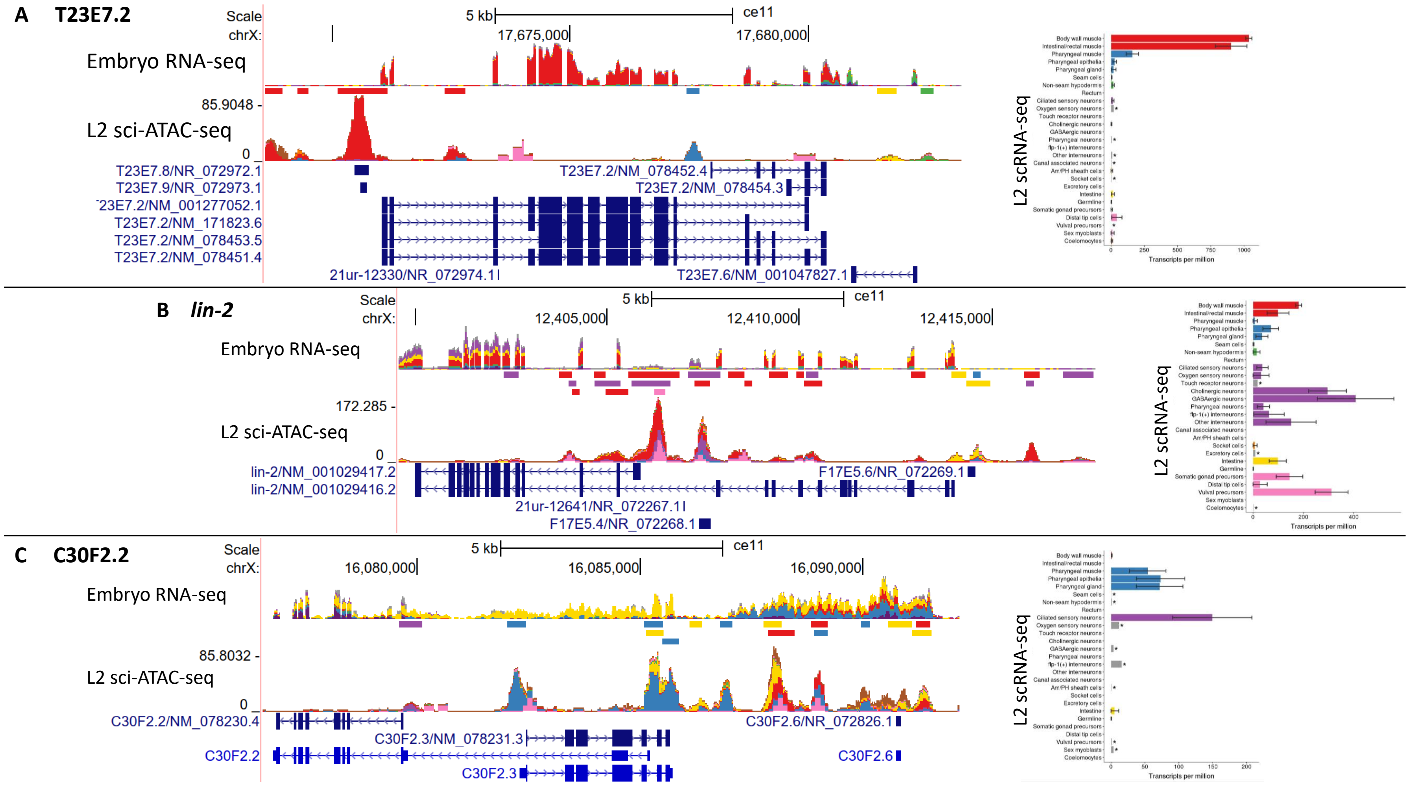

Supplementary Figure S12: **Some genes with multiple start sites show promoter accessibility that suggests tissue-specific isoform usage.** (*A*) Gene T23E7.2 is predominantly expressed in muscle and pharynx according to scRNA-seq. There is a large peak of accessibility in muscle cells (red) at the start of the long isoform, and a smaller peak of pharynx accessibility (blue) at the start of the medium/short isoform. The FACS embryo RNA-seq Warner et al. (2019) shows elevated pharyngeal contribution to the global expression in the last two exons. (*B*) Gene *lin-2* shows a broader expression pattern across tissues, with the accessibility pattern suggesting the long isoform is expressed in muscle (red), intestine (yellow), and pharynx (blue), while the shorter isoform shows gonad (pink) and neuron (purple) accessibility. Again, the embryo RNA-seq supports the sci-ATAC-seq data, with greater neuron signal over the short isoform exons. (*C*) By scRNA-seq, gene C30F2.2 is mostly expressed in pharynx (blue) and neurons(purple), with some expression in intestine (yellow). There is prominent pharyngeal and intestine accessibility over the start of the long isoform, and a neuron peak over the short isoform. By scRNA-seq, gene C30F2.3 on the opposite strand is expressed in pharynx, with very little intestine or neuron expression. There is also an intriguing complex pattern of accessibility and RNA-seq signal downstream of C30F2.3, giving one example of the complexity captured in these data.

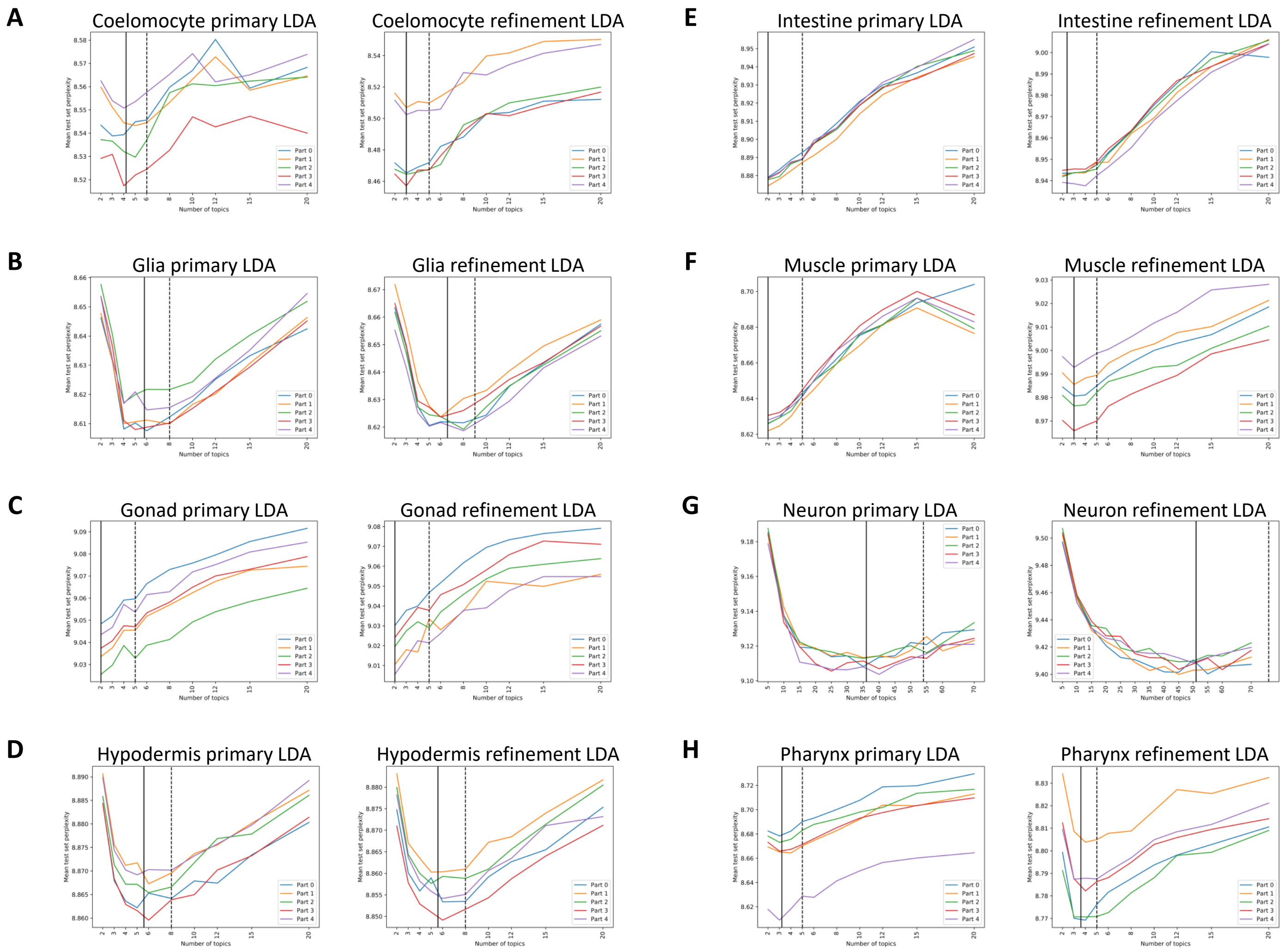

Supplementary Figure S13: **Tuning the number of topics using 5-fold cross validation.** Models were trained on 4 folds and tested on a held-out fold for varying numbers of topics. The average minimum topic number (solid line) was calculated, and used as the basis to pick a number of topics 1.5 times greater for use in training the full LDA model (dotted line). Pairs of plots show the topic number search for the tissue-specific primary LDA (left plot) and tissue-specific refinement LDA (right plot) for (*A*) coelomocyte, (*B*) glia, (*C*) gonad, (*D*) hypodermis, (*E*) intestine, (*F*) muscle, (*G*) neuron, (*H*) pharynx.

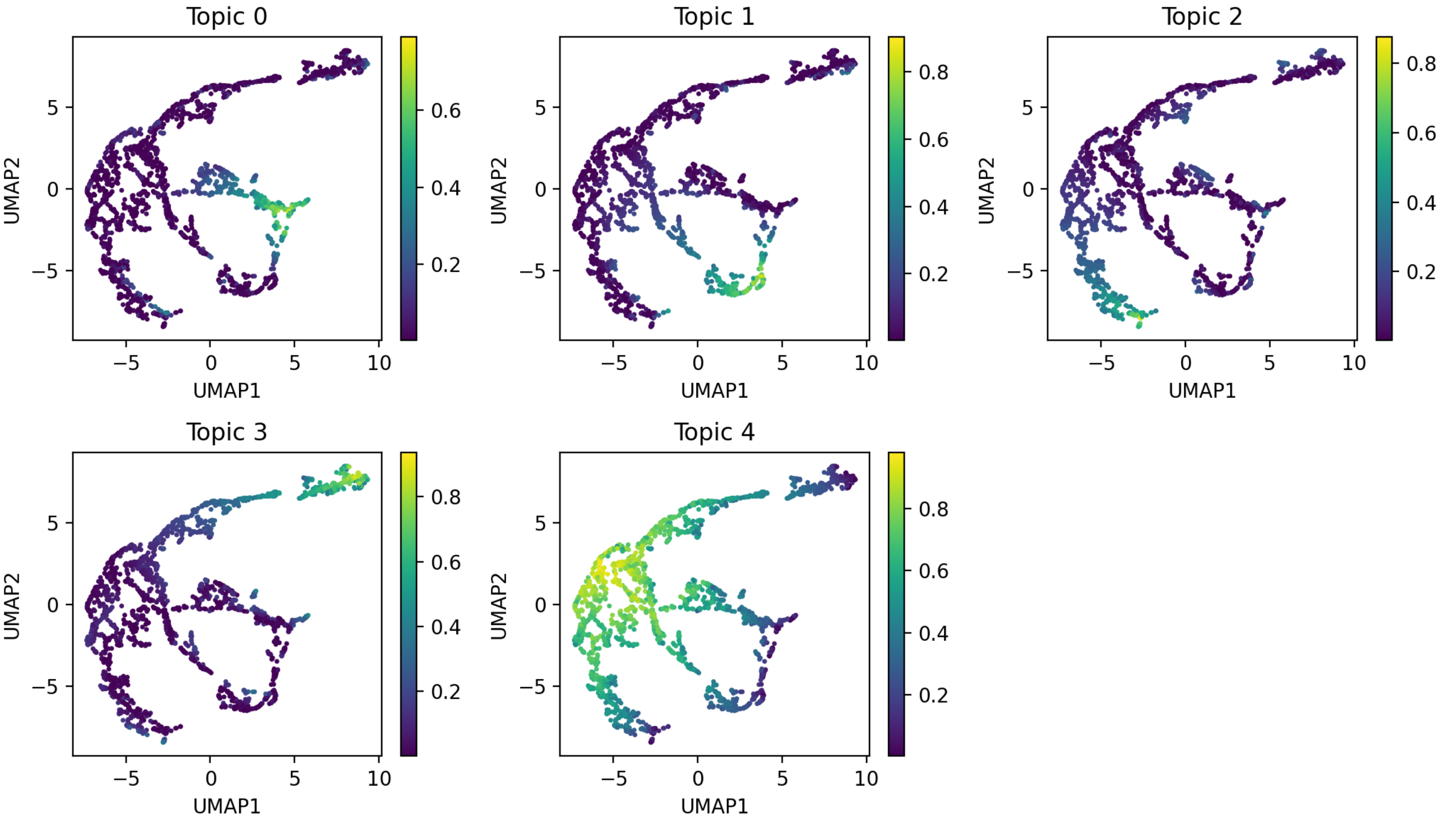

Supplementary Figure S14: **Topic probabilities for muscle subclustering LDA analysis.** UMAP plots displaying the results of performing our iterative LDA procedure on only muscle cells (topic 40 in the whole worm refinement LDA, see Fig. S9). Each dot in the scatterplot represents one cell, and in each plot the cells are colored by their probability for each LDA topic.

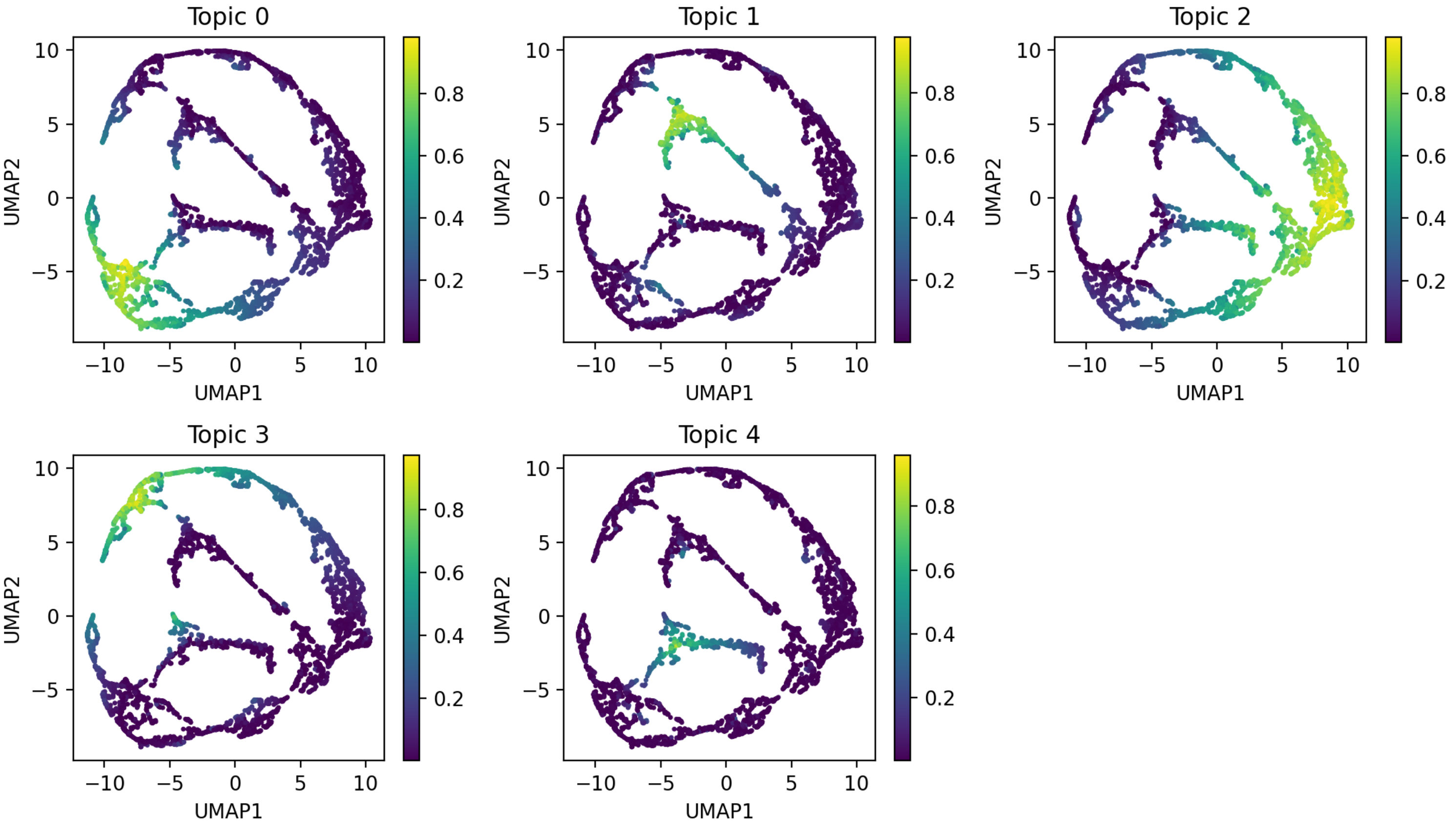

Supplementary Figure S15: **Topic probabilities for intestine subclustering LDA analysis.** UMAP plots displaying the results of performing our iterative LDA procedure on only intestine cells (topics 13, 23, 47, and 51 in the whole worm refinement LDA, see Fig. S9). Each dot in the scatterplot represents one cell, and in each plot the cells are colored by their probability for each LDA topic.

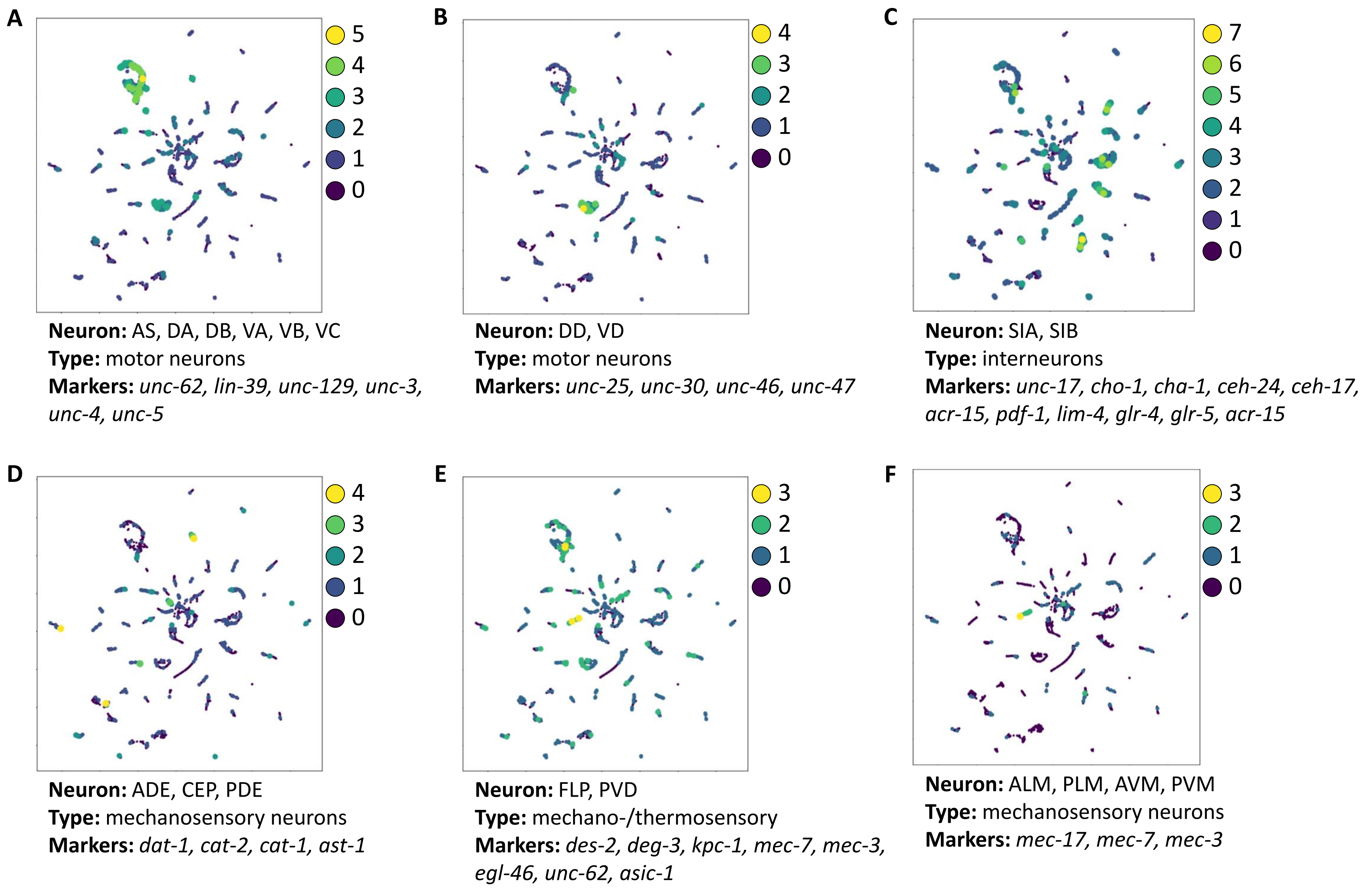

Supplementary Figure S16: **Marker genes identify neuronal types at high resolution.** We identified additional neurons in the UMAP plot by plotting the distribution of cells showing peaks of accessibility within 1200 bp upstream or 100 bp downstream of sets of marker genes from Packer, et al. 2019 (Packer et al., 2019). Each scatter plot dot represents a cell, and the number of genes with nearby accessibility in a given cell is shown by the color and size of its dot on the scatter plot, with cells showing accessibility near more marker genes having dots that are larger and more yellow. Information below each plot details the names of the neurons being highlighted, the type of neuron, and the marker genes used to generate the plot.

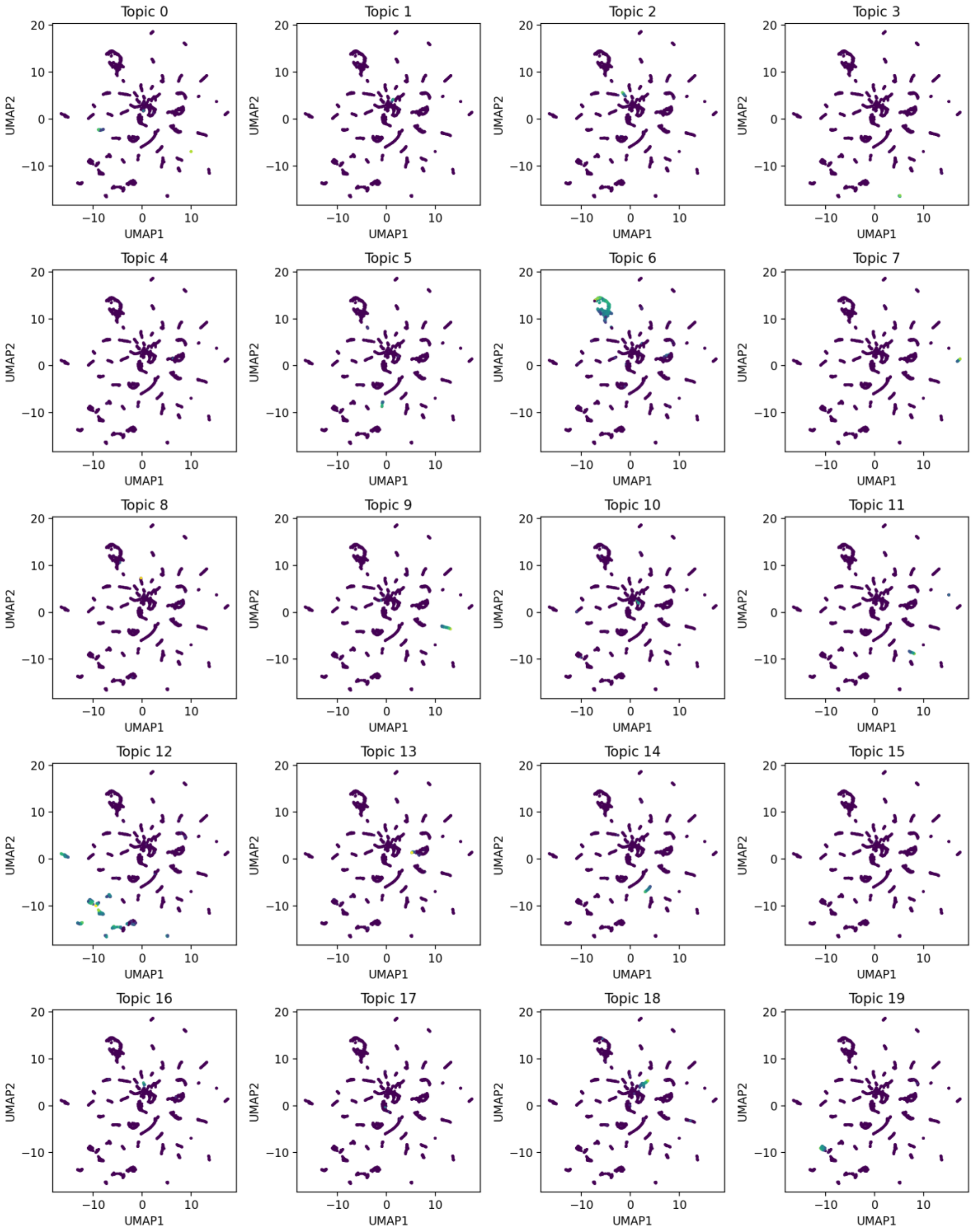

Supplementary Figure S17: **Topic probabilities for neuron subclustering LDA analysis. Part 1.** UMAP plots displaying the results of performing our iterative LDA procedure on only neuron cells (topics 0, 6, 14, 15, 16, 18, 19, 32, 33, 38, and 45 in the whole worm refinement LDA, see Fig. S9). Each dot in the scatterplot represents one cell, and in each plot the cells are colored by their probability for each LDA topic.

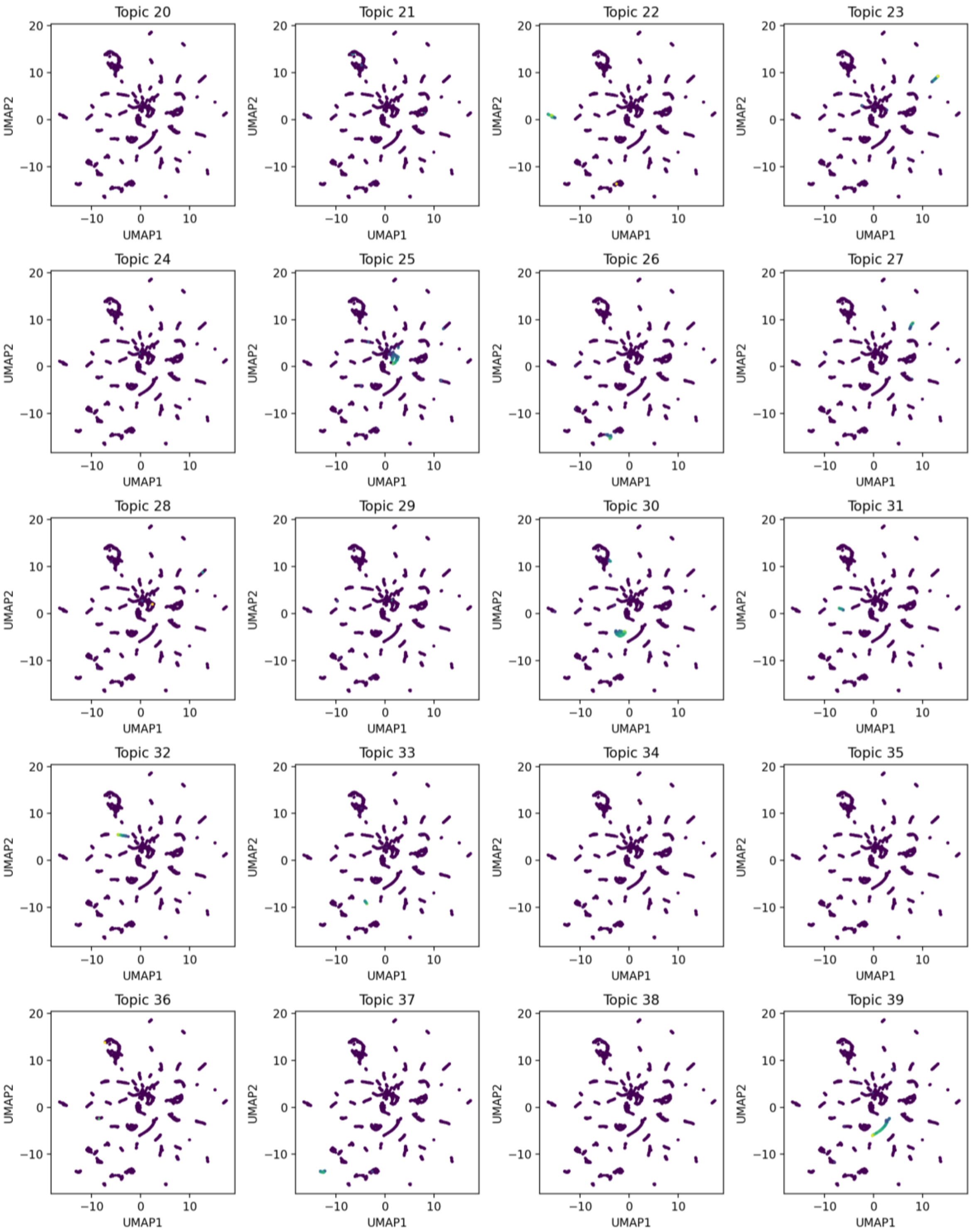

Supplementary Figure S18: **Topic probabilities for neuron subclustering LDA analysis. Part 2.** UMAP plots displaying the results of performing our iterative LDA procedure on only neuron cells (topics 0, 6, 14, 15, 16, 18, 19, 32, 33, 38, and 45 in the whole worm refinement LDA, see Fig. S9). Each dot in the scatterplot represents one cell, and in each plot the cells are colored by their probability for each LDA topic.

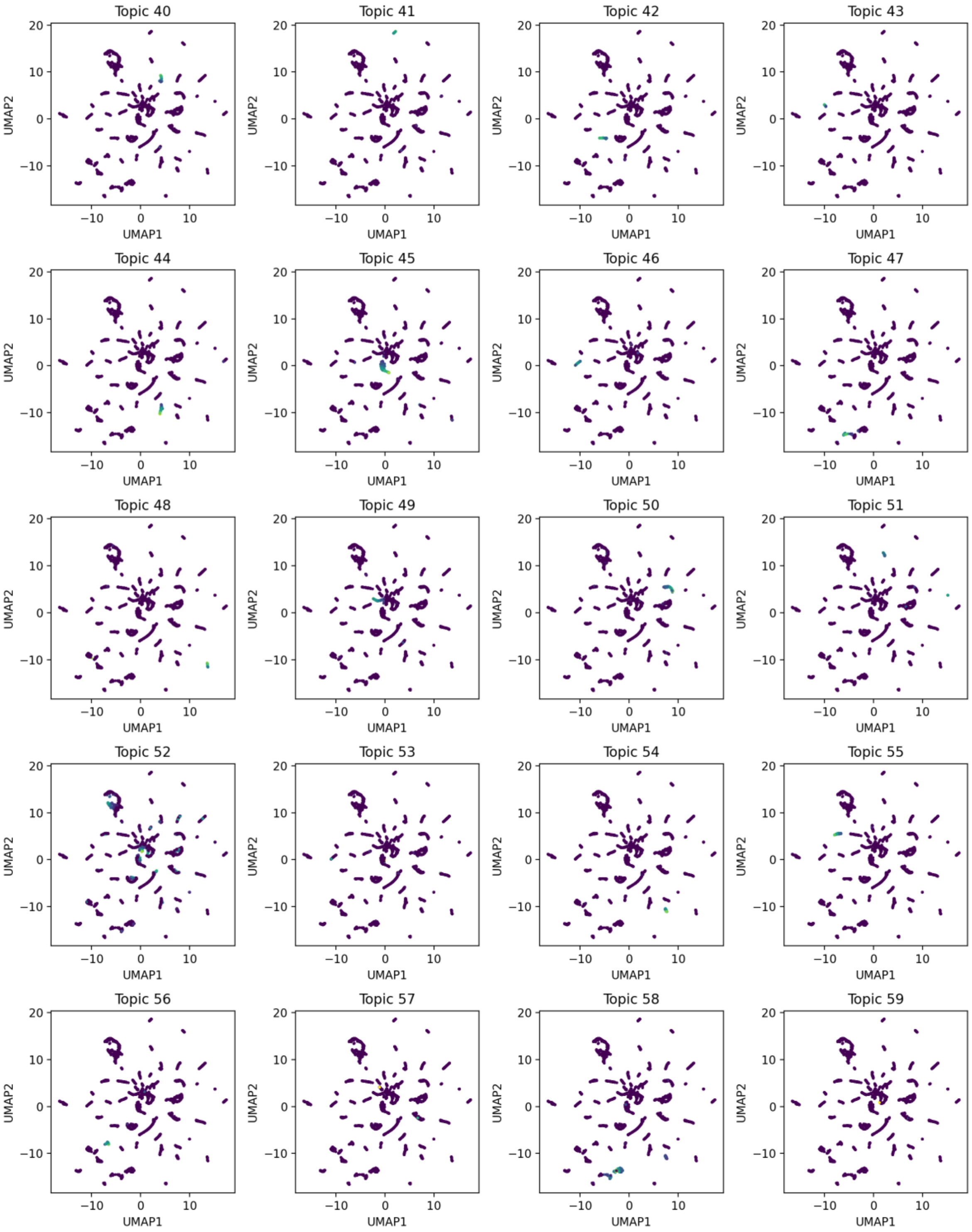

Supplementary Figure S19: **Topic probabilities for neuron subclustering LDA analysis. Part 3.** UMAP plots displaying the results of performing our iterative LDA procedure on only neuron cells (topics 0, 6, 14, 15, 16, 18, 19, 32, 33, 38, and 45 in the whole worm refinement LDA, see Fig. S9). Each dot in the scatterplot represents one cell, and in each plot the cells are colored by their probability for each LDA topic.

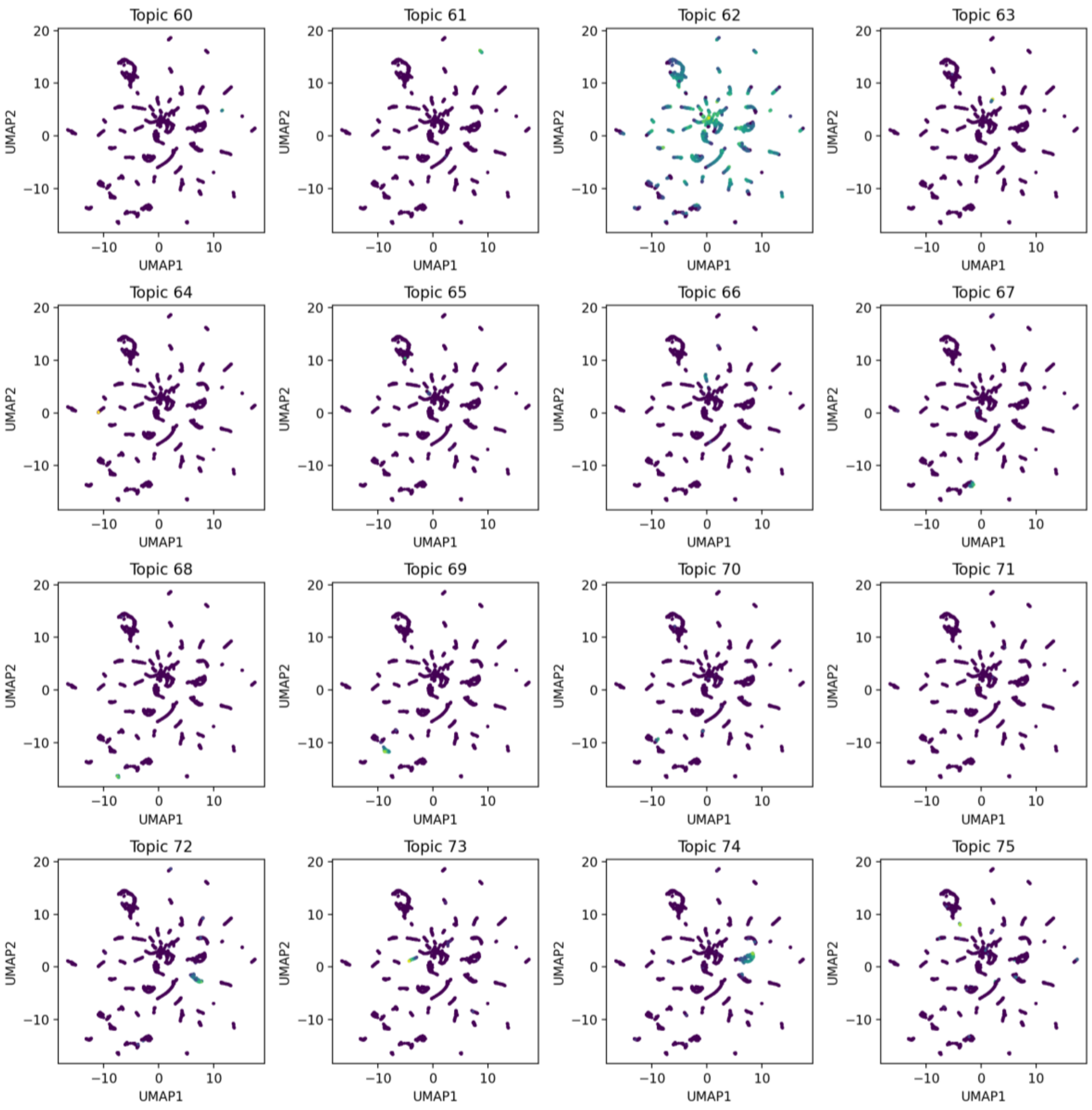

Supplementary Figure S20: **Topic probabilities for neuron subclustering LDA analysis. Part 4.** UMAP plots displaying the results of performing our iterative LDA procedure on only neuron cells (topics 0, 6, 14, 15, 16, 18, 19, 32, 33, 38, and 45 in the whole worm refinement LDA, see Fig. S9). Each dot in the scatterplot represents one cell, and in each plot the cells are colored by their probability for each LDA topic.

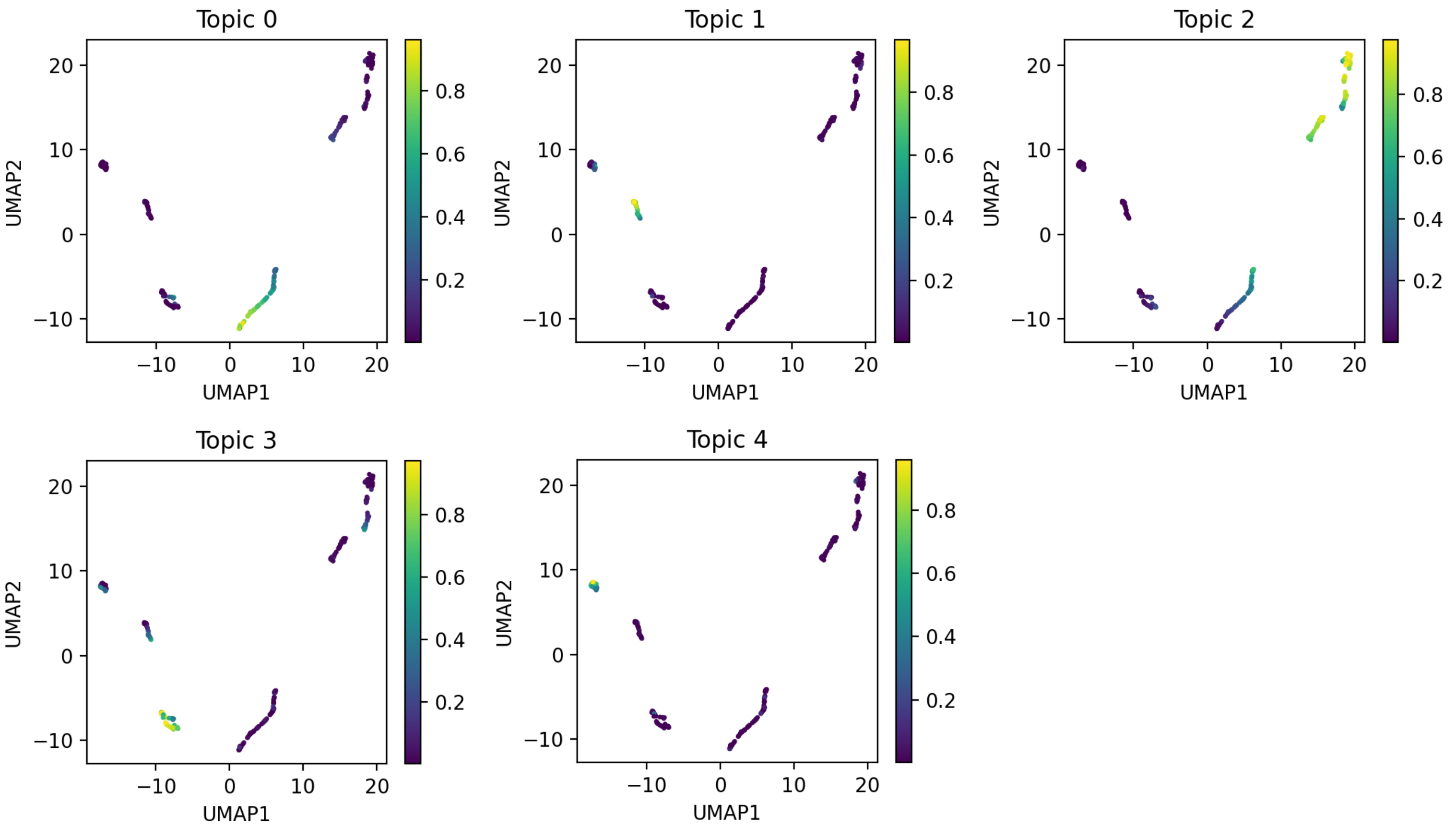

Supplementary Figure S21: **Topic probabilities for coelomocyte subclustering LDA analysis.** UMAP plots displaying the results of performing our iterative LDA procedure on only coelomocyte cells (topic 4 in the whole worm refinement LDA, see Fig. S9). Each dot in the scatterplot represents one cell, and in each plot the cells are colored by their probability for each LDA topic.

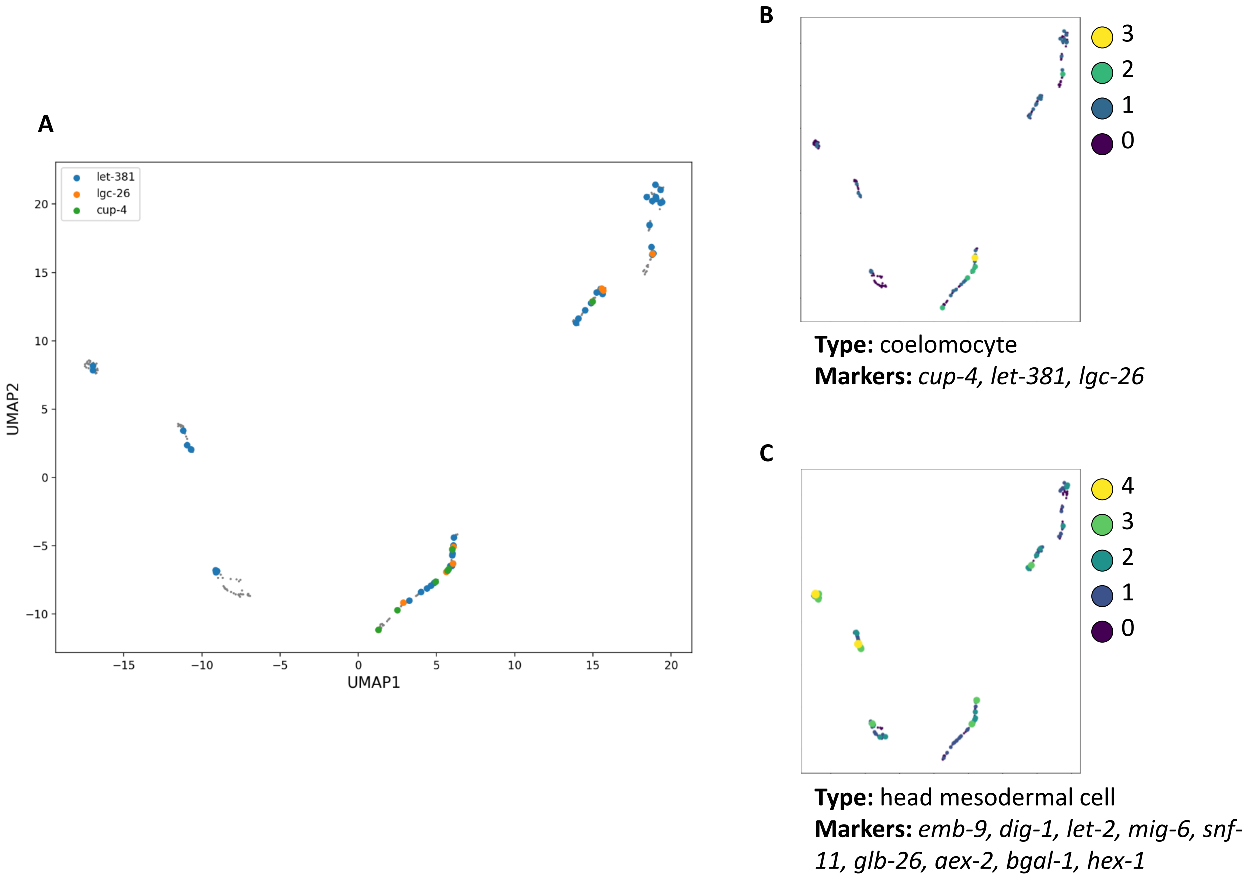

Supplementary Figure S22: **Marker genes identify coelomocyte subclusters.** We identified coelomocyte subclusters in the UMAP plot by plotting the distribution of cells showing peaks of accessibility within 1200 bp upstream or 100 bp downstream of sets of marker genes from Packer, et al. 2019 (Packer et al., 2019). (*A*) Scatter plot of the UMAP embedding with the cells colored by which of three coelomocyte marker genes show nearby accessibility. (*B*) The same coelomocyte marker genes are plotted, but in this case each cell is colored based on how many of the marker genes show nearby accessibility in each cell. (*C*) Plotting the number of head mesodermal cell marker genes with nearby accessibility identifies the clusters enriched for topics 1 and 4 (Fig. S21) as candidate head mesodermal cells.

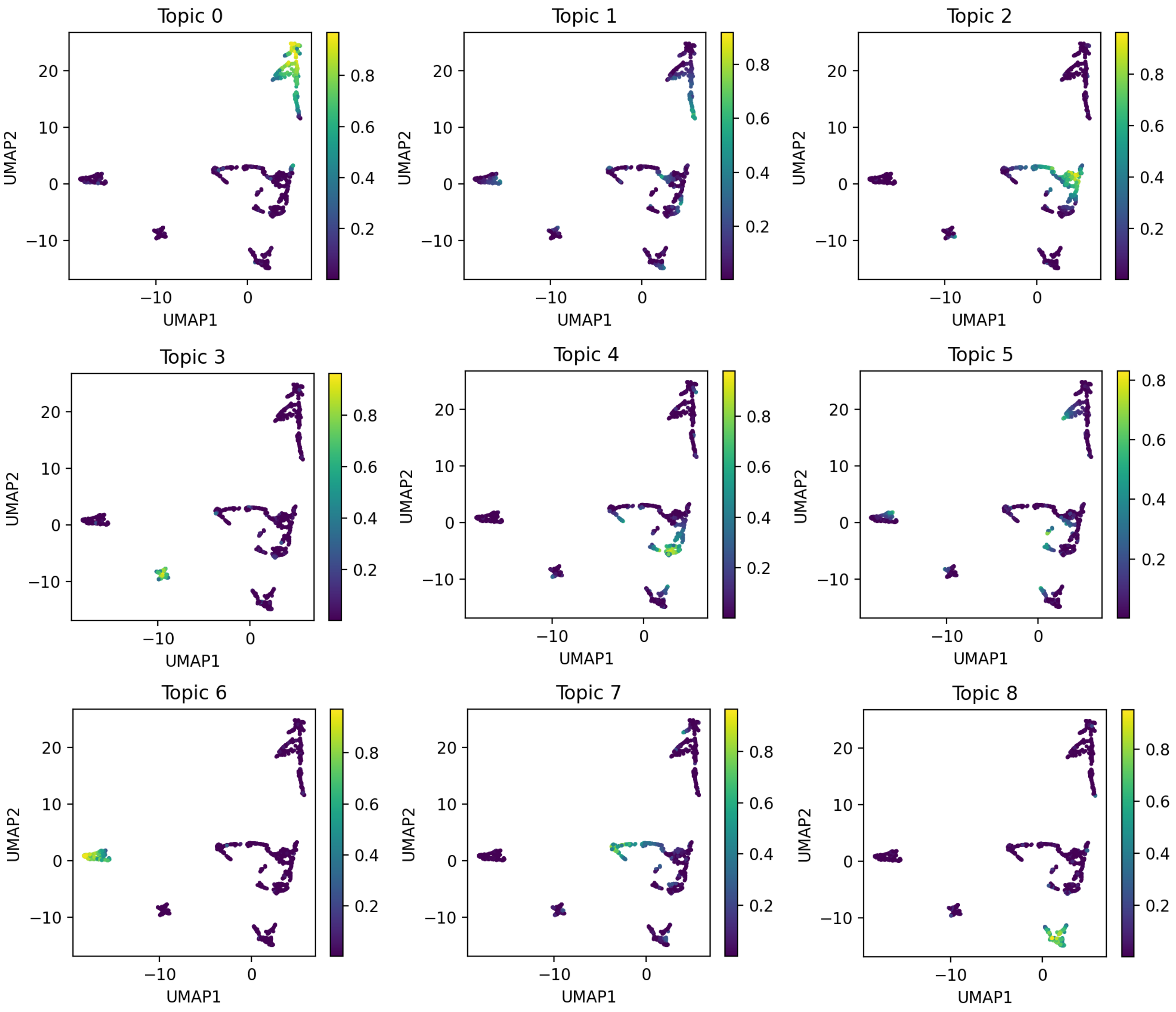

Supplementary Figure S23: **Topic probabilities for glia subclustering LDA analysis.** UMAP plots displaying the results of performing our iterative LDA procedure on only glial cells (topics 12, 21, 27, and 31 in the whole worm refinement LDA, see Fig. S9). Each dot in the scatterplot represents one cell, and in each plot the cells are colored by their probability for each LDA topic.

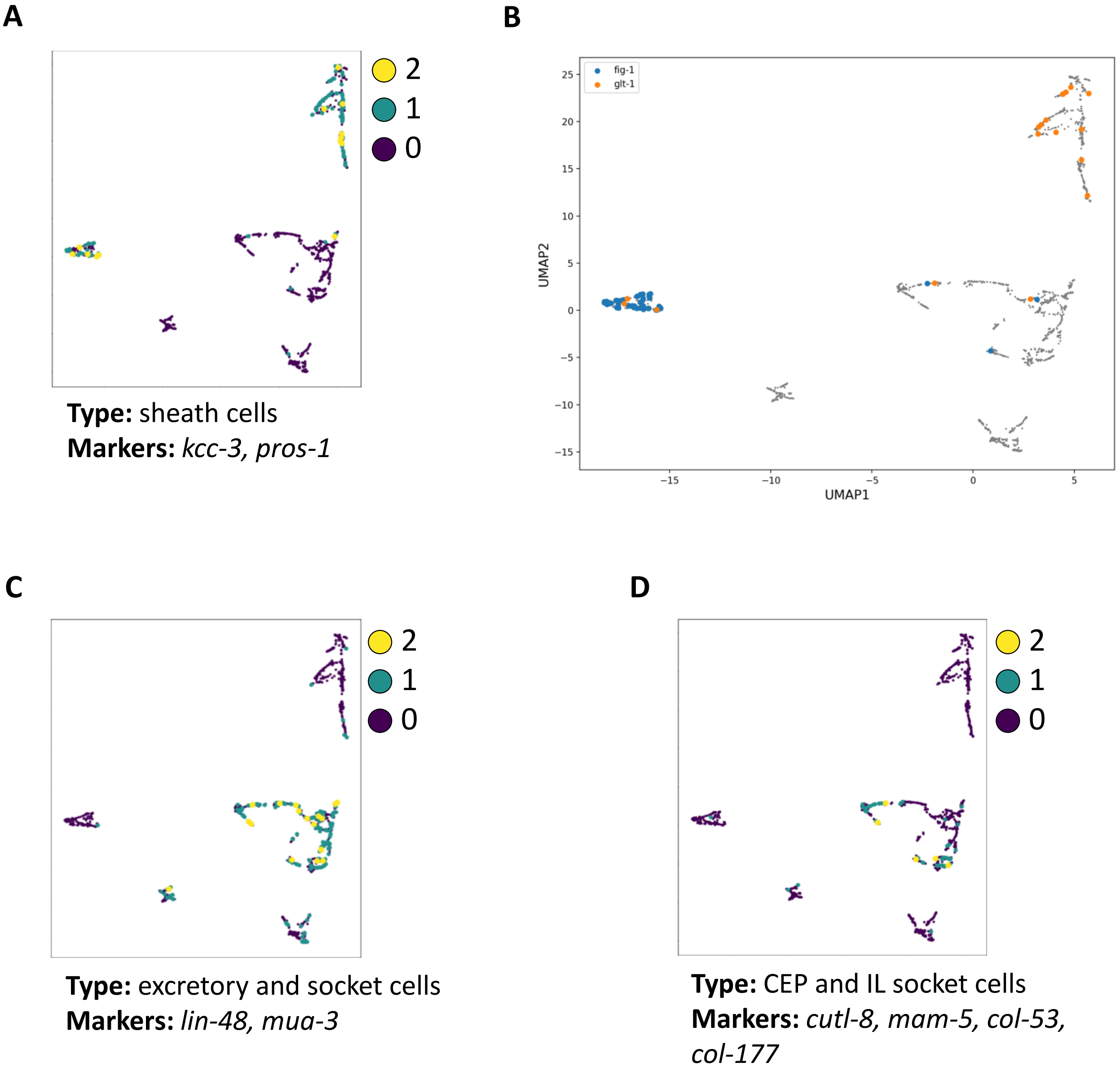

Supplementary Figure S24: **Marker genes identify glia subclusters.** We identified glia subclusters by plotting the distribution of cells showing accessibility for sets of marker genes from Packer, et al. 2019 (Packer et al., 2019). (*A*) Sheath cells are characterized by expression of marker genes *kcc-3* and *pros-1*. Cells that show accessibility near these genes are predominantly those with high probabilities for topics 0 and 6 (Fig. S23). (*B*) The sheath cells can be further subdivided based on the expression of *fig-1*, which marks amphid and phasmid sheath cells, and *glt-1*, which marks cephalic sheath cells. The cells with high probability for topic 0 (Fig. S23) have low accessibility near *fig-1*, but do show accessibility near *glt-1*, identifying them as candidate cephalic sheath cells, while cells with high probability in topic 6 (Fig. S23) show the reverse and are candidate amphid and phasmid sheath cells. (*C*) Similarly, coloring the cells by accessibility nearby *lin-48* and *mua-3* show the other cells in the plot are candidate excretory and socket cells, while accessibility near marker genes *cutl-8*, *mam-5*, *col-53*, and *col-177* suggest that the cells with high topic 4 and topic 7 probability (Fig. S23) are candidate cephalic and inner labial socket cells (*D*).

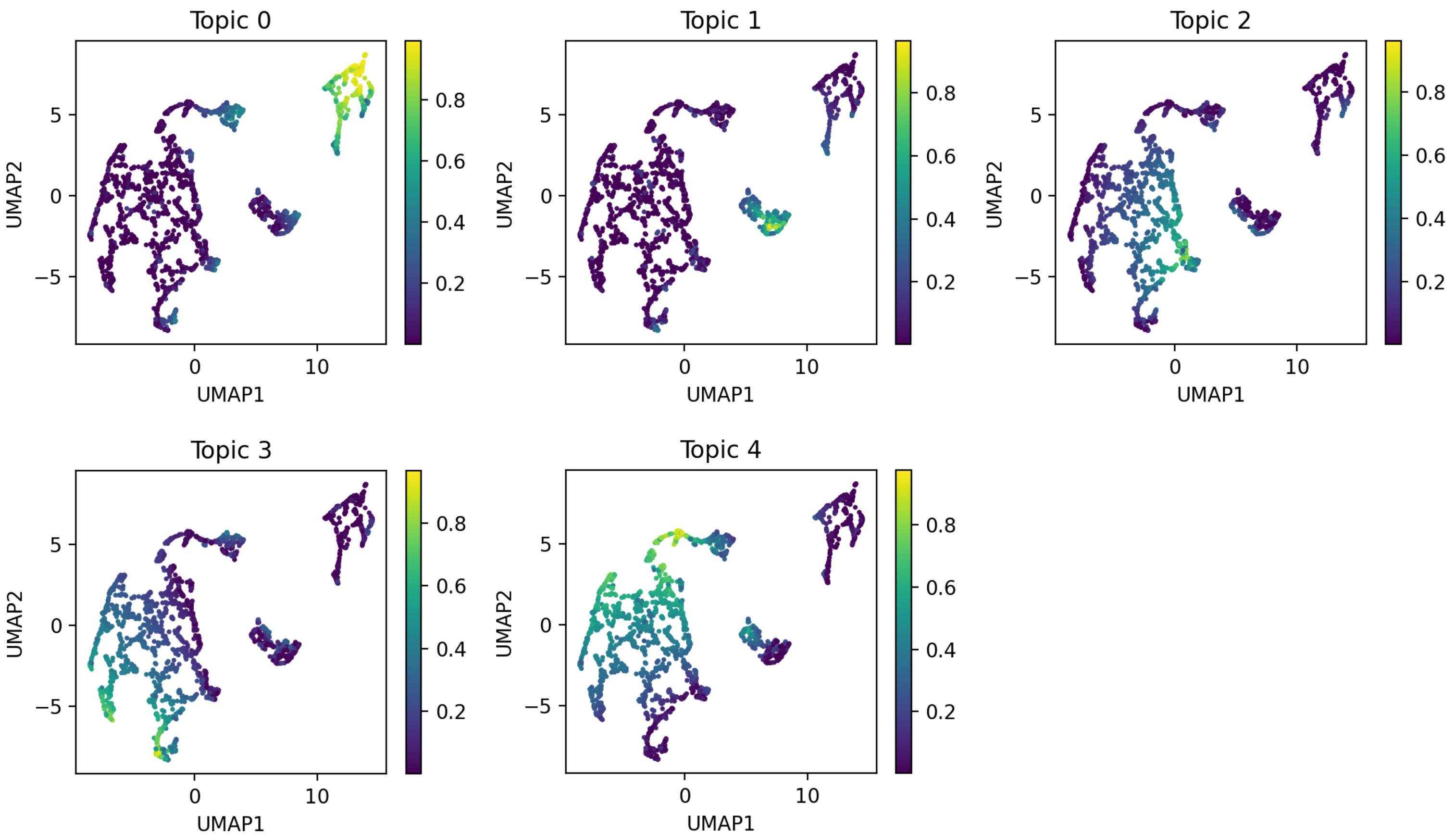

Supplementary Figure S25: **Topic probabilities for gonad subclustering LDA analysis.** UMAP plots displaying the results of performing our iterative LDA procedure on only gonad cells (topics 7, 24, 36, and 48 in the whole worm refinement LDA, see Fig. S9). Each dot in the scatterplot represents one cell, and in each plot the cells are colored by their probability for each LDA topic.

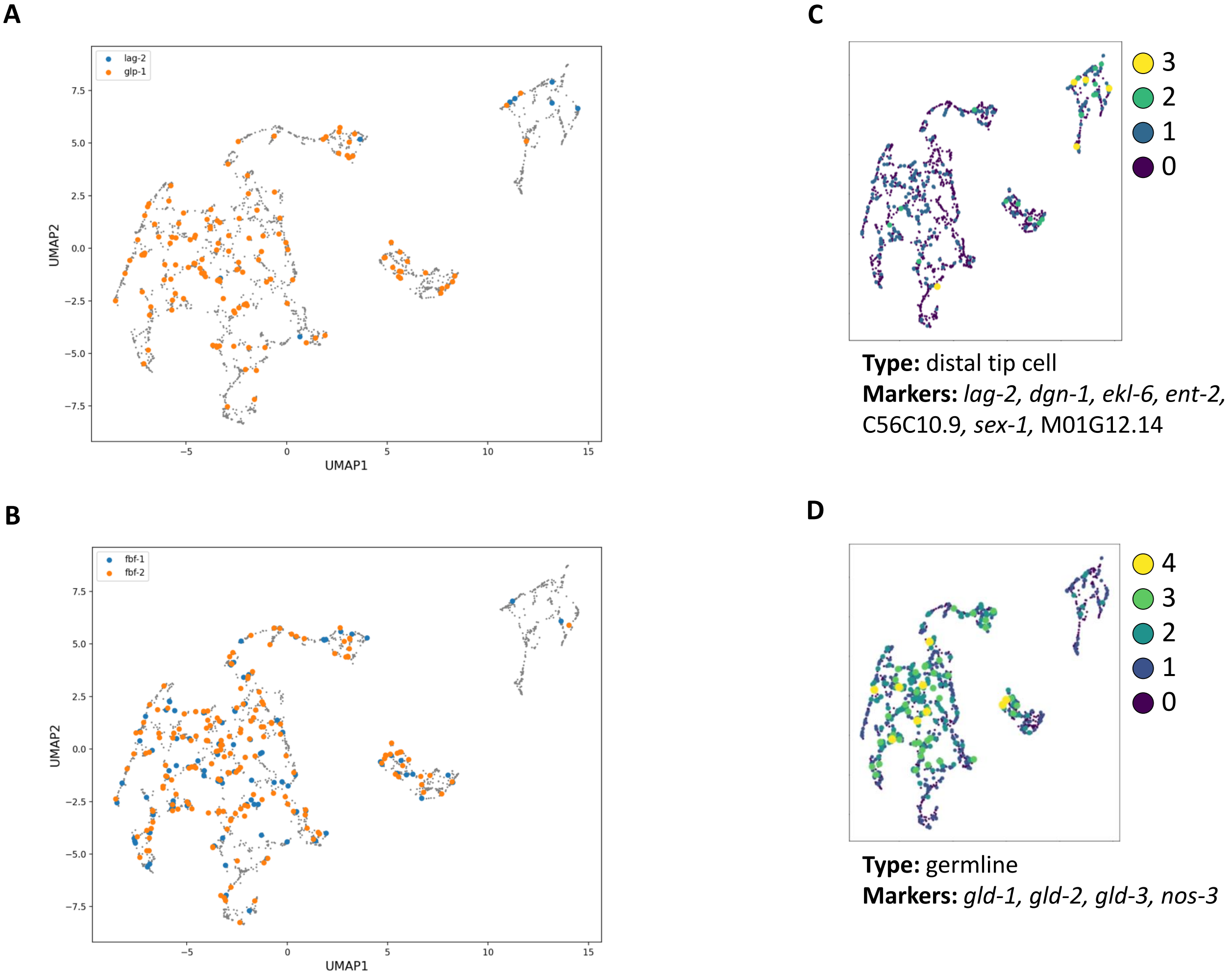

Supplementary Figure S26: **Marker genes identify gonad subclusters.** We identified gonad subclusters by plotting the distribution of cells showing accessibility for sets of marker genes from Packer, et al. 2019 (Packer et al., 2019) and Wormbook (Kimble, 2005). The gonad forms with a stem cell niche maintained by the distal tip cells that maintain stemness in the germline by Notch signaling. The distal tip cells produce the Notch ligand LAG-2, while the mitotic germline cells express the receptor, GLP-1. Here, the gonad LDA analysis largely separates the cells with accessibility near these two genes (*A*), suggesting that the cells with high topic 0 probability (Fig. S25) are candidate distal tip cells, while most of the others are candidate germline cells. This observation is also supported by looking for accessibility near the *fbf-1* and *fbf-2* genes (*B*), which encode RNA binding proteins that function downstream of GLP-1 to maintain germ cells in the mitotic state. The candidate distal tip cells also show coaccessibility near other distal tip cell marker genes identified from the single cell RNA-seq data (*C*), and similarly, additional germline marker genes show nearby coaccessibility in the same cells that have accessible sites near the *fbf* genes (*D*).

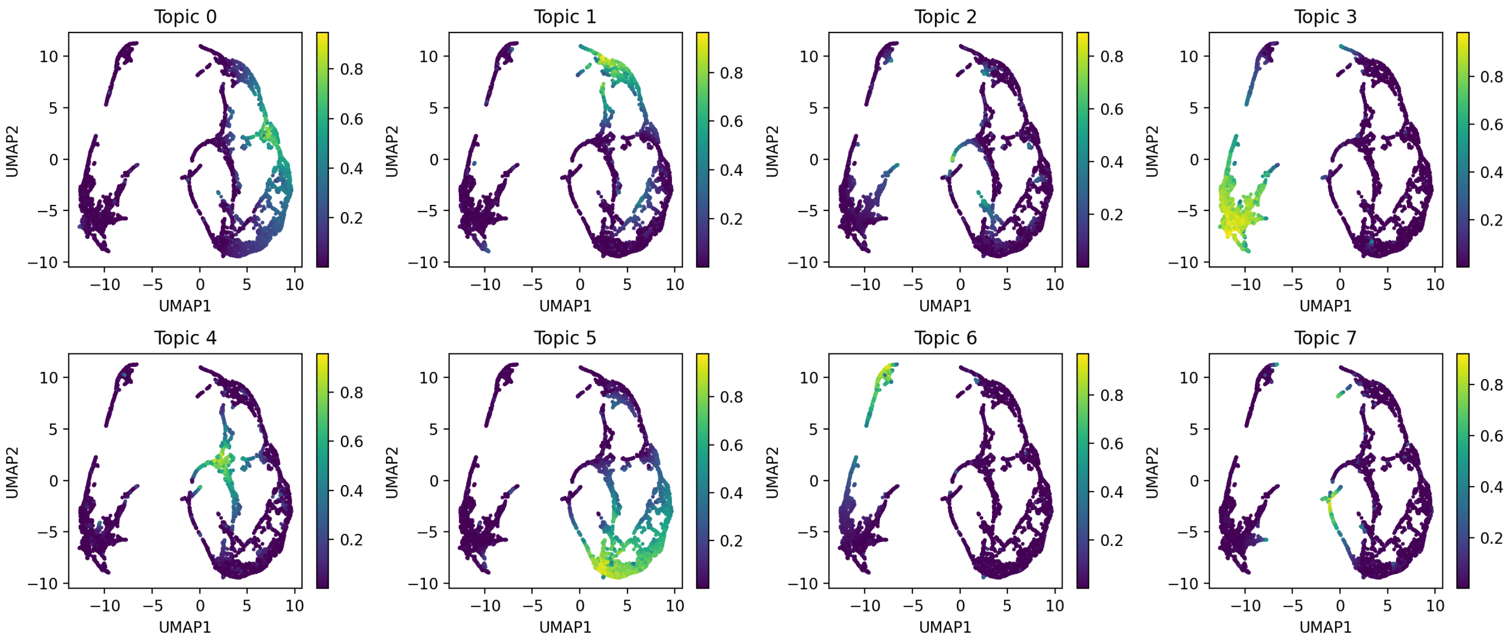

Supplementary Figure S27: **Topic probabilities for hypodermis subclustering LDA analysis.** UMAP plots displaying the results of performing our iterative LDA procedure on only hypodermal cells (topics 1, 9, 10, 17, 25, 30, 41, 43, and 46 in the whole worm refinement LDA, see Fig. S9). Each dot in the scatterplot represents one cell, and in each plot the cells are colored by their probability for each LDA topic.

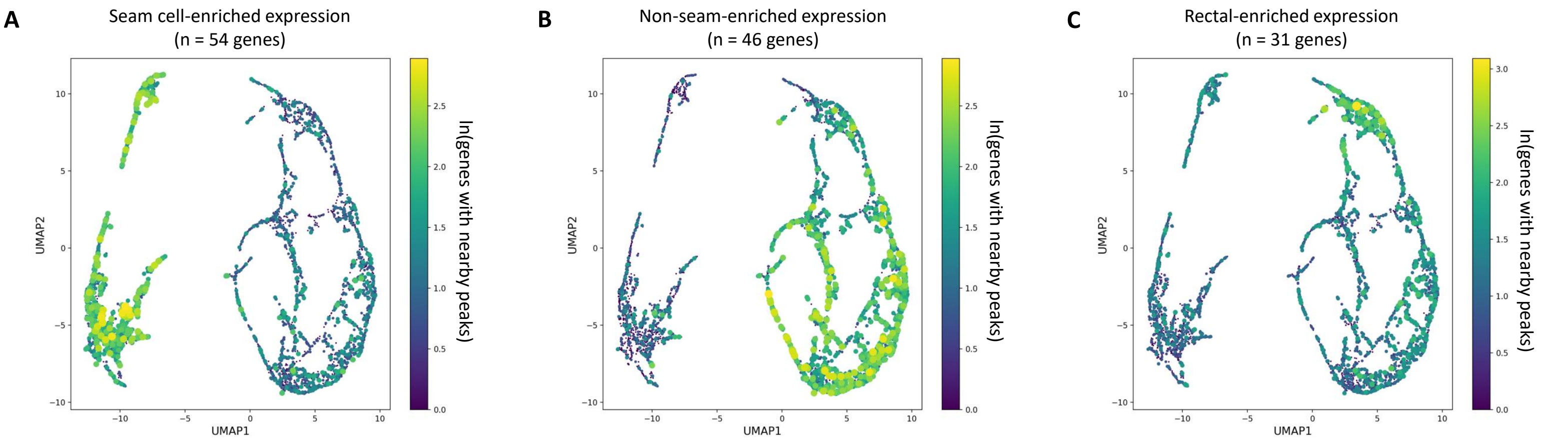

Supplementary Figure S28: **Marker genes identify hypodermis subclusters.** To identify hypodermis subclusters, we assessed co-accessibility of sites near genes enriched in expression for the hypodermal tissues identified in Cao et al. 2017 (Cao et al., 2017). The genes that we selected have greater than five-fold enrichment in the specified hypodermis tissue compared to all other tissues, as reported by the GExplore website (http://genome.sfu.ca/gexplore/gexplore_search_tissues.html). (*A*) The cells with high probability in topics 3 and 6 (Fig. S27) show high co-accessibility of regions near genes with enriched expression in seam cells. (*B*) Genes with enriched expression in non-seam hypodermis have nearby co-accessible sites in cells with high probability in topics 0, 4, 5, and 7 (Fig. S27). (*C*) Last, cells with high probability for topic 1 (Fig. S27) tend to have co-accessible sites near genes with enriched expression in rectum.

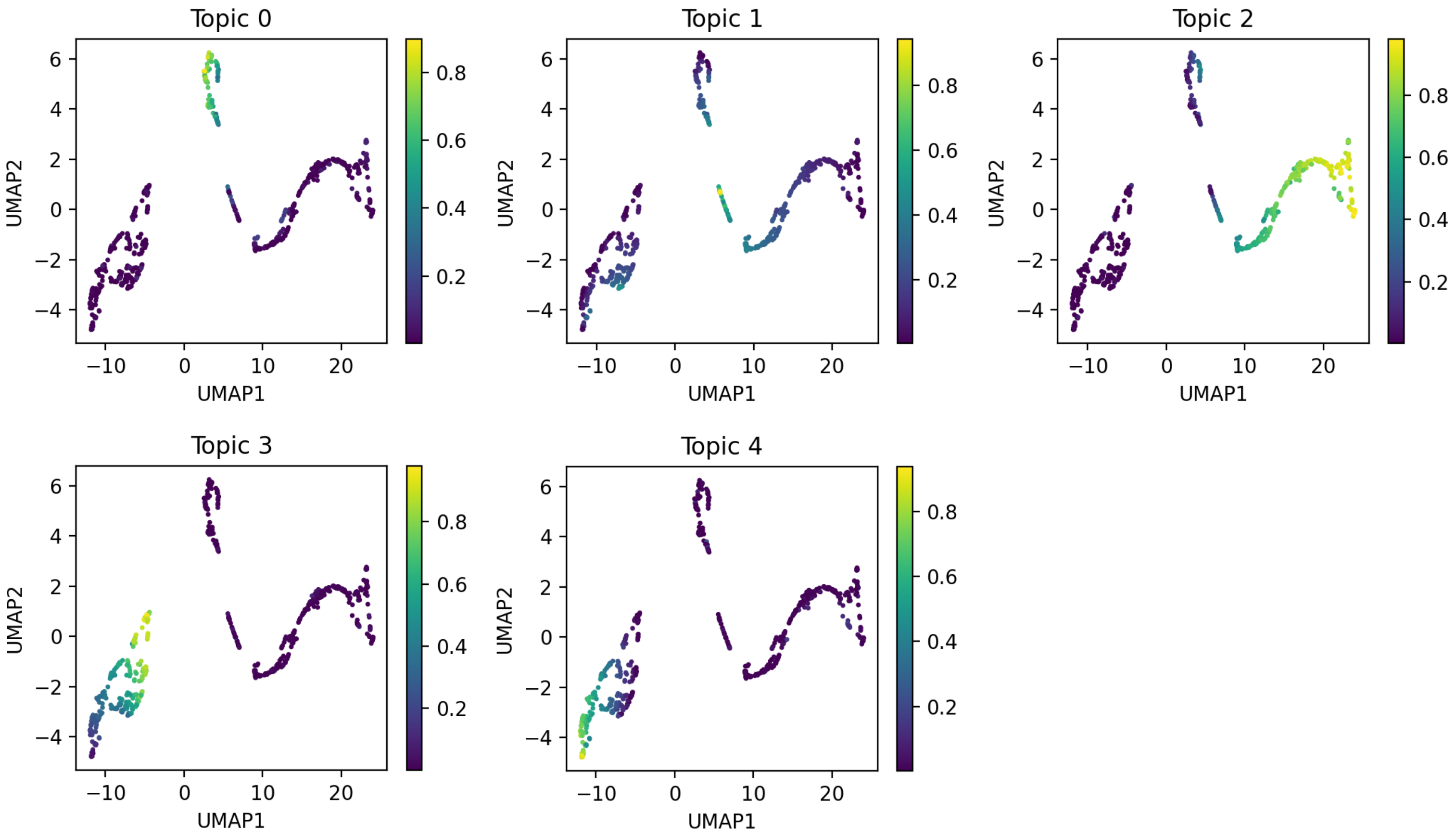

Supplementary Figure S29: **Topic probabilities for pharynx subclustering LDA analysis.** UMAP plots displaying the results of performing our iterative LDA procedure on only pharyngeal cells (topics 35 and 53 in the whole worm refinement LDA, see Fig. S9). Each dot in the scatterplot represents one cell, and in each plot the cells are colored by their probability for each LDA topic.

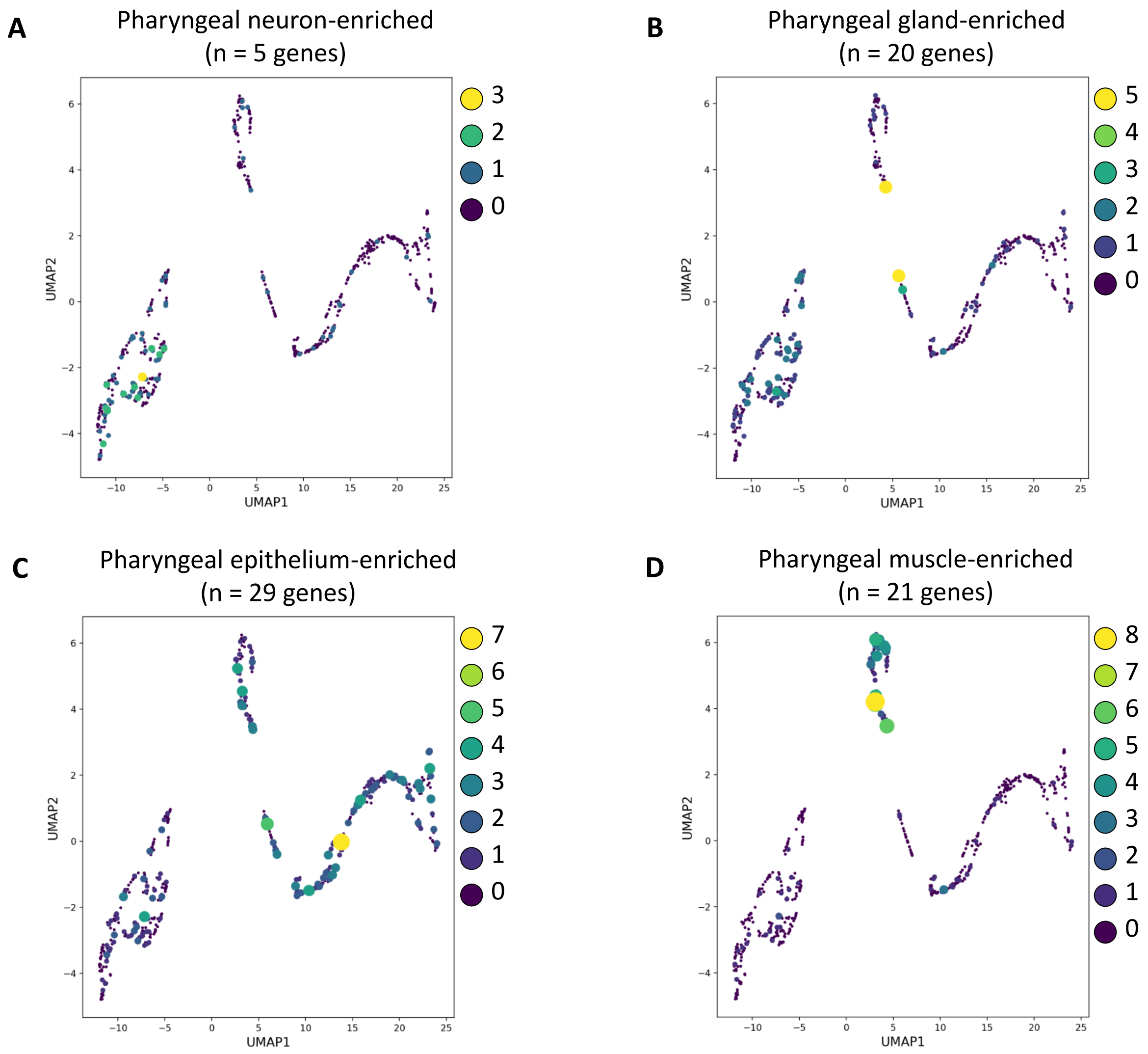

Supplementary Figure S30: **Marker genes identify pharynx subclusters.** To identify pharynx subclusters, we assessed co-accessibility of sites near genes enriched in expression in the pharyngeal tissues identified in Cao et al. 2017 (Cao et al., 2017). The genes that we selected have greater than five-fold enrichment in the specified pharyngeal tissue compared to all other tissues, as reported by the GExplore website (http://genome.sfu.ca/gexplore/gexplore_search_tissues.html). Note that a relatively small subset of the genes matching the expression criteria in these tissues have nearby peaks, probably because our experiment recovered relatively few pharyngeal cells, reducing our power to detect pharynx-specific peaks. Nevertheless, we find that the genes with enriched expression in different pharyngeal tissues show nearby co-accessibility in cells with high probability in different topics. In particular, cells with high probability for topics 3 and 4 (Fig. S29) have more accessibility near genes expressed in pharyngeal neurons (*A*), cells with high probability in topic 1 (Fig. S29) have more accessibility near genes expressed in pharyngeal gland (*B*), cells with high probability in topic 2 (Fig. S29) have more accessibility near genes expressed in pharyngeal epithelium (*C*), and cells with high probability in topic 0 (Fig. S29) have more accessibility near genes expressed in pharyngeal muscle (*D*).

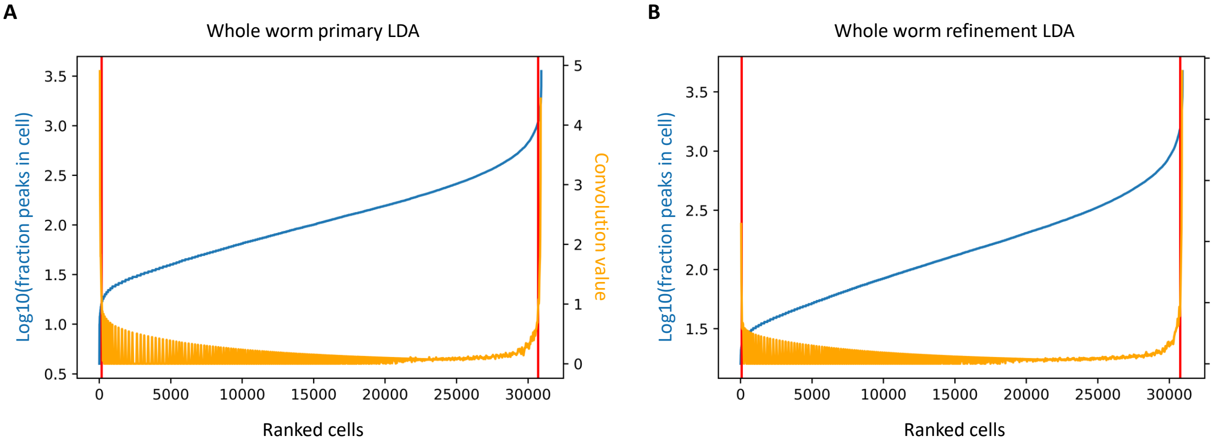

Supplementary Figure S31: **Filtering cells with too few peaks.** Cells were ranked by the number of peaks detected (blue line), and cells with too few peaks were filtered out. The threshold (left-hand red vertical line) was determined by automatically finding the inflection point in the ranking curve (orange line). (*A*) Filtering cells before the whole-worm primary LDA iteration. (*B*) Filtering cells before the whole-worm refinement LDA iteration.

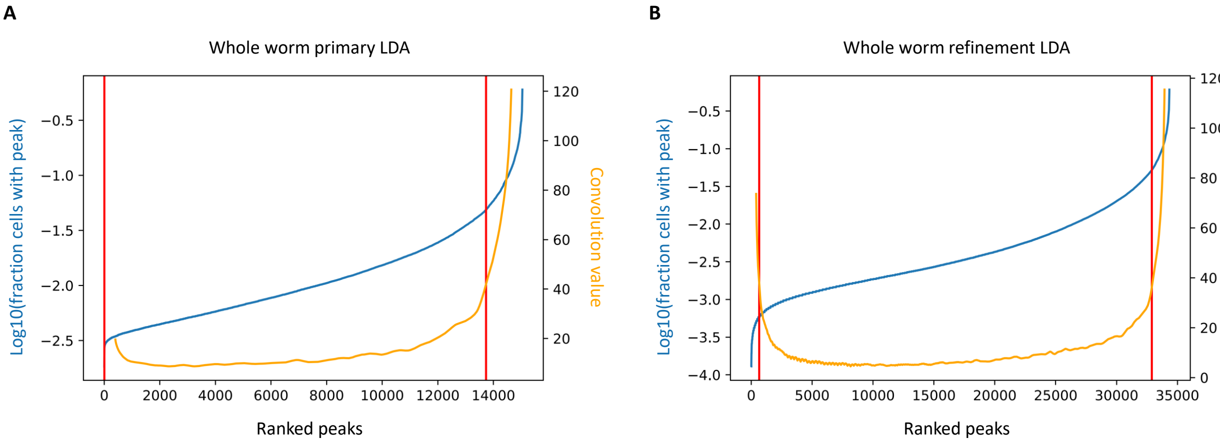

Supplementary Figure S32: **Filtering peaks found in too many or too few cells.** Peaks were ranked by the fraction of cells in which they were detected (blue line), and outlier peaks were filtered out. The thresholds (red vertical lines) were determined by automatically finding the inflection points in the ranking curve (orange line). (*A*) Filtering peaks before the whole-worm primary LDA iteration. (*B*) Filtering peaks before the whole-worm refinement LDA iteration.
